# Asymmetric Evolution and Expansion of the NAC Transcription Factor in Polyploidized Cotton

**DOI:** 10.3389/fpls.2018.00047

**Published:** 2018-01-30

**Authors:** Kai Fan, Feng Li, Jiahuan Chen, Zhaowei Li, Weiwei Lin, Size Cai, Jianping Liu, Wenxiong Lin

**Affiliations:** ^1^Key Laboratory of Ministry of Education for Genetics, Breeding and Multiple Utilization of Crops, College of Crop Science, Fujian Agriculture and Forestry University, Fuzhou, China; ^2^Fujian Provincial Key Laboratory of Agroecological Processing and Safety Monitoring, College of Life Sciences, Fujian Agriculture and Forestry University, Fuzhou, China; ^3^Key Laboratory of Crop Ecology and Molecular Physiology (Fujian Agriculture and Forestry University), Fujian Province University, Fuzhou, China; ^4^College of Life Science, Shanxi Datong University, Datong, China

**Keywords:** cotton, NAC family, molecular evolution, expansion, polyploidization

## Abstract

Polyploidy in *Gossypium hirsutum* conferred different properties from its diploid ancestors under the regulation of transcription factors. The NAC transcription factor is a plant-specific family that can be related to plant growth and development. So far, little is known about the NAC family in cotton. This study identified 495 NAC genes in three cotton species and investigated the evolution and expansion of different genome-derived NAC genes in cotton. We revealed 15 distinct NAC subfamilies in cotton. Different subfamilies had different gene proportions, expansion rate, gene loss rate, and orthologous exchange rate. Paleohexaploidization (35%) and cotton-specific decaploidy (32%) might have primarily led to the expansion of the NAC family in cotton. Half of duplication events in *G. hirsutum* were inherited from its diploid ancestor, and others might have occurred after interspecific hybridization. In addition, NAC genes in the At and Dt subgenomes displayed asymmetric molecular evolution, as evidenced by their different gene loss rates, orthologous exchange, evolutionary rates, and expression levels. The dominant duplication event was different during the cotton evolutionary history. Different genome-derived NACs might have interacted with each other, which ultimately resulted in morphogenetic evolution. This study delineated the expansion and evolutionary history of the NAC family in cotton and illustrated the different fates of NAC genes during polyploidization.

## Introduction

Cotton is a major economic crop that serves as a principal source of natural fiber and a raw material of oil. Cotton is also an ideal model plant for research on polyploidization. The *Gossypium* genus experienced two major events. In cotton ancestry, the A-genome diploids diverged from the D-genome diploids ~5–10 million years ago (MYA). Afterward, allopolyploid *Gossypium* species, including *G. hirsutum*, formed through the interspecific hybridization between the A-genome ancestor resembling *G. arboreum* and the D-genome ancestor resembling *G. raimondii* around 1–2 MYA (Li et al., 2015; Zhang et al., 2015). Cotton allopolyploidization produces thousands of duplicated genes with different expression levels (Hu et al., 2015; Wang et al., 2016). The allopolyploid cotton species *G. hirsutum* differs greatly from the putative donor species *G. arboreum* and *G. raimondii* in plant morphology and economic traits (Paterson et al., 2012; Li et al., 2014). Transcription factors play important regulatory roles in the aforementioned networks. Many transcription factors, such as TCP, WRKY, and MYB, regulate numerous critical biological processes in cotton (Hao et al., 2012; Yu et al., 2012; Lu et al., 2017).

The NAC (NAM, ATAF, and CUC) family is one of the largest families of plant-specific transcription factors (Ooka et al., 2003; Olsen et al., 2005). The first reported NAC gene (NAM) is related to the formation of the shoot apical meristem and primordium in Petunia (Souer et al., 1996). In general, the NAC family has a highly conserved N-terminal region (NAC domain) and a relatively divergent C-terminal transcriptional activation region (TAR) (Puranik et al., 2012). The NAC family regulates plant growth and development processes, including lateral root formation (Guo et al., 2005), leaf senescence (Guo and Gan, 2006), flower morphogenesis (Sablowski and Meyerowitz, 1998), cellular metabolism (Kim et al., 2009), seed development (Kim et al., 2008), fruit ripening (Shan et al., 2012), secondary wall synthesis (Mitsuda et al., 2005), and hormonal signaling (He et al., 2005). Moreover, NAC genes respond to many biotic and abiotic stresses, such as pathogen disease (Wang et al., 2009), drought (Mao et al., 2015), salt (Hu et al., 2006), and temperature (Fang et al., 2015). NAC transcription factors are also associated with crop yield and quality (Uauy et al., 2006; Liang et al., 2014; Zhao et al., 2015).

NAC genes have been studied in various plant species, such as *Arabidopsis thaliana* (Jensen et al., 2010), *Oryza sativa* (Nuruzzaman et al., 2010), *Zea mays* (Fan et al., 2014), *Glycine max* (Pinheiro et al., 2009), *Solanum tuberosum* (Singh et al., 2013), *Musa acuminata* (Shan et al., 2012), *Eucalyptus grandis* (Hussey et al., 2015), and *Vitis vinifera* (Wang et al., 2013). Several NAC genes have been isolated in cotton. GhNAP influences cotton yield and its fiber quality by regulating leaf senescence (Fan et al., 2015a), and GhATAF1 responds to salt stress and fungal infection by coordinating phytohormone signaling networks (He et al., 2016). However, genome-wide analysis of the NAC family in cotton is lacking.

The completion of genome sequencing in *G. arboreum, G. raimondii*, and *G. hirsutum* has opened an opportunity to investigate gene families in cotton (Wang et al., 2012; Li et al., 2014, 2015). A comprehensive analysis of the NAC family during allopolyploidization is expected to accelerate molecular breeding in cotton. Previous studies conducted whole-genome annotation of the NAC family in *G. arboreum* and *G. raimondii* (Shang et al., 2013, 2016) but only identified the NAC family in *G. hirsutum* by using EST scanning (Meng et al., 2009; Huang et al., 2013; Shah et al., 2013, 2014). Thus, a systematic research of the molecular evolution of the NAC family in cotton is needed. Genome-wide and comparative genomic analyses of the NAC genes in *G. arboreum, G. raimondii*, and *G. hirsutum* revealed asymmetric evolution and expansion during cotton polyploidization, as evidenced by their biased subfamily distribution, selective gene loss, unequal gene localization, and biased orthologous gene expression. This study unraveled the evolution of NAC genes in polyploid cotton and elucidated how NAC genes from the different progenitor genomes interact with each other.

## Materials and methods

### Sequence retrieval

The genome sequences of *G. arboreum, G. raimondii*, and *G. hirsutum* were downloaded from the CGP database (http://cgp.genomics.org.cn/). The genome sequences of *A. thaliana, T. cacao*, and *V. vinifera* were obtained from the Phytozome database (http://www.phytozome.net/). The Hidden Markov Model (HMM) profile of the NAC domain (PF02365) was extracted from the Pfam database (http://pfam.xfam.org/) and was used as the query by searching in the mentioned databases using the HMMER 3.0 program with the default parameters (Finn et al., 2011). Afterward, the conserved NAC domain of each putative NAC gene was confirmed by the CDD program (https://www.ncbi.nlm.nih.gov/). Finally, NAC sequences with at least four out of five conserved NAC subdomains were selected for the following research (Ooka et al., 2003).

### Chromosomal localization and structure analysis

The chromosomal localization information of the NAC genes in *G. arboreum, G. raimondii*, and *G. hirsutum*, which were respectively denoted as GaNACs, GrNACs, and GhNACs, was downloaded from the CGP database. The chromosomal distribution images of GaNACs, GrNACs, and GhNACs were illustrated by using the MapInspect software. The genomic schematic of the NAC family in cotton was visualized by GSDS 2.0 (http://gsds.cbi.pku.edu.cn). The subcellular localization was inferred using PSORT (https://psort.hgc.jp/form.html) and Cello (http://cello.life.nctu.edu.tw/). The orthologous groups (OG) of NAC proteins in cotton were identified through OrthoMCL clustering (http://orthomcl.org/orthomcl/).

### Phylogenetic analysis

The NAC genes in this study were aligned with the ClustalX program. Then, neighbor-joining (NJ) phylogenetic trees were constructed in PHYLIP and MEGA with 1,000 bootstrap replicas (Plotree and Plotgram, 1989; Kumar et al., 2016). Meanwhile, FastTree version 2.1.3 was used to estimate the maximum-likelihood phylogeny (Price et al., 2010). In addition, Bayesian analysis was performed in MrBayes version 3.1.2 (Ronquist and Huelsenbeck, 2003). All trees were visualized with Figtree version 1.4.0.

The conserved motifs of NAC proteins in cotton were scanned using the MEME program (http://meme-suite.org/tools/meme). Parameters were set based on a previous study (Fan et al., 2015b). Sequence logos of the conserved domains were generated with the WebLogo program (http://weblogo.berkeley.edu/).

### Gene duplication and syntenic analysis

Gene duplication events were identified on the basis of a previous report (Fan et al., 2014). A previous report identified all orthologous genes in cotton (Li et al., 2015), but these were not classified into families. The conserved synteny blocks between NAC genes in cotton were inferred using the OrthoClusterDB program (http://genome.sfu.ca/cgi-bin/orthoclusterdb/runortho.cgi). The syntenic relationships were illustrated with the Circos program. Meanwhile, the orthologous genes of GaNACs, GrNACs, and GhNACs in *A. thaliana, T. cacao*, and *V. vinifera* were searched via Blastp and phylogenetic analyses. In addition, the evolutionary rates (Ka, Ks, and Ka/Ks ratio) were estimated by KaKs_Calculator package (Zhang et al., 2006). On the basis of the synonymous substitutions per year (λ) of 2.6 × 10^−9^ for cotton, the divergent time of the duplicated NAC genes was estimated (T = Ks/2λ × 10^−6^ Mya; Zhang et al., 2015).

### Plant materials, RNA extraction, and quantitative real-time PCR

*G. raimondii, G. arboreum* (Shixiya1), and *G. hirsutum* (TM-1) were used to construct the expression patterns of the NAC genes in this study. Root, stem, and leaf samples were collected from 3-week-old seedlings. Afterward, the total RNAs of the collected samples were extracted by RNAprep pure Plant Kit (TIANDZ, China), and the first-strand cDNA was synthesized from DNase-treated RNA with PrimerScript 1st Strand cDNA synthesis kit (TaKaRa). Gene-specific primers were designed (Table S10) and then synthesized (Generay) for qRT-PCR, which was conducted in a CFX96 Realtime System (BioRad) by SYBR premix Extaq (TakaRa). qRT-PCR cycles were performed at an annealing temperature of 60°C. The endogenous control was an *EF1*α gene in all qRT-PCR analyses. Relative gene expression levels were determined using the 2^−ΔΔCt^ method. Three biological replications were performed in all reactions. The expression profiles of GaNACs, GrNACs, and GhNACs were clustered using the Cluster 3.0 software.

### Statistical analysis

The experimental data were statistically analyzed using the SAS version 8.0. All graphic presentations were performed using OrginPro 8.0 program.

## Results

### Comparative phylogenetic analysis of the NAC family in cotton

We found a total of 495 NAC genes across all three species of cotton (Table S1). *G. arboreum* and *G. raimondii* each have 142 NAC genes, whereas *G. hirsutum* has 211. Furthermore, similar methods were used to screen 106 NAC genes in *A. thaliana*, 100 NAC genes in *T. cacao*, and 68 NAC genes in *V. vinifera* (Table S2).

**Figure 1:**
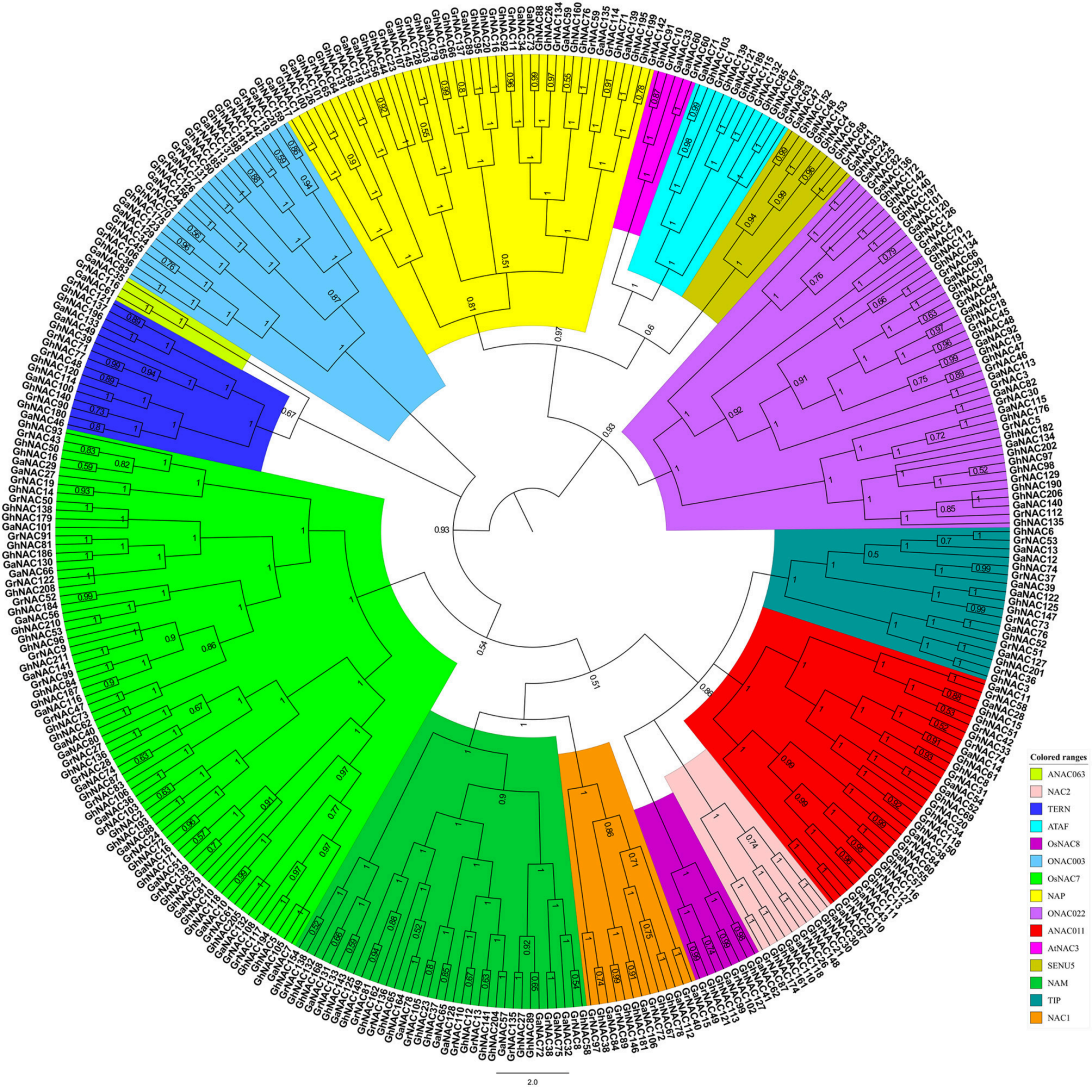
Phylogenetic tree of the NAC proteins from *G. raimondii, G. arboreum*, and *G. hirsutum*. The phylogenetic relationship was generated with the Bayesian method based on the multiple alignments of NAC protein sequences in the three *Gossypium* species. The numbers in the clades are posterior probability values. The NAC subfamilies are indicated using different colors.

**Figure 2:**
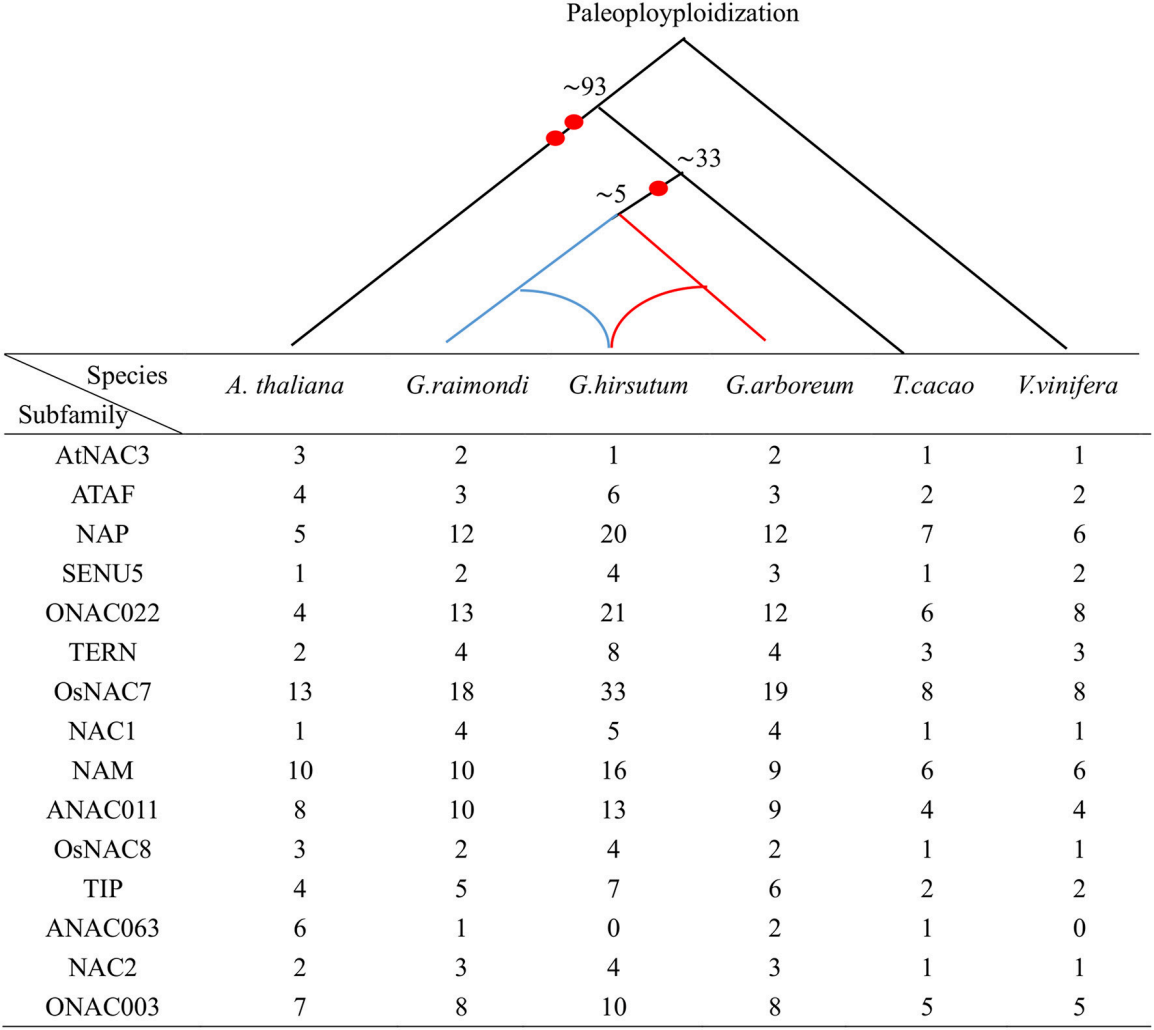
Distribution of the NAC members in three *Gossypium* species and three other genomes descended from common eudicot genome ancestors. The upper branch represents the evolutionary history of the six species. Numbers denote the predicted divergence time (MYA), and each red dot shows one whole genome duplication.

We performed the phylogenetic analysis of the identified NAC genes by using MEGA, PHYLIP, FastTree, and MrBayes (Figure 1, Figures S1–S3). The NAC subfamily was defined by a previous classification in the phylogenetic analysis (Ooka et al., 2003). In our paper, the NAC family in cotton contained 15 NAC subfamilies, each of which contained a different percentage of the genes. In each cotton species, the OsNAC7 subfamily (more than 10%) contained the most genes, followed by the ONAC022 subfamily (8–10%; Figure S4). The AtNAC3 and ANAC063 subfamilies had the least genes (<1%). Meanwhile, *G. arboreum* and *G. raimondii* contained a similar number of NAC genes in each subfamily (Figure 2). Almost every subfamily had more NAC genes in *G. arboreum* and *G. raimondii* than in *A. thaliana, T. cacao*, and *V. vinifera*. In addition, *G. hirsutum* in the AtNAC3, ANAC011, TIP, and ONAC003 subfamilies displayed a similar gene number to *G. arboreum* and *G. raimondii*. No GhNAC was identified in the ANAC063 subfamily. Meanwhile, the GhNAC number in other subfamilies was almost twice the GaNAC and GrNAC numbers. Furthermore, GhNAC loss in the AtNAC3 and ANAC011 subfamilies was mainly due to the AA-derived NACs, whereas the ONAC003 subfamily primarily lost many GhNACs from the DD-derived NACs. The TIP subfamily lost GhNACs from both AA-derived and DD-derived NACs (Table S7).

The orthologous group (OGs) is identified as orthologs by OrthoMCL. With the use of OrthoMCL clustering, 44 OGs were identified in cotton (Table S3). Each subfamily shared one or more distinct OGs, and different subfamily contains different OGs. The OG distributions of the NAC family in cotton agreed with the phylogenetic analysis.

### Structural analysis and expression patterns of the NAC family in cotton

**Figure 3:**
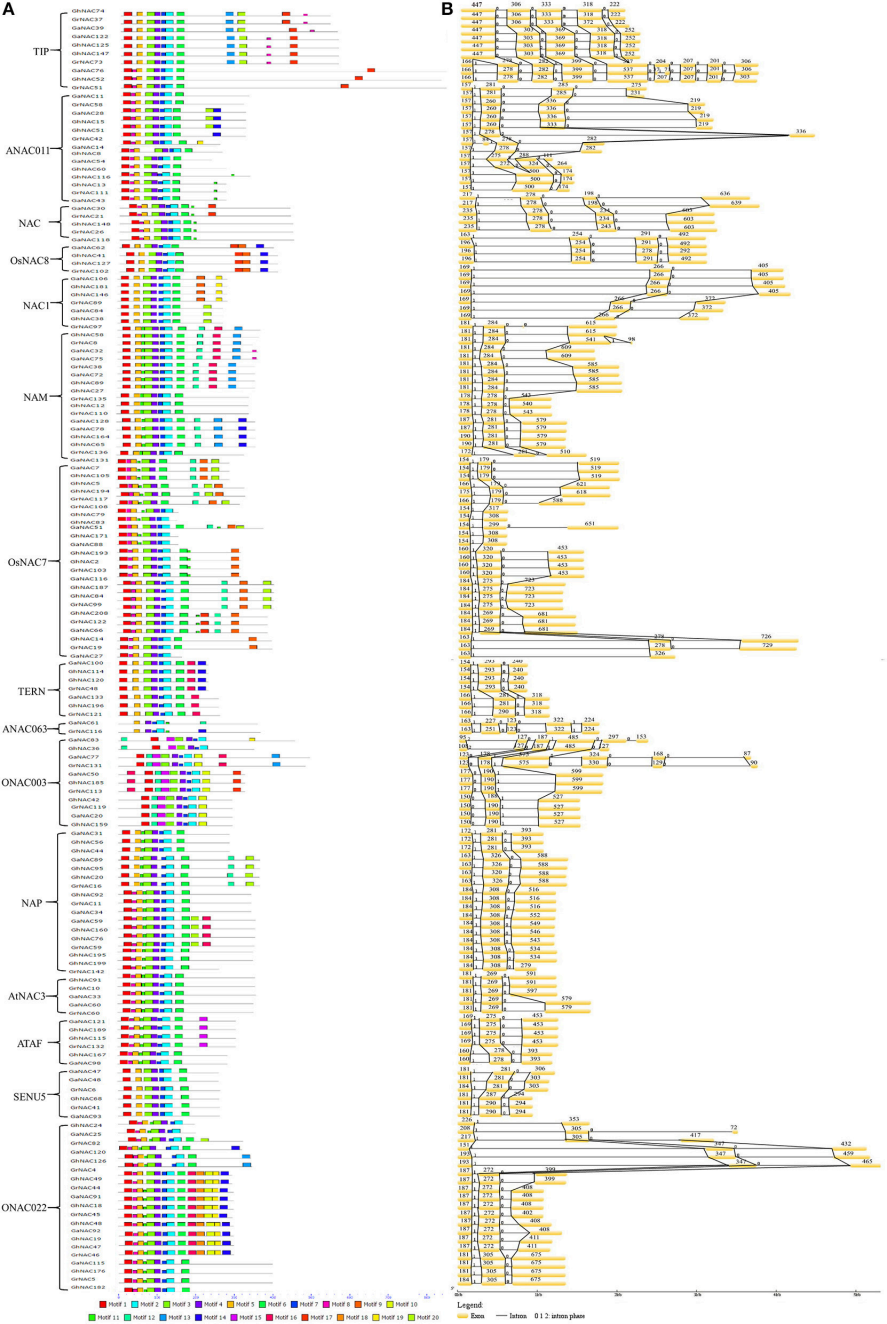
Putative conserved domain distribution and gene structure dynamics of some NAC members in *G. raimondii, G. arboreum*, and *G. hirsutum*. **(A)** The conserved motifs were identified by MEME. Different motifs were represented by various colored boxes. The location of each motif can be estimated using the scale at the bottom. Subfamily designations are indicated by brackets. **(B)** Gene structures of the NAC members were performed by the GSDS software.

The MEME program revealed 20 distinct conserved motifs in the NAC family (Figure 3A). On the basis of the distribution of the conserved motifs, all of the NAC genes in cotton can be classified into 15 subfamilies, which is consistent with the categorization from the phylogenetic analysis (Figure 1). Motifs 1, 2, 3, and 4 were shared in the NAC family, and they corresponded to highly conserved subdomains A, C, and D (Figure S5). Motifs 5 and 6 corresponding to subdomains B and E also existed in most NAC genes. Moreover, some NAC-subfamily-specific motifs were identified in some NAC subfamilies. Meanwhile, a DNA-binding domain (DBD) existed in subdomain C, and a nuclear localization signal (NLS) was found in subdomain D.

Gene structure and intron phase were investigated in the NAC family (Figure 3B, Table S4). The main gene structure was three exons and two introns. The length of the first and second exons was conserved (150–230 and 180–320 bp, respectively). However, the length of the third exon was highly variable, especially for ANAC0111, ONAC003, and TIP subfamilies. In addition, several subfamilies showed differences in gene structure due to gains and losses of introns. Some TIP genes lacked an intron between the first and second exons, and the ANAC011 subfamily showed the similar loss between the second and third exons. A gain of an intron in the third exon was seen in the ONAC003, ANAC011, and TIP subfamilies. By contrast, no change was observed in the gene structure of the NAC1, AtNAC3, and SENU5 subfamilies.

**Figure 4:**
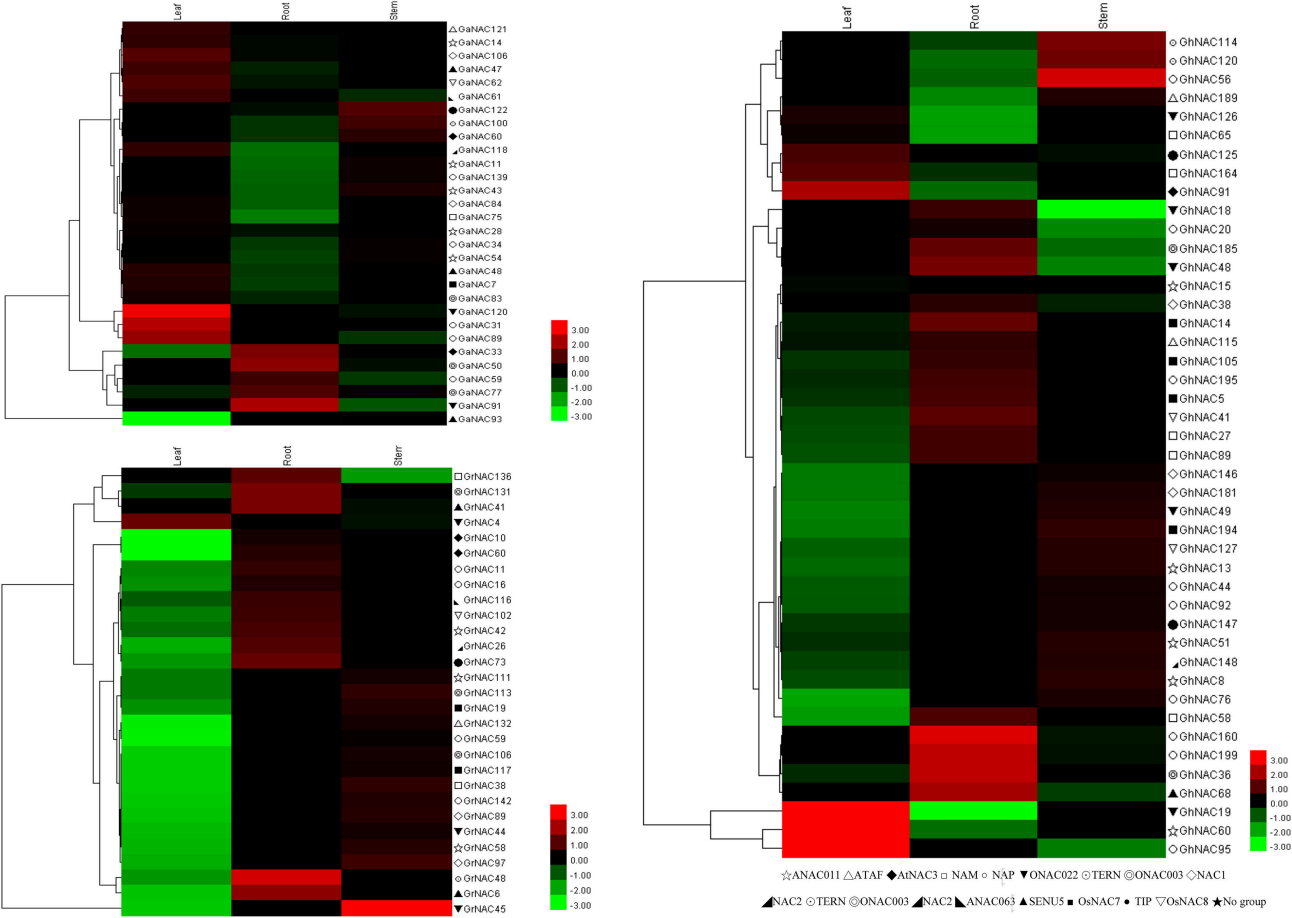
Heat map representation and hierarchical clustering of GaNACs, GrNACs, and GhNACs across different tissues. The color bar represents the relative signal intensity value.

PSORT and Cello analyses showed that most of the NAC genes in cotton are localized to the nucleus (Table S5). qRT-PCR analysis of some randomly selected GhNACs, GaNACs, and GrNACs in the roots, stems, and leaves showed that the expression patterns of these genes significantly differed in different tissues (Figure 4). In general, most of the NACs in the NAP subfamily were highly expressed in the roots, whereas those in the ANAC011 subfamily were predominantly expressed in the stems. Thus, the NAC genes from the same subfamily exhibited similar expression profiles in cotton.

### Genomic locations and duplication of the NAC genes in cotton

Chromosomal location images of the NAC family were generated in three *Gossypium* species (Figures S6, S7, Table S6). Each chromosome contained NAC genes, but these NAC genes are distributed unevenly across the cotton chromosomes (Figures S4, S6, S7). In *G. arboreum*, AA13 had the largest number of GaNACs. In *G. raimondii*, DD01, DD07, and DD08 contained the largest number of GrNACs. In *G. hirsutum*, At09 and Dt05 contained the maximum number of GhNACs in the At and Dt subgenomes, respectively. Moreover, many NAC genes were clustered within a short distance, such as the middle of AA13 and At09 and the top of DD08 and Dt07.

**Figure 5:**
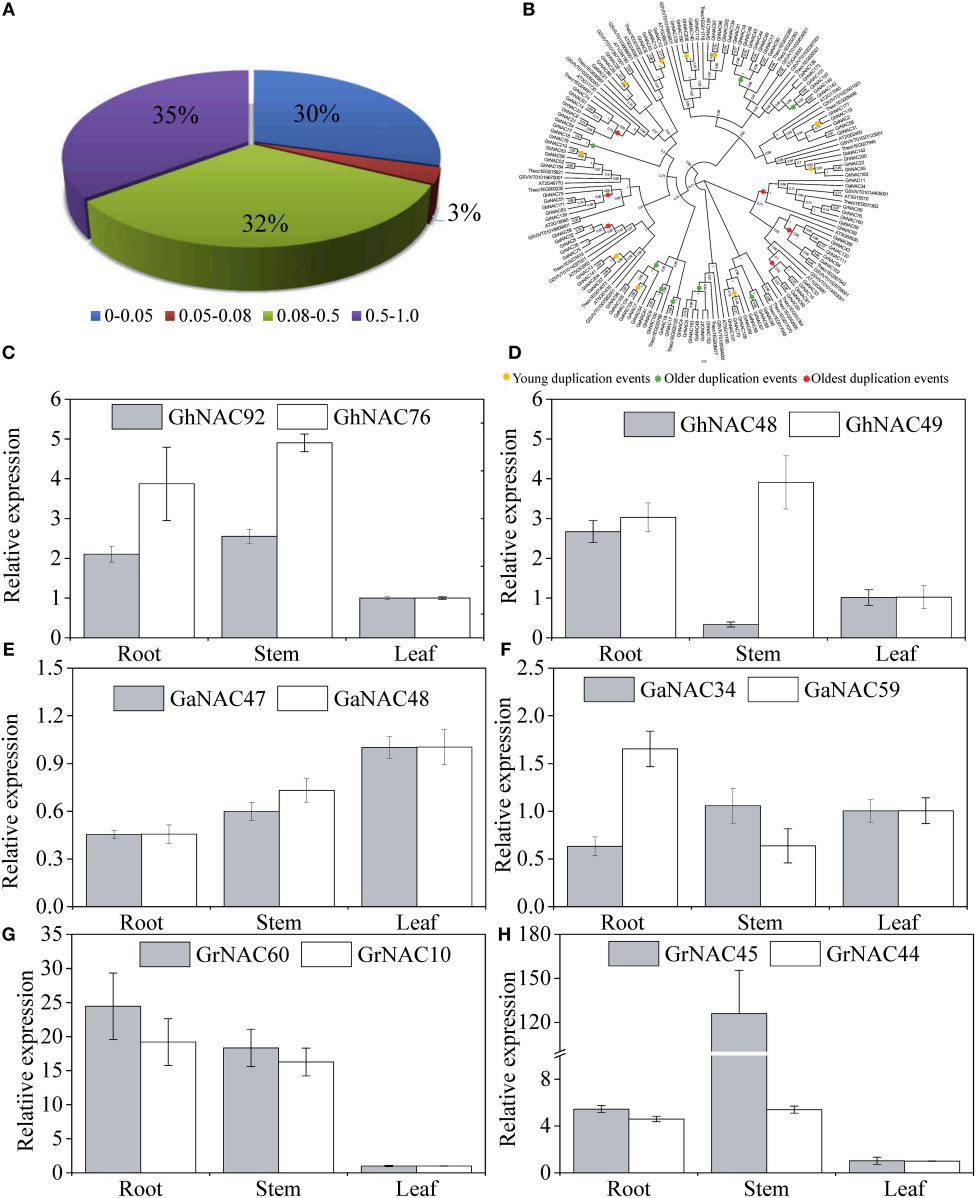
Age distribution, phylogenetic relationship, and expression analysis of the duplicated NAC genes in *G. raimondii, G. arboretum*, and *G. hirsutum*. **(A)** Age distribution of the duplicated GhNACs, GaNACs, and GrNACs based on Ks values. **(B)** Phylogenetic relationship among the duplicated GhNACs, GaNACs, and GrNACs and their orthologous genes in three other dicots. The phylogenetic tree was constructed by using the FastTree method. The numbers in the clades are the FastTree bootstrap values. The circles highlight nodes representing the duplication events. **(C–H)** Expression pattern of some duplicated GhNACs, GaNACs, and GrNACs in different tissues.

Gene duplication events were identified to illustrate the NAC expansion in cotton. In this study, 12, 8, and 14 duplicated gene pairs were found in *G. raimondii, G. arboreum*, and *G. hirsutum*, respectively (Table 1). These gene pairs belonged to the ONAC022, NAM, and NAP subfamilies. According to the sequence analysis and the chromosomal location, 13 gene pairs were related to tandem duplication events, whereas 21 gene pairs were involved in segmental duplication events. Moreover, the expression relationships of some duplicated genes were investigated (Figures 5C–H). Three pairs of genes (GhNAC 92/76, GaNAC 47/48, and GrNAC 10/60) had highly similar expression levels, whereas others (GhNAC 48/49, GaNAC 34/59, and GrNAC 44/45) showed limited expression divergence. For example, GhNAC 48/49 exhibited a similar transcript level in the roots and leaves but not in the stems. Furthermore, Ka/Ks ratios were less than 1 in all duplicated gene pairs (Table 1). The Ks values were generally between 0.08 and 1.0 (67%), and the Ks values of 10 duplicated gene pairs were less than 0.05 (Figure 5A). Specifically, most of Ks values were between 0.08 and 1.0 in *G. raimondii* (84%) and *G. arboretum* (75%), while half of Ks values was less than 0.05 in *G. hirsutum* (Figure S8).

**Table 1:** Ka/Ks analysis and estimated divergence time for the NAC duplicated genes in *G.arboreum, G.raimondii*, and *G.hirsutum*.

**Species**	**Duplicated gene 1**	**Duplicated gene 2**	**Subfamily**	**Ka**	**Ks**	**Ka/Ks**	**Purifying selection**	**Duplicate type**	**Age(MYA)**
*G. arboreum*	GaNAC102	GaNAC69	No group	0.064117	0.656974	0.097594	Yes	Segmental	126.3412
	GaNAC104	GaNAC124	No group	0.010065	0.032472	0.309972	Yes	Segmental	6.2445
	GaNAC12	GaNAC13	TIP	0.008645	0.011390	0.759003	Yes	Tandem	2.1903
	GaNAC33	GaNAC60	AtNAC3	0.080250	0.663169	0.121010	Yes	Segmental	127.5325
	GaNAC34	GaNAC59	NAP	0.074491	0.685007	0.108744	Yes	Segmental	131.7321
	GaNAC47	GaNAC48	SENU5	0.031741	0.140173	0.226438	Yes	Tandem	26.9563
	GaNAC75	GaNAC32	NAM	0.088431	0.742200	0.119148	Yes	Segmental	142.7308
	GaNAC90	GaNAC91	ONAC022	0.083246	0.228827	0.363795	Yes	Tandem	44.0052
*G. raimondii*	GrNAC10	GrNAC60	AtNAC3	0.089186	0.633726	0.140732	Yes	Segmental	121.8704
	GrNAC101	GrNAC140	ONAC022	0.060670	0.157019	0.386389	Yes	Segmental	30.1960
	GrNAC11	GrNAC59	NAP	0.076075	0.652593	0.116573	Yes	Segmental	125.4987
	GrNAC130	GrNAC94	No group	0.186174	0.858892	0.216761	Yes	Segmental	165.1715
	GrNAC17	GrNAC18	No group	0.119789	0.354589	0.337825	Yes	Tandem	68.1902
	GrNAC31	GrNAC74	ANAC011	0.094354	0.714574	0.132042	Yes	Segmental	137.4181
	GrNAC38	GrNAC8	NAM	0.094469	0.631973	0.149482	Yes	Segmental	121.5333
	GrNAC44	GrNAC45	ONAC022	0.079115	0.253014	0.312692	Yes	Tandem	48.6565
	GrNAC54	GrNAC96	No group	0.136243	0.224140	0.607851	Yes	Segmental	43.1038
*G. raimondii*	GrNAC67	GrNAC68	No group	0.020244	0.067305	0.300773	Yes	Tandem	12.9433
	GrNAC70	GrNAC69	No group	0.009034	0.037721	0.239489	Yes	Tandem	7.2541
	GrNAC77	GrNAC78	No group	0.091657	0.149849	0.611666	Yes	Tandem	28.8171
*G. hirsutum*	GhNAC153	GhNAC152	SENU5	0.031961	0.130171	0.245532	Yes	Tandem	25.0329
	GhNAC17	GhNAC18	ONAC022	0.083637	0.245527	0.340641	Yes	Tandem	47.2167
	GhNAC177	GhNAC119	No group	0.000000	0.000002	0.000001	Yes	Segmental	0.0004
	GhNAC183	GhNAC200	No group	0.000000	0.005089	0.000001	Yes	Tandem	0.9786
	GhNAC190	GhNAC206	ONAC022	0.000000	0.000002	0.000001	Yes	Segmental	0.0003
	GhNAC197	GhNAC142	ONAC022	0.564578	0.708338	0.797047	Yes	Segmental	136.2188
	GhNAC204	GhNAC141	NAM	0.008889	0.011327	0.784748	Yes	Segmental	2.1782
	GhNAC28	GhNAC29	No group	0.002171	0.021142	0.102669	Yes	Tandem	4.0657
	GhNAC49	GhNAC48	ONAC022	0.082394	0.272132	0.302772	Yes	Tandem	52.3331
	GhNAC53	GhNAC210	OsNAC7	0.004257	0.009447	0.450641	Yes	Segmental	1.8168
	GhNAC76	GhNAC92	NAP	0.085041	0.665845	0.127719	Yes	Segmental	128.0471
	GhNAC79	GhNAC171	OsNAC7	0.037436	0.637425	0.058730	Yes	Segmental	122.5817
	GhNAC9	GhNAC100	No group	0.107982	0.161159	0.670033	Yes	Segmental	30.9921
	GhNAC97	GhNAC98	ONAC022	0.002492	0.028542	0.087322	Yes	Tandem	5.4888

Orthologous gene pairs of duplicated NACs were identified in *G. arboreum, G. raimondii*, and *G. hirsutum* (Table S7). Nine duplicated groups were found in the NAC family and can be classified into two types. The first type of duplication events occurred in the diploid species (*G. arboretum* and *G. raimondii*), and the second type existed in the *G. hirsutum* and its diploid ancestor. The first type contained four duplicated groups (GaNAC 102/69 and GrNAC 130/94, GaNAC75/32 and GrNAC 38/8, GaNAC34/59 and GrNAC 11/59, and GaNAC 33/60 and GrNAC 10/60), and their corresponding Ks values were between 0.5 and 1.0. The second type of duplication events had similar duplication ages (GaNAC 90/91 and GhNAC 17/18, GaNAC 47/48 and GhNAC 152/153, GrNAC 101/140 and GhNAC 197/142, GrNAC 11/59 and GhNAC 76/92, and GrNAC 44/45 and GhNAC 48/49), and all of the Ks values was more than 0.08. The five duplicated groups originated from the SENU5, ONAC022, and NAP subfamilies.

Meanwhile, the orthologs of 34 duplicated gene pairs were detected in *A. thaliana, T. cacao*, and *V. vinifera* (Table S8). The relative time of the duplication events, including those of the orthologous genes, was predicted through phylogenetic tree analysis (Figure 5B). The young duplication events mainly occurred in *G. hirsutum*, with Ks values < 0.05. The oldest duplication events mainly existed in diploid cotton, with Ks values more than 0.5. Furthermore, all of the oldest duplicated gene pairs was involved in segmental duplication events, while most of the older duplicated gene pairs (75%) was related to tandem duplication events. The young duplication events contained the similar proportion of segmental and tandem duplication events.

### Gene loss and orthologous exchange during cotton NAC evolution

**Figure 6:**
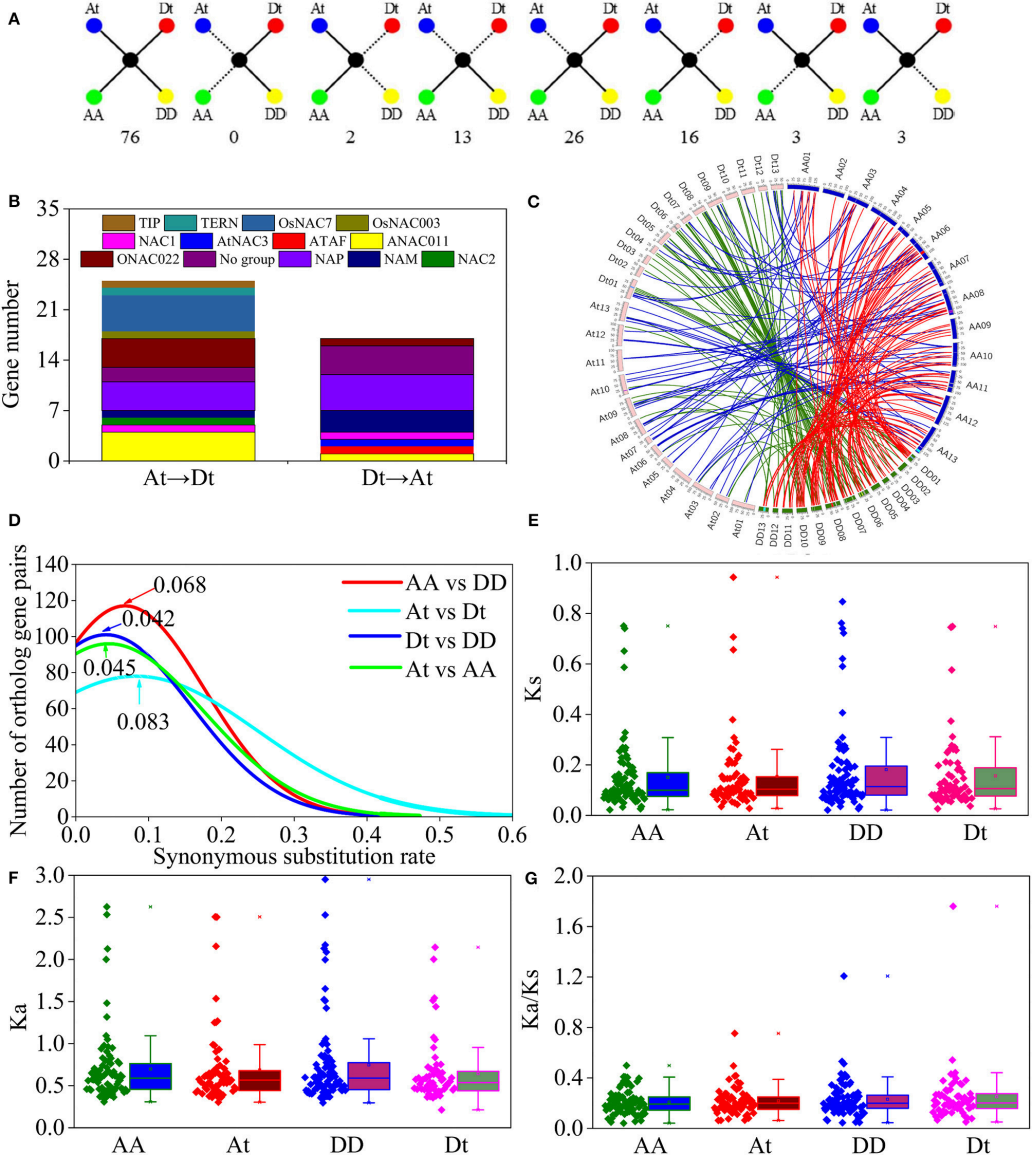
Syntenic analysis and evolution of the NAC members in *G. hirsutum* (At and Dt), and in two diploid cotton genomes, *G. arboretum* (AA) and *G. raimondii* (DD). **(A)** Scenarios and number of gene conservation. Solid lines show currently observed genes, and dotted lines show lost genes. The numbers beneath each drawing represent the number of gene pairs found in the cotton genomes. **(B)** Statistics of NAC members in *G. hirsutum* transferred from Dt to At and from At to Dt. **(C)** Circos diagrams of the NAC homologous gene pairs in *G. raimondii, G. arboreum*, and *G. hirsutum*. The homologous genes in *G. raimondii* and *G. arboretum, G. raimondii* and *G.hirsutum*, and *G. arboreum* and *G. hirsutum* are linked with red, green, and blue lines, respectively. The colored blocks within the chromosomes represent the synteny blocks in cotton NAC members. **(D)** Distribution of Ks values for the NAC orthologous gene sets among *G. raimondii, G. arboreum*, and *G. hirsutum*. Peak values for each comparison are indicated with arrows. **(E)** Distribution of Ks values between four cotton genomes and *T. cacao*. **(F)** Distribution of Ka values between four cotton genomes and *T. cacao*. **(G)** Distribution of Ka/Ks values between four cotton genomes and *T. cacao*.

In this study, 152 orthologous gene pairs of the NAC family were obtained in cotton (Table S7). The At and Dt subgenomes of *G. hirsutum* and their corresponding ancestral genome contained 97 and 105 gene pairs, respectively. A total of 76 of 131 pairs in both *G. raimondii* and *G. arboreum* were conserved in *G. hirsutum* (Figure 6A). Meanwhile, two pairs were absent from the DD genome and the Dt subgenome, whereas no gene pairs were lost in the AA genome and the At subgenome. Thirteen pairs in both *G. raimondii* and *G. arboreum* did not obtain their orthologous genes in *G. hirsutum*. A total of 26 and 16 genes were lost in the At and Dt subgenomes, respectively, and three genes were lost in the AA and DD genomes. Furthermore, gene loss was distributed unevenly in the NAC subfamily. For example, 10 genes were lost in the OsNAC7 subfamily, and 7 genes were absent in the ANAC011 subfamily. However, no gene loss occurred in the ATAF, OsNAC8, and TERN subfamilies.

Considering the genomic localization, we found that 42 NAC genes in *G. hirsutum* exhibited orthologous exchange (Figure 6B). Results showed that 25 GhNACs were transferred from Dt to At, and 17 GhNACs were transferred from At to Dt. In addition, 7, 11, and 13 synetic blocks in the NAC family were predicted between *G. hirsutum* and *G. arboretum, G. hirsutum* and *G. raimondii*, and *G. raimondii* and *G. arboretum*, respectively (Figure 6C, Table S9). One synetic block was transferred from Dt to At, whereas two synetic blocks were transferred from At to Dt. Nine GhNACs in the NAP subfamily were associated with the orthologous exchange, whereas no transformation occurred in the ANAC063, OsNAC8, and SENU5 subfamilies.

### Asymmetric evolution of the At and Dt subgenomes in the NAC family

Comparison of the Ks values of the NAC orthologous gene sets revealed Ks value peaks at 0.068 and 0.083 between AA and DD and between At and Dt, respectively, and their corresponding divergent times were 13.1 and 16.0 MYA (Figure 6D). Meanwhile, the divergent times between At and AA and between Dt and DD were 8.7 and 8.1 MYA, respectively (Ks peaks at 0.045 and 0.042). In addition, both Ka and Ks values declined in the Dt subgenome, compared with their corresponding DD genome (Figures 6E,F). However, Ka and Ks values were similar between the At subgenome and the AA genome. Moreover, Ka/Ks ratio slightly elevated in the At and Dt subgenomes relative to their corresponding progenitor genomes. Ka/Ks ratios were higher in the Dt subgenome and the DD genome than in the At subgenome and the AA genome (Figure 6G).

### Expression of the orthologous genes in cotton

**Figure 7:**
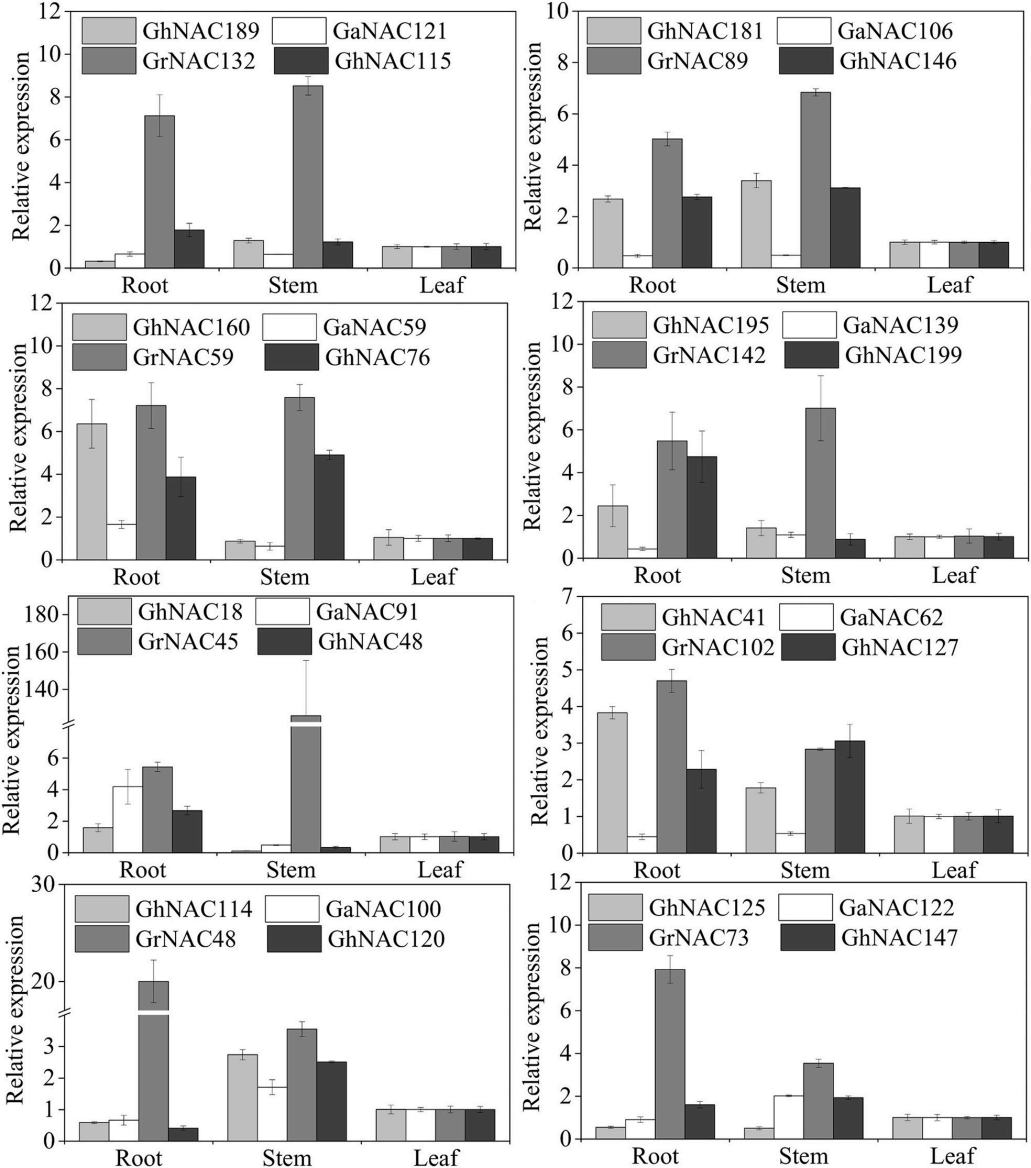
Expression analysis of the orthologous gene pairs from *G. arboreum, G. raimondii*, and *G. hirsutum* in different tissues.

The expression patterns of some GhNAC genes and their orthologous GaNAC and GrNAC genes were analyzed in different tissues (Figure 7). Most of the gene pairs showed a similar expression pattern, except for GhNAC181/GaNAC106, GhNAC41/GaNAC62, GhNAC48/GrNAC45, GhNAC115/GrNAC132, and GhNAC120/GrNAC48. Most of GhNACs were more highly expressed than the orthologous GaNACs in the At subgenome, whereas most of the GhNACs and their its orthologous GrNACs showed the opposite expression in the Dt subgenome. Moreover, most NAC genes in the DD genome or the Dt subgenome had higher transcript levels than their orthologous genes in the AA genome or the At subgenome.

## Discussion

With cotton being an important crop, sequencing of its genome facilitates our understanding of eudicot and polyploid plant evolution. The present research analyzed the NAC expansion and evolutionary history from the diploid to the allotetraploid cotton.

In this study, we identified 142 GaNACs, 142 GrNACs, and 211 GhNACs in cotton species (Table S1). The number of NAC genes was larger in each *Gossypium* than in three other eudicots (Table S2). This finding may be due to the extra whole genome duplication in the *Gossypium* lineage (Paterson et al., 2012; Li et al., 2015; Zhang et al., 2015). Although the A-related genome is larger than the D-related genome, the number of NAC members is similar in the two genomes. The reason may be related to more transposable elements in the A-related genome (Li et al., 2014, 2015). Through phylogenetic analysis, 15 NAC subfamilies were clustered in cotton (Figure 1, Figures S1–S3). The OsNAC7 and ONAC022 subfamilies had the most genes, whereas the AtNAC3 and ANAC063 subfamilies had the least NACs (Figure S4). Similar distribution of the NAC family existed in maize (Fan et al., 2014) and banana (Shan et al., 2012). The distribution of OG, gene structure, and conserved motif confirmed the similar classification (Figure 3, Tables S3, S4). Meanwhile, *G. hirsutum* contains almost twice as many genes in most NAC subfamilies as its diploid ancestors, with the exception of the AtNAC3, ANAC011, TIP, ONA003, and ANAC063 subfamilies. This indicates that *G. hirsutum* might have experienced gene loss in those latter subfamilies (Figure 2). This phenomenon may be related to the diploidization process after polyploidization (Otto, 2007; Soltis and Soltis, 2009). After polyploidization, the ployploid genome may reshuffle extensively to regain the diploid heredity (Wang et al., 2016). Moreover, the NAC family in cotton has a typical NAC structure (Figure 3A, Figure S5). The lengths of the first and second exons in the gene structure were relatively conserved partially because of the conserved encoding of the NAC domain (Figure 3B). However, exon length and intron insertions were different in the third exon, especially for the ANAC011, ONAC003, and TIP subfamilies. The gain and loss of introns might have resulted in the different structures of NAC genes in cotton. Besides, the putative NLS existed in subdomain D. Most of the NAC genes in cotton were predicted to be nuclear proteins through PSORT and Cello analyses (Table S5). This has been confirmed for some GhNACs through subcellular localization (Fan et al., 2015a; He et al., 2016). In addition, tissue-specific expression patterns were relatively conserved in the NAC subfamily. Different NAC subfamilies may have different biological functions, mainly due to the distinct structures, especially in the TAR region (Figure 4, Figure S5).

The expansion of NAC genes was investigated in cotton. We found 34 duplicated NAC gene pairs in cotton (Table 1). The duplication events had strong expansion preference for some NAC subfamilies, including ONAC022, NAM, and NAP. Thus, the duplication event is not random across NAC subfamilies during the cotton evolution. The retention may be related to the genomic fractionation in cotton (Wang et al., 2016). Meanwhile, orthologous genes of the duplicated NACs were isolated in five other eudicots (Table S8). Due to their similar duplication ages, some duplicated GhNACs in the SENU5, ONAC022, and NAP subfamilies might have originated via duplications in their diploid ancestors. However, we could not find their corresponding duplicated GhNACs in other duplicated GaNACs or GrNACs. Thus, the NAC subfamily might have various retention rates during whole genome reshuffling after interspecific hybridization. On the basis of the chromosomal location, we found that the main duplication events is different during the cotton evolutionary history (Figures S6, S7, Table 1). The segmental duplication of NAC genes dominated the expansion in the paleohexaploidization event and might be related to the paleopolyploidy (Li et al., 2015; Zhang et al., 2015). Most of the duplicated genes after the cotton-specific decaploidy resulted from tandem duplication events, and might result from the chromosomal breakages and rearrangements after polyploidization (Buggs et al., 2012). However, the segmental and tandem duplication events were highly prevalent after interspecific hybridization, and might be different from previously reported polyploidy-related duplication events. Moreover, the Ka/Ks ratios were <1 in all duplicated gene pairs, indicating that the NAC genes in cotton have mainly experienced purifying selection (Table 1). The expression profiles of some duplicated genes revealed similar results (Figures 5C–H). These results indicate that most of the duplicated genes of the NAC family might have retained some essential functions during sequent evolution.

Cotton experienced two major events as evidenced by the Ks distribution (Figure 6D). Two Ks peaks were between 0.045 and 0.042 and between 0.068 and 0.083, and their corresponding times may correspond to the interspecific hybridization and the divergence between their diploid ancestors. Our Ks values are a little higher than the previous reports (Li et al., 2015; Zhang et al., 2015). The main reason may be fewer orthologous gene sets in our study. Then, cotton has undergone two major whole genome duplication events: the paleohexaploidization event and the cotton-specific decaploidy (Paterson et al., 2012; Li et al., 2015; Zhang et al., 2015). In our study, 67% of the duplication events might have occurred in the aforementioned periods, expecially in *G. raimondii* and *G. arboretum* (Figure 5A, Figure S8). The whole duplication events resulted in the decaploid ancestor in cotton (Wang et al., 2016). Half of the duplication events in *G. hirsutum* might have occurred after interspecific hybridization. The results suggest that *G. hirsutum* might have experienced a complex and distinct evolutionary history from its diploid ancestors. Phylogenetic analysis also indicated that the duplication events of GaNACs and GrNACs might have mainly occurred in the paleohexaploidization event and the cotton-specific decaploid and that most of the duplicated GhNACs might have occurred after interspecific hybridization (Figure 5B).

*G. hirsutum* originated from the interspecific hybridization between AA-genome and DD-genome species (Li et al., 2015). In the present study, orthologous genes of GhNACs were identified in the diploid ancestors (Figure 6C, Table S7). Compared with NAC loss in the different genomes, the loss rate of NAC genes was higher in *G. hirsutum* than in diploid species. During the polyploidization, many chromosomal breakages and rearrangements led to gene loss and gene retentions (Buggs et al., 2012). The high loss rate may exist in other allopolyploid *Gossypium* species (Liu et al., 2015). In addition, more NACs were lost in the At subgenome than in the Dt subgenome during the formation of *G. hirsutum*, which led to more orthologous gene pairs and synetic blocks of the NAC family in the Dt subgenome and the DD genome (Figure 6C, Table S9). In the Dt subgenome, multilocus interactions are largely preponderant, which highlights the complex of NAC genes in cotton (Waghmare et al., 2016). Moreover, due to the different functions of the At and Dt subgenomes, the NAC family in *G. hirsutum* may be primarily associated with stress tolerance (Zhang et al., 2015). In previous studies, some GhNAC genes exhibited upregulated expression under stress (Meng et al., 2009; Shah et al., 2013, 2014; He et al., 2016). Thus, NAC genes in cotton have different loss rates in different genomes.

Orthologous exchange was identified in the At and Dt subgenomes (Figure 6B). A large number of GhNACs were transferred from Dt to At, which may increase the expression of NACs in the current At subgenome relative to the ancestral AA genome. Similar results were found in the *Adh* locus (Small and Wendel, 2002). The orthologous exchange of NAC genes might distinguish *G. hirsutum* from its ancestor species in important phenotypic changes, including plant morphology and economic traits. This exchange is also confirmed by the genetic linkage map (Li et al., 2015). Moreover, gene loss and orthologous exchange showed NAC subfamily preference, and different NAC subfamilies exhibited different rates of gene loss and orthologous exchange. Furthermore, Ka and Ks analyses of the NAC genes showed that the Dt subgenome might have evolved slower than the DD genome while the At subgenome and the AA genome might have had similar evolutionary rates (Figures 6E,F). Analysis of Ka/Ks ratio revealed that NAC genes might have experienced less positive selection in the At subgenome than in the Dt subgenome (Figure 6G). This result may partly explain why most At or Dt subgenome shows the highest orthologous with the corresponding diploid AA homologous chromosomes (Li et al., 2015) Thus, the At and Dt subgenomes had undergone asymmetric evolution after interspecific hybridization, which might be caused by different levels of selection pressure and introgressed chromatin (Waghmare et al., 2016). In addition, GhNAC redundancy created by allotetraploidy might have allowed relatively relaxed purifying selection in both At and Dt subgenomes. In the allotetraploid cotton, different genome-derived NACs possibly interacted with each other. Furthermore, most of the orthologous gene pairs between GhNAC and its orthologous GaNACs or GrNACs have the similar expression model in different tissues, which indicated limited functional divergence after interspecific hybridization (Figure 7). However, most of the NAC genes exhibited biased D-ortholog expression, which might have led to subfunctionalization of the NAC genes. This result suggests that the NAC genes in the DD genome or the Dt subgenome play important roles in plant development. The asymmetric expression was also observed in the LTP and miRNA families (Xie and Zhang, 2015; Li, F. et al., 2016; Li, X. et al., 2016).

## Conclusion

In this report, we isolated 142 GaNACs, 142 GrNACs, and 211 GhNACs in cotton. The main duplication event of NAC genes was different during the cotton evolutionary history. The duplication events mainly occurred in the paleohexaploidization event (35%) and the cotton-specific decaploidy (32%). Moreover, some duplicated GhNACs might have originated from their diploid ancestor, and another might have occurred after interspecific hybridization. Meanwhile, 15 NAC subfamilies in cotton were clustered with NAC subfamily preference. In addition, NAC genes in the At and Dt subgenomes have different gene loss rates, orthologous exchange, evolutionary rates, and expression levels. Taken together, the findings of the present study could broaden our understanding on the molecular evolution and expansion history of the NAC family in cotton during polyploidization.

## Author contributions

KF and WenL designed the research. KF, FL, JC, and ZL performed the experiments. KF, FL, JC, WeiL, and SC analyzed the data. KF, JL, and WenL wrote the paper with contributions from all the authors.

### Conflict of interest statement

The authors declare that the research was conducted in the absence of any commercial or financial relationships that could be construed as a potential conflict of interest.

## Supplementary material

Figure S1 Evolutionary analysis of the NAC family in *G. raimondii, G. arboreum, G. hirsutum, A. thaliana, T. cacao*, and *V. vinifera* with the MEGA program. Amino acid sequences were aligned with ClustalW, and the neighbor-joining (NJ) tree was generated through the MEGA program. The numbers in the clades are the bootstrap values. The subfamilies within the NAC family are grouped by colors.

Figure S2 Phylogenetic relationship of the NAC proteins from *G. raimondii, G. arboreum*, and *G. hirsutum*. Amino acid sequences were aligned with ClustalW, and the neighbor-joining (NJ) tree was generated through PHYLIP package. The numbers in the clades are the bootstrap values. The subfamilies within the NAC family are grouped by colors.

Figure S3 Phylogenetic analysis of GaNACs, GrNACs, and GhNACs. The phylogenetic tree was constructed with the FastTree method. The tree is unrooted. The numbers in the clades are the FastTree bootstrap values. Each NAC subfamily is indicated using a specific color.

Figure S4 Percentage of NAC subfamily **(A,C,E)** and its chromosomes localizations **(B,D,F)** in *G. arboretum* **(A,B)**, *G. raimondii* **(C,D)**, and *G. hirsutum* **(E,F)**.

Figure S5 Conserved domain analysis of the NAC proteins in *G. arboretum, G. raimondii*, and *G. hirsutum*. Sequence logos of the NAC domain **(A)** and TAR region **(B)** were shown with the WebLogo program. The height of letter designating the amino acid residue at each position represents the degree of conservation. The x-axis represents the sequence positions in the corresponding conservative domains, and the y-axis represents the information content measured in bits.

Figure S6 Chromosomal locations of GaNAC and GrNAC on all 13 chromosomes. The scale used is megabases (Mb). Markers before the gene names indicate the NAC subfamily. The red lines mark the tandem duplication, and the genes related to segmental duplication are joined by gray lines.

Figure S7 Chromosomal locations of GhNAC on the 26 chromosomes. The scale used is megabases (Mb). Markers before the gene names indicate the NAC subfamily. The red lines mark the tandem duplication, and the genes related to segmental duplication are joined by gray lines.

Figure S8 Age distribution of the duplicated GaNACs, GrNACs, and GhNACs based on Ks values.

Table S1 NAC proteins identified in *G.raimondii, G.arboreum*, and *G.hirsutum*.

Table S2 NAC proteins identified in *A. thaliana, T. cacao*, and *V. vinifera*.

Table S3 Orthologous groups of GaNAC, GrNAC, and GhNAC found through OtrhoMCL clustering.

Table S4 Structural analysis of GrNAC, GaNAC, and GhNAC identified in this study.

Table S5 Subcellular localization of NAC proteins in *G.raimondii, G.arboreum*, and *G.hirsutum* by using PSORT and Cello software.

Table S6 Genomic locations of NAC genes in *G.raimondii, G.arboreum*, and *G.hirsutum*.

Table S7 Orthologous gene pairs of NAC proteins in *G.hirsutum, G.arboreum*, and *G.raimondii*.

Table S8 Duplicated GaNAC, GrNAC, and GhNAC and their corresponding orthologous genes in three other dicots.

Table S9 Information of synetic blocks predicted among *G.hirsutum, G. arboretum*, and *G.raimondii* NAC homologs.

Table S10 PCR primers used in this study.
