# Supplementary material for: Asymmetric Evolution and Expansion of the NAC Transcription Factor in Polyploidized Cotton
Source: Front Plant Sci. 2018 Jan 30;9:47. doi: 10.3389/fpls.2018.00047 (PMC5797638; doi:10.3389/fpls.2018.00047)
Supplement: Figure S2 — Phylogenetic relationship of the NAC proteins from G. raimondii, G. arboreum, and G. hirsutum. Amino acid sequences were aligned with ClustalW, and the neighbor-joining (NJ) tree was generated through PHYLIP package. The numbers in the clades are the bootstrap values. The subfamilies within the NAC family are grouped by colors. [file Image2.PDF]

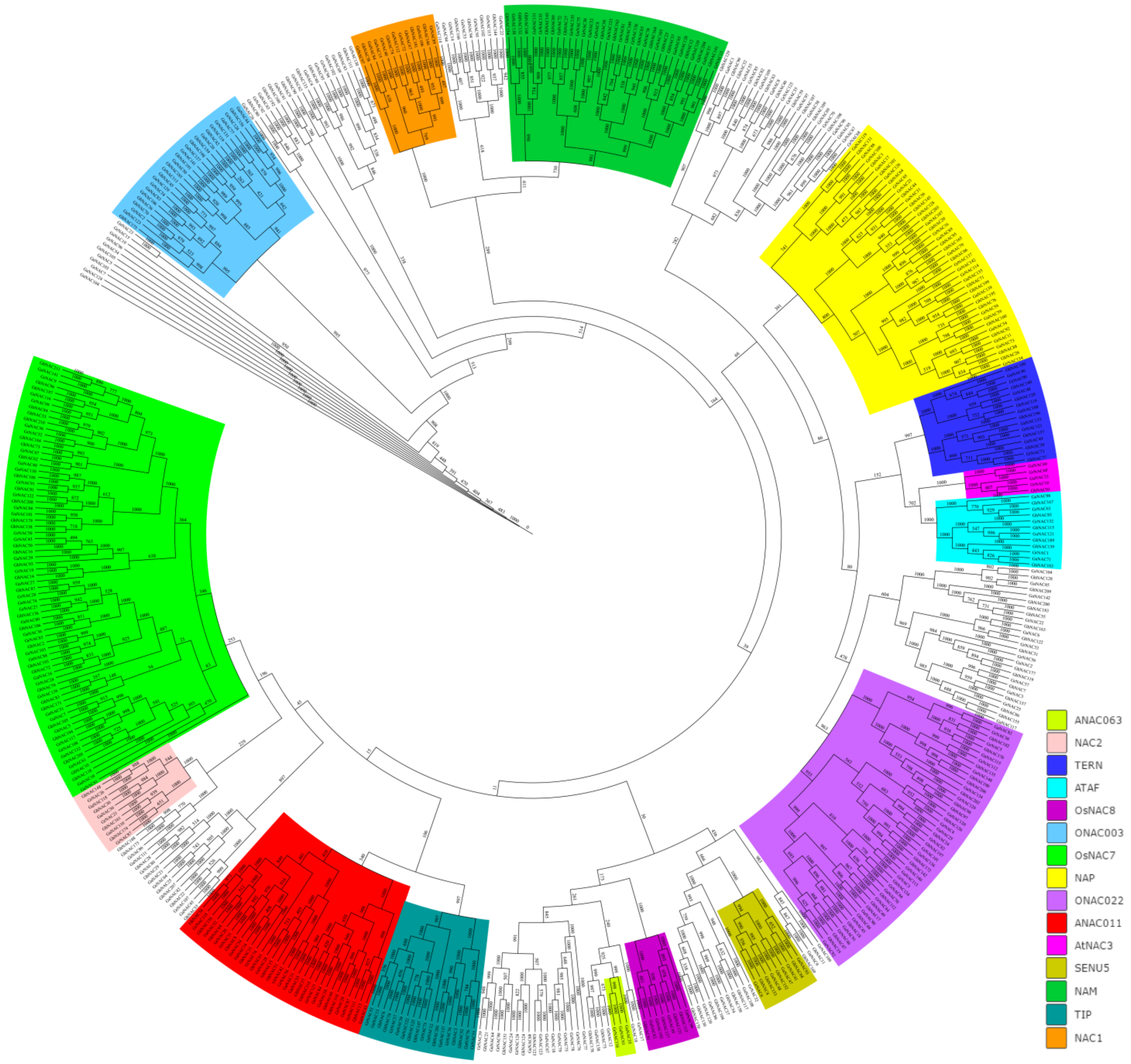

Fig. S2. Phylogenetic relationship of NAC proteins from *G.raimondii, G.arboreum and G.hirsutum*. Amino acid sequences were aligned with ClustalW and the neighbor joining (NJ) tree was generated through PHYLIP package. The numbers in the clades are the bootstrap values. The subfamilies within the NAC family are grouped by colors.

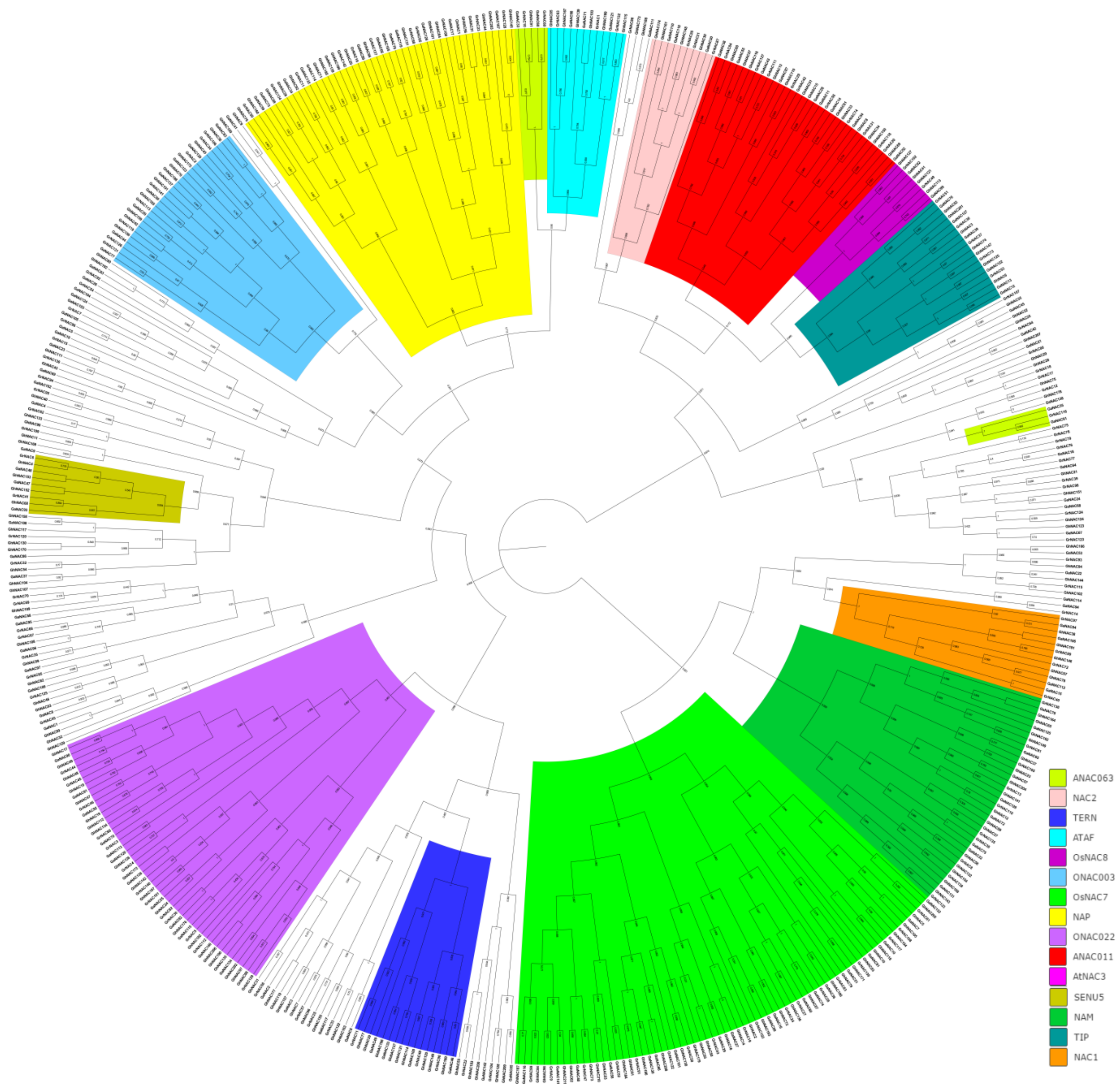

Fig. S3. Phylogenetic analysis of GaNACs, GrNACs and GhNACs. The phylogenetic tree was constructed with the Fasttree method. The tree is unrooted. The numbers in the clades are the FastTree bootstrap values. Each NAC subfamily are indicated in a specific color.

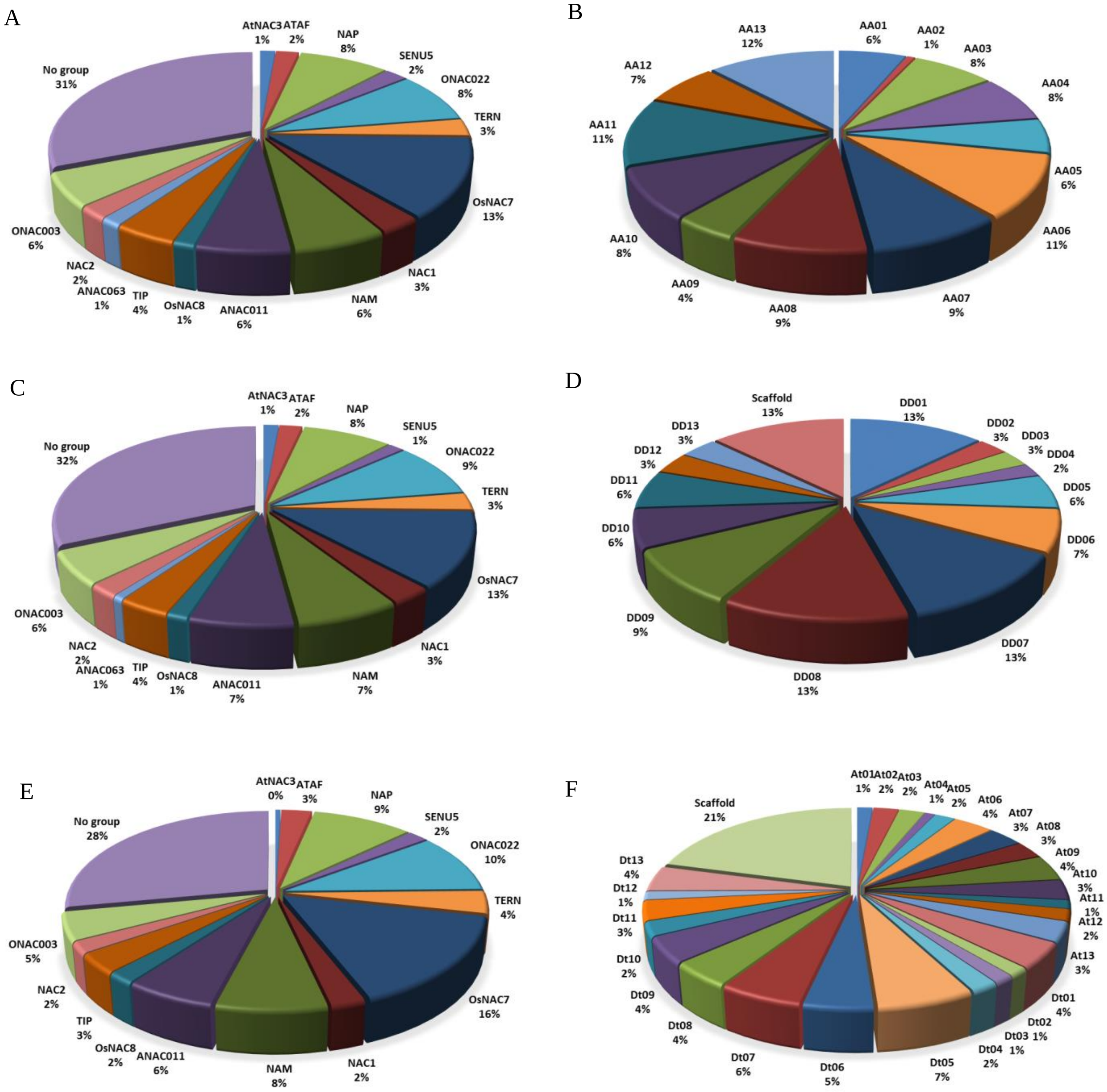

Fig. S4. The percentage of NAC subfamily (A, C, E) and its chromosomes localizations (B, D, F) in *G.arboretum* (A-B), *G.raimondii* (C-D) and *G.hirsutum* (E-F).

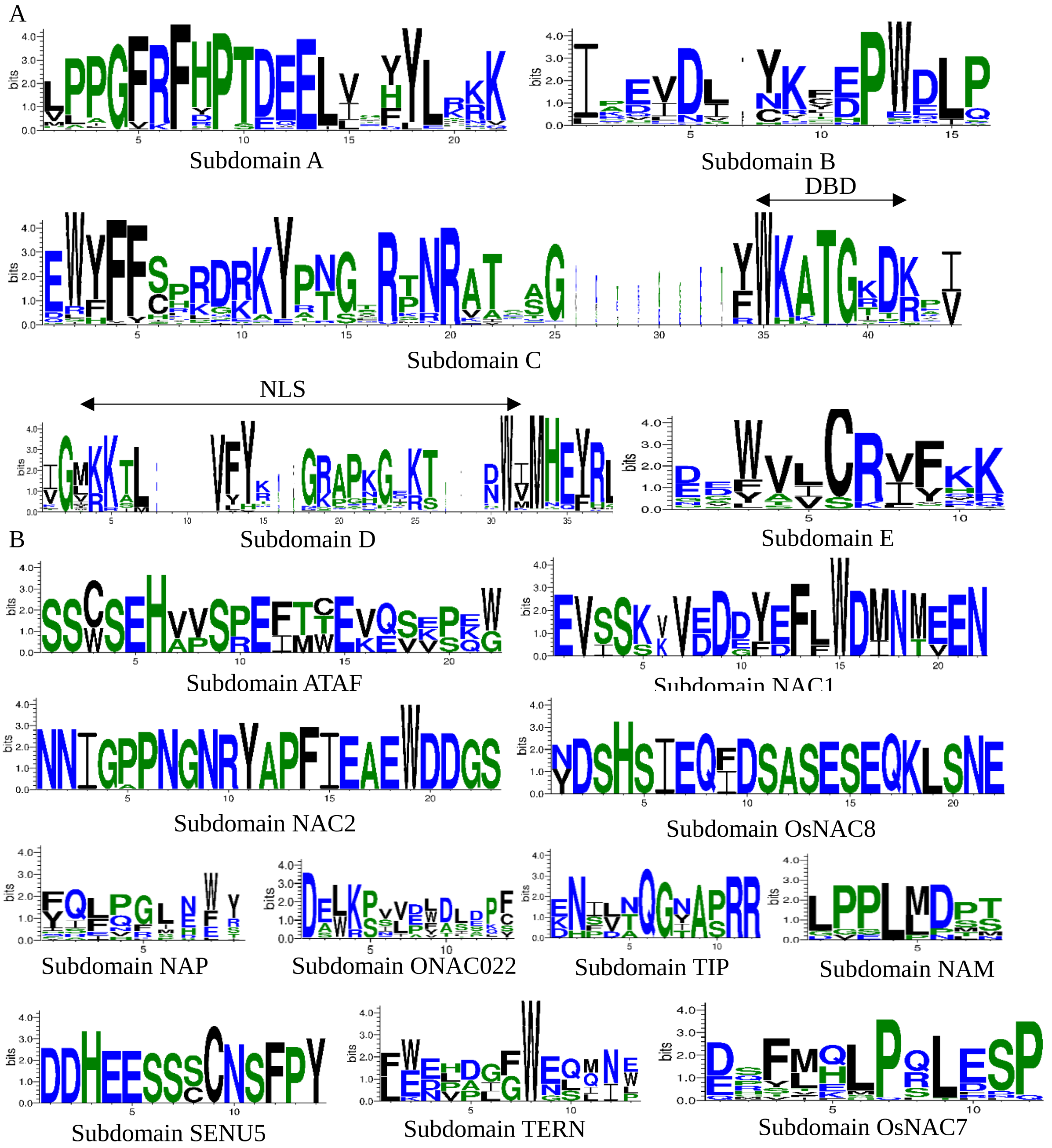

Fig. S5. The conserved domain analysis of NAC proteins in *G.arboretum*, *G.raimondii* and *G.hirsutum*. Sequence logos of NAC domain (A) and TAR region (B) were shown by WebLogo program. The height of letter designating the amino acid residue at each position represents the degree of conservation. The numbers on the x-axis represent the sequence positions in its corresponding conservative domains. The y-axis represents the information content measured in bits.

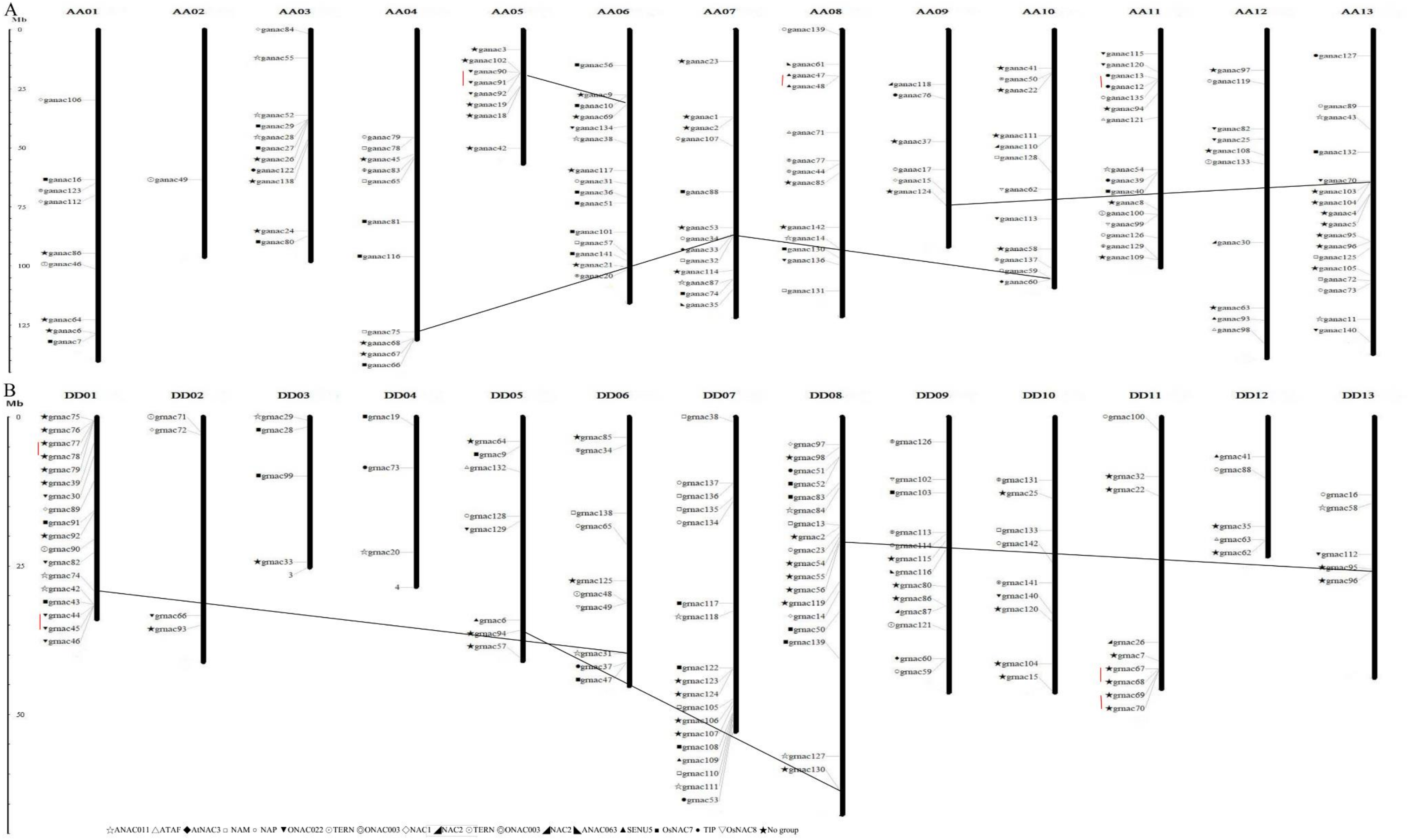

Fig. S6 Chromosomal locations of GaNAC (A) and GrNAC (B) on all 13 chromosomes. The scale is megabases (Mb). Markers before the gene names indicate the NAC subfamily. The red lines mark the tandem duplication, and genes related to segmental duplication are joined by gray lines.

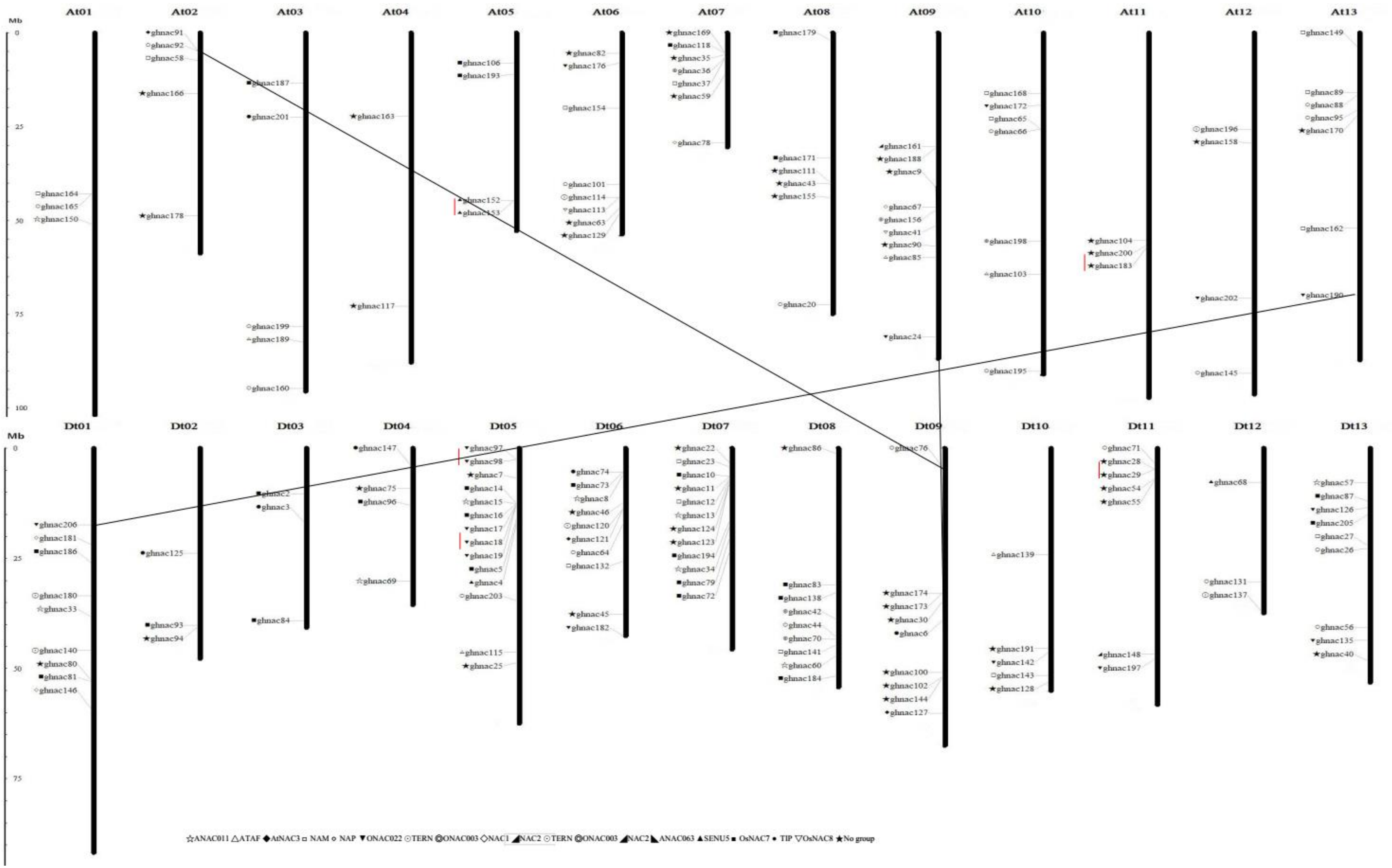

Fig.S7 Chromosomal locations of GhNAC on the 26 chromosomes. The scale is megabases (Mb). Markers before the gene names indicate the NAC subfamily. The red lines mark the tandem duplication, and genes related to segmental duplication are joined by gray lines.

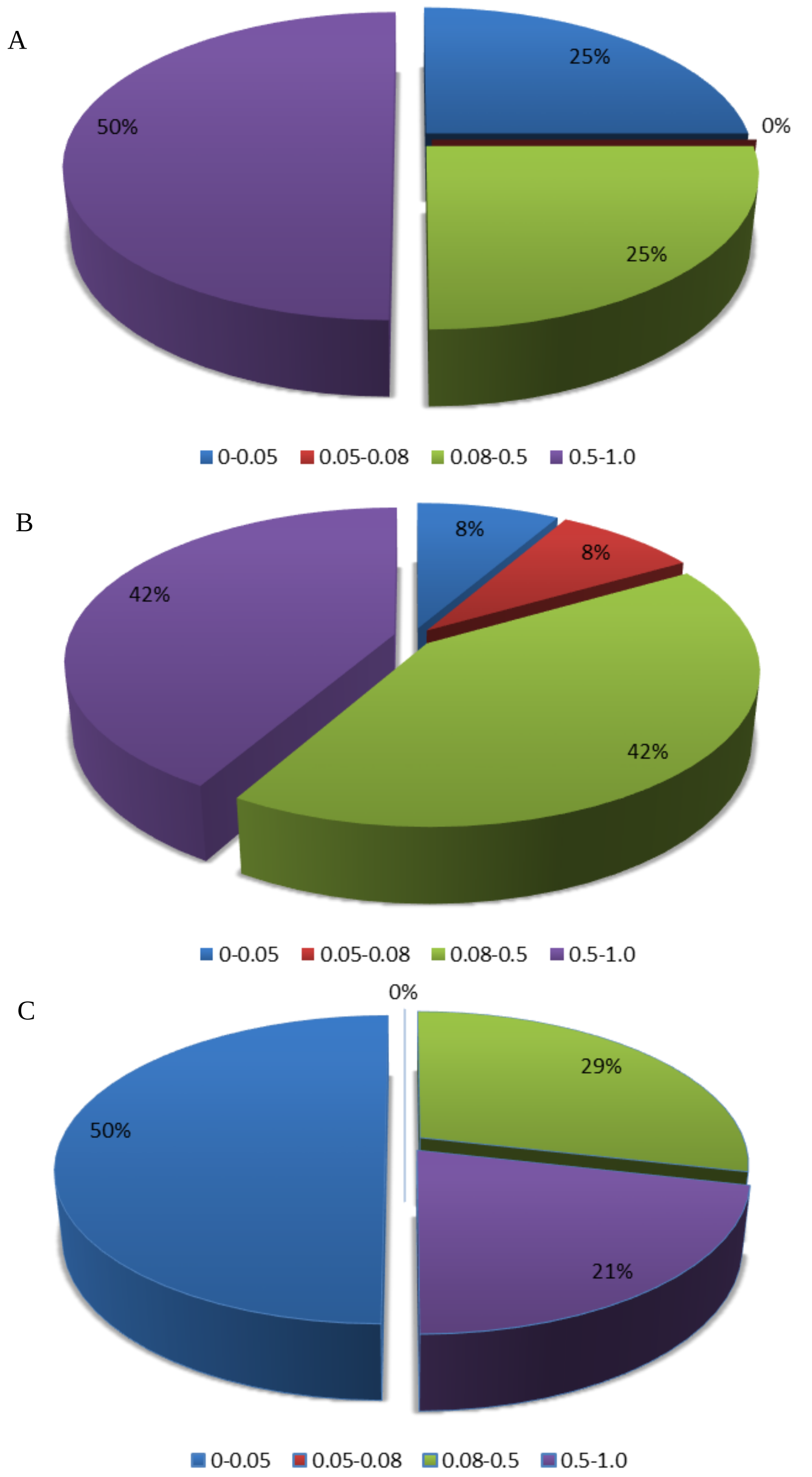

Fig. S8. Age distribution of the duplicated GaNACs (A), GrNACs (B), and GhNACs (C) based on Ks values.
