# Supplementary material for: Asymmetric Evolution and Expansion of the NAC Transcription Factor in Polyploidized Cotton
Source: Front Plant Sci. 2018 Jan 30;9:47. doi: 10.3389/fpls.2018.00047 (PMC5797638; doi:10.3389/fpls.2018.00047)
Supplement: Table S1 — NAC proteins identified in G.raimondii, G.arboreum, and G.hirsutum. [file Table1.PDF]

TABLE S1 NAC proteins identified in *G.raimondii, G.arboreum* and *G.hirsutum*.

Name	Locus name	Subfamily	E-value	Score
GrNAC1	Cotton_D_gene_10000398	ATAF	8.90E-28	98.1
GrNAC2	Cotton_D_gene_10001169	ONAC003	1.20E-13	52.8
GrNAC3	Cotton_D_gene_10001389	ONAC022	4.00E-25	89.6
GrNAC4	Cotton_D_gene_10001402	ONAC022	2.50E-23	83.9
GrNAC5	Cotton_D_gene_10001781	ONAC022	2.10E-25	90.5
GrNAC6	Cotton_D_gene_10001806	SENU5	6.60E-27	95.3
GrNAC7	Cotton_D_gene_10002720	No group	2.90E-13	51.6
GrNAC8	Cotton_D_gene_10003338	NAM	1.60E-28	100.5
GrNAC9	Cotton_D_gene_10003415	OsNAC7	1.80E-26	94
GrNAC10	Cotton_D_gene_10003677	AtNAC3	5.70E-26	92.3
GrNAC11	Cotton_D_gene_10003679	NAP	2.60E-28	99.8
GrNAC12	Cotton_D_gene_10004057	No group	1.90E-21	77.9
GrNAC13	Cotton_D_gene_10004296	NAM	2.20E-29	103.3
GrNAC14	Cotton_D_gene_10004507	No group	4.20E-22	80
GrNAC15	Cotton_D_gene_10006133	No group	6.80E-18	66.5
GrNAC16	Cotton_D_gene_10006239	NAP	1.40E-26	94.3
GrNAC17	Cotton_D_gene_10006331	No group	2.50E-15	58.2
GrNAC18	Cotton_D_gene_10006337	No group	1.50E-15	59
GrNAC19	Cotton_D_gene_10007297	OsNAC7	1.90E-27	97
GrNAC20	Cotton_D_gene_10007865	ANAC011	1.10E-30	107.4
GrNAC21	Cotton_D_gene_10008434	NAC2	1.70E-27	97.3
GrNAC22	Cotton_D_gene_10008587	No group	9.80E-25	88.4
GrNAC23	Cotton_D_gene_10008897	NAP	3.70E-27	96.1
GrNAC24	Cotton_D_gene_10009406	OsNAC7	1.10E-28	101
GrNAC25	Cotton_D_gene_10009504	No group	1.60E-25	90.9
GrNAC26	Cotton_D_gene_10009519	NAC2	1.20E-27	97.8
GrNAC27	Cotton_D_gene_10009574	OsNAC7	1.90E-28	100.2
GrNAC28	Cotton_D_gene_10009764	OsNAC7	1.40E-28	100.7
GrNAC29	Cotton_D_gene_10009873	ANAC011	1.00E-28	101.1
GrNAC30	Cotton_D_gene_10010003	ONAC022	4.50E-26	92.7
GrNAC31	Cotton_D_gene_10010022	ANAC011	7.80E-30	104.7
GrNAC32	Cotton_D_gene_10010808	No group	2.10E-21	77.7
GrNAC33	Cotton_D_gene_10011876	No group	1.10E-25	91.4
GrNAC34	Cotton_D_gene_10011908	ONAC003	3.90E-13	51.2
GrNAC35	Cotton_D_gene_10012088	No group	1.00E-21	78.7
GrNAC36	Cotton_D_gene_10012458	TIP	1.30E-26	94.4
GrNAC37	Cotton_D_gene_10012613	TIP	2.50E-27	96.7
GrNAC38	Cotton_D_gene_10012678	NAM	2.10E-28	100.1
GrNAC39	Cotton_D_gene_10013218	No group	4.60E-20	73.4
GrNAC40	Cotton_D_gene_10013771	NAC1	7.80E-28	98.3
GrNAC41	Cotton_D_gene_10014197	SENU5	1.10E-26	94.6
GrNAC42	Cotton_D_gene_10015217	ANAC011	1.80E-28	100.4

GrNAC43	Cotton_D_gene_10015218	OsNAC7	5.50E-28	98.8
GrNAC44	Cotton_D_gene_10015235	ONAC022	2.70E-24	87
GrNAC45	Cotton_D_gene_10015236	ONAC022	2.70E-25	90.2
GrNAC46	Cotton_D_gene_10015237	ONAC022	8.10E-25	88.6
GrNAC47	Cotton_D_gene_10015370	OsNAC7	2.40E-26	93.6
GrNAC48	Cotton_D_gene_10015775	TERN	2.40E-22	80.7
GrNAC49	Cotton_D_gene_10015783	OsNAC8	4.60E-25	89.4
GrNAC50	Cotton_D_gene_10015938	OsNAC7	7.70E-27	95.1
GrNAC51	Cotton_D_gene_10016783	TIP	1.70E-27	97.2
GrNAC52	Cotton_D_gene_10016798	OsNAC7	1.20E-26	94.6
GrNAC53	Cotton_D_gene_10017774	TIP	7.70E-27	95.1
GrNAC54	Cotton_D_gene_10018007	No group	1.60E-11	46
GrNAC55	Cotton_D_gene_10018120	No group	1.80E-23	84.3
GrNAC56	Cotton_D_gene_10018185	No group	1.20E-24	88.1
GrNAC57	Cotton_D_gene_10018455	No group	2.90E-24	86.9
GrNAC58	Cotton_D_gene_10018978	ANAC011	5.80E-29	101.9
GrNAC59	Cotton_D_gene_10019247	NAP	6.30E-28	98.6
GrNAC60	Cotton_D_gene_10019250	AtNAC3	3.60E-26	93
GrNAC61	Cotton_D_gene_10019529	OsNAC7	4.70E-22	79.8
GrNAC62	Cotton_D_gene_10019770	No group	5.60E-21	76.3
GrNAC63	Cotton_D_gene_10019785	ATAF	9.70E-16	59.5
GrNAC64	Cotton_D_gene_10019947	No group	3.20E-27	96.4
GrNAC65	Cotton_D_gene_10020643	NAP	4.40E-26	92.7
GrNAC66	Cotton_D_gene_10020922	ONAC022	1.90E-25	90.7
GrNAC67	Cotton_D_gene_10021541	No group	7.80E-26	91.9
GrNAC68	Cotton_D_gene_10021542	No group	3.40E-24	86.6
GrNAC69	Cotton_D_gene_10021572	No group	1.20E-22	81.7
GrNAC70	Cotton_D_gene_10021575	No group	1.00E-22	81.9
GrNAC71	Cotton_D_gene_10021859	TERN	8.60E-23	82.1
GrNAC72	Cotton_D_gene_10021906	NAC1	1.30E-27	97.6
GrNAC73	Cotton_D_gene_10022615	TIP	5.00E-27	95.7
GrNAC74	Cotton_D_gene_10022691	ANAC011	2.20E-30	106.5
GrNAC75	Cotton_D_gene_10023133	No group	1.90E-07	33
GrNAC76	Cotton_D_gene_10023135	No group	3.50E-07	32.1
GrNAC77	Cotton_D_gene_10023137	No group	5.80E-08	34.6
GrNAC78	Cotton_D_gene_10023138	No group	3.40E-07	32.2
GrNAC79	Cotton_D_gene_10023139	No group	3.90E-06	28.8
GrNAC80	Cotton_D_gene_10023489	No group	1.60E-27	97.3
GrNAC81	Cotton_D_gene_10024033	NAM	1.00E-29	104.3
GrNAC82	Cotton_D_gene_10024070	ONAC022	5.60E-25	89.2
GrNAC83	Cotton_D_gene_10024267	OsNAC7	1.80E-28	100.3
GrNAC84	Cotton_D_gene_10024357	ANAC011	7.60E-29	101.5
GrNAC85	Cotton_D_gene_10026995	No group	1.30E-23	84.7
GrNAC86	Cotton_D_gene_10027600	No group	7.90E-28	98.3

GrNAC87	Cotton_D_gene_10027602	NAC2	8.10E-28	98.3
GrNAC88	Cotton_D_gene_10027798	NAP	1.40E-27	97.5
GrNAC89	Cotton_D_gene_10028025	NAC1	1.30E-27	97.6
GrNAC90	Cotton_D_gene_10028293	TERN	1.50E-13	52.6
GrNAC91	Cotton_D_gene_10028537	OsNAC7	1.30E-27	97.6
GrNAC92	Cotton_D_gene_10028546	No group	5.50E-20	73.2
GrNAC93	Cotton_D_gene_10028772	No group	5.00E-27	95.7
GrNAC94	Cotton_D_gene_10029594	No group	4.50E-23	83.1
GrNAC95	Cotton_D_gene_10030000	No group	9.60E-24	85.2
GrNAC96	Cotton_D_gene_10030033	No group	2.20E-13	52
GrNAC97	Cotton_D_gene_10030897	NAC1	4.10E-27	96
GrNAC98	Cotton_D_gene_10030952	No group	1.90E-19	71.4
GrNAC99	Cotton_D_gene_10031000	OsNAC7	1.40E-26	94.3
GrNAC100	Cotton_D_gene_10031395	NAP	1.30E-24	88
GrNAC101	Cotton_D_gene_10031650	ONAC022	1.40E-18	68.6
GrNAC102	Cotton_D_gene_10032044	OsNAC8	1.00E-24	88.4
GrNAC103	Cotton_D_gene_10032344	OsNAC7	6.30E-28	98.6
GrNAC104	Cotton_D_gene_10032427	No group	1.40E-24	87.8
GrNAC105	Cotton_D_gene_10032729	NAM	5.00E-29	102.1
GrNAC106	Cotton_D_gene_10032775	ONAC003	9.70E-12	46.7
GrNAC107	Cotton_D_gene_10032869	No group	6.50E-25	88.9
GrNAC108	Cotton_D_gene_10033034	OsNAC7	3.50E-19	70.6
GrNAC109	Cotton_D_gene_10033076	No group	2.60E-24	87
GrNAC110	Cotton_D_gene_10033135	NAM	5.50E-29	102
GrNAC111	Cotton_D_gene_10033204	ANAC011	1.80E-28	100.3
GrNAC112	Cotton_D_gene_10033523	ONAC022	1.10E-25	91.4
GrNAC113	Cotton_D_gene_10033716	ONAC003	3.40E-15	57.8
GrNAC114	Cotton_D_gene_10033762	NAP	4.30E-28	99.1
GrNAC115	Cotton_D_gene_10033915	No group	1.20E-27	97.8
GrNAC116	Cotton_D_gene_10034094	ANAC063	8.40E-06	27.7
GrNAC117	Cotton_D_gene_10034381	OsNAC7	3.50E-19	70.6
GrNAC118	Cotton_D_gene_10034463	ANAC011	2.40E-30	106.4
GrNAC119	Cotton_D_gene_10034768	ONAC003	9.90E-16	59.5
GrNAC120	Cotton_D_gene_10034990	No group	2.20E-19	71.2
GrNAC121	Cotton_D_gene_10035259	TERN	3.10E-20	74
GrNAC122	Cotton_D_gene_10035732	OsNAC7	2.20E-27	96.9
GrNAC123	Cotton_D_gene_10035749	No group	1.50E-18	68.5
GrNAC124	Cotton_D_gene_10035750	No group	1.90E-21	77.9
GrNAC125	Cotton_D_gene_10036070	No group	7.40E-25	88.8
GrNAC126	Cotton_D_gene_10037389	ONAC003	2.80E-16	61.3
GrNAC127	Cotton_D_gene_10037770	ANAC011	1.10E-28	101.1
GrNAC128	Cotton_D_gene_10037884	NAP	7.40E-27	95.2
GrNAC129	Cotton_D_gene_10037901	ONAC022	2.00E-25	90.6
GrNAC130	Cotton_D_gene_10038188	No group	3.50E-23	83.4

GrNAC131	Cotton_D_gene_10039159	ONAC003	2.10E-16	61.7
GrNAC132	Cotton_D_gene_10039294	ATAF	2.90E-27	96.5
GrNAC133	Cotton_D_gene_10039623	NAM	4.00E-28	99.2
GrNAC134	Cotton_D_gene_10039854	NAP	2.90E-28	99.7
GrNAC135	Cotton_D_gene_10039891	NAM	3.40E-28	99.5
GrNAC136	Cotton_D_gene_10039991	NAM	9.30E-30	104.5
GrNAC137	Cotton_D_gene_10039993	NAP	7.60E-28	98.3
GrNAC138	Cotton_D_gene_10040174	NAM	1.10E-28	101.1
GrNAC139	Cotton_D_gene_10040323	OsNAC7	1.40E-28	100.7
GrNAC140	Cotton_D_gene_10040655	ONAC022	1.50E-25	91
GrNAC141	Cotton_D_gene_10040814	ONAC003	7.50E-15	56.7
GrNAC142	Cotton_D_gene_10040904	NAP	5.70E-29	102
GaNAC1	Cotton_A_00183	No group	1.80E-23	84.3
GaNAC2	Cotton_A_00256	No group	1.20E-24	88.1
GaNAC3	Cotton_A_00602	No group	2.60E-25	90.2
GaNAC4	Cotton_A_00831	No group	4.40E-24	86.2
GaNAC5	Cotton_A_00841	No group	4.20E-05	25.4
GaNAC6	Cotton_A_01505	No group	2.50E-25	90.3
GaNAC7	Cotton_A_01569	OsNAC7	8.00E-19	69.4
GaNAC8	Cotton_A_01813	No group	6.70E-25	88.9
GaNAC9	Cotton_A_01943	No group	2.70E-24	86.9
GaNAC10	Cotton_A_01989	OsNAC7	2.30E-29	103.2
GaNAC11	Cotton_A_02191	ANAC011	6.20E-29	101.8
GaNAC12	Cotton_A_02365	TIP	9.70E-28	98
GaNAC13	Cotton_A_02366	TIP	7.70E-27	95.1
GaNAC14	Cotton_A_02718	ANAC011	2.80E-30	106.1
GaNAC15	Cotton_A_03013	NAC1	1.40E-27	97.5
GaNAC16	Cotton_A_03573	OsNAC7	1.10E-28	101
GaNAC17	Cotton_A_03930	NAP	3.90E-24	86.4
GaNAC18	Cotton_A_04401	No group	1.30E-06	30.3
GaNAC19	Cotton_A_04422	No group	4.30E-10	41.4
GaNAC20	Cotton_A_04481	ONAC003	9.60E-16	59.5
GaNAC21	Cotton_A_04509	No group	1.90E-27	97
GaNAC22	Cotton_A_04612	No group	8.00E-28	98.2
GaNAC23	Cotton_A_04939	No group	2.10E-17	64.9
GaNAC24	Cotton_A_05099	No group	4.70E-20	73.3
GaNAC25	Cotton_A_05272	ONAC022	1.20E-25	91.3
GaNAC26	Cotton_A_05740	No group	5.70E-18	66.7
GaNAC27	Cotton_A_05832	OsNAC7	2.90E-28	99.6
GaNAC28	Cotton_A_05844	ANAC011	1.70E-28	100.4
GaNAC29	Cotton_A_05845	OsNAC7	5.20E-28	98.8
GaNAC30	Cotton_A_06086	NAC2	1.70E-27	97.2
GaNAC31	Cotton_A_06161	NAP	3.60E-27	96.1
GaNAC32	Cotton_A_06256	NAM	1.50E-28	100.5

GaNAC33	Cotton_A_06324	AtNAC3	5.70E-26	92.3
GaNAC34	Cotton_A_06325	NAP	2.60E-28	99.8
GaNAC35	Cotton_A_06877	ANAC063	2.40E-05	26.2
GaNAC36	Cotton_A_07124	OsNAC7	1.50E-28	100.6
GaNAC37	Cotton_A_07511	No group	7.10E-21	76
GaNAC38	Cotton_A_07894	ANAC011	9.80E-29	101.2
GaNAC39	Cotton_A_08115	TIP	5.30E-27	95.6
GaNAC40	Cotton_A_08128	OsNAC7	6.00E-27	95.4
GaNAC41	Cotton_A_08671	No group	1.90E-16	61.8
GaNAC42	Cotton_A_08816	No group	2.50E-27	96.7
GaNAC43	Cotton_A_09163	ANAC011	1.70E-28	100.4
GaNAC44	Cotton_A_09393	ONAC003	2.80E-16	61.3
GaNAC45	Cotton_A_09515	No group	3.20E-25	89.9
GaNAC46	Cotton_A_09599	TERN	3.60E-23	83.3
GaNAC47	Cotton_A_10020	SENU5	7.00E-27	95.2
GaNAC48	Cotton_A_10022	SENU5	7.20E-27	95.2
GaNAC49	Cotton_A_10170	TERN	6.10E-23	82.6
GaNAC50	Cotton_A_11313	ONAC003	3.30E-15	57.8
GaNAC51	Cotton_A_11365	OsNAC7	3.00E-29	102.8
GaNAC52	Cotton_A_12607	ANAC011	1.20E-30	107.3
GaNAC53	Cotton_A_12683	No group	4.60E-27	95.8
GaNAC54	Cotton_A_13074	ANAC011	3.20E-30	105.9
GaNAC55	Cotton_A_13127	ANAC011	2.00E-28	100.2
GaNAC56	Cotton_A_13198	OsNAC7	1.30E-26	94.4
GaNAC57	Cotton_A_13395	NAM	2.70E-29	102.9
GaNAC58	Cotton_A_13494	No group	4.70E-18	66.9
GaNAC59	Cotton_A_13667	NAP	1.10E-27	97.8
GaNAC60	Cotton_A_13669	AtNAC3	3.90E-26	92.8
GaNAC61	Cotton_A_13728	ANAC063	1.20E-05	27.1
GaNAC62	Cotton_A_13859	OsNAC8	9.10E-25	88.4
GaNAC63	Cotton_A_14093	No group	1.90E-19	71.4
GaNAC64	Cotton_A_15184	No group	9.20E-20	72.4
GaNAC65	Cotton_A_15474	NAM	4.90E-29	102.1
GaNAC66	Cotton_A_15646	OsNAC7	2.10E-27	96.9
GaNAC67	Cotton_A_15664	No group	5.50E-18	66.7
GaNAC68	Cotton_A_15665	No group	2.00E-21	77.7
GaNAC69	Cotton_A_15785	No group	2.10E-23	84.1
GaNAC70	Cotton_A_15893	ONAC022	1.70E-25	90.8
GaNAC71	Cotton_A_16296	ATAF	8.60E-28	98.1
GaNAC72	Cotton_A_16773	NAM	1.70E-28	100.4
GaNAC73	Cotton_A_16813	NAP	2.80E-28	99.7
GaNAC74	Cotton_A_17125	OsNAC7	1.30E-28	100.8
GaNAC75	Cotton_A_17275	NAM	1.10E-28	101
GaNAC76	Cotton_A_17362	TIP	1.10E-27	97.8

GaNAC77	Cotton_A_18089	ONAC003	2.40E-16	61.5
GaNAC78	Cotton_A_18123	NAM	1.10E-29	104.2
GaNAC79	Cotton_A_18125	NAP	6.80E-28	98.5
GaNAC80	Cotton_A_18426	OsNAC7	7.00E-29	101.6
GaNAC81	Cotton_A_19336	OsNAC7	1.00E-29	104.3
GaNAC82	Cotton_A_19848	ONAC022	7.80E-26	91.9
GaNAC83	Cotton_A_20638	ONAC003	1.20E-12	49.6
GaNAC84	Cotton_A_20658	NAC1	4.00E-27	96
GaNAC85	Cotton_A_21002	No group	1.50E-24	87.8
GaNAC86	Cotton_A_22700	No group	1.60E-19	71.7
GaNAC87	Cotton_A_23128	ANAC011	7.40E-29	101.6
GaNAC88	Cotton_A_23892	OsNAC7	8.70E-28	98.1
GaNAC89	Cotton_A_24225	NAP	1.30E-26	94.3
GaNAC90	Cotton_A_24792	ONAC022	2.10E-24	87.3
GaNAC91	Cotton_A_24793	ONAC022	2.70E-25	90.1
GaNAC92	Cotton_A_24794	ONAC022	8.80E-25	88.5
GaNAC93	Cotton_A_24945	SENU5	9.40E-27	94.8
GaNAC94	Cotton_A_25050	No group	1.00E-26	94.7
GaNAC95	Cotton_A_25101	No group	4.40E-25	89.5
GaNAC96	Cotton_A_25115	No group	5.10E-24	86.1
GaNAC97	Cotton_A_25243	No group	1.40E-21	78.2
GaNAC98	Cotton_A_25450	ATAF	2.20E-27	96.8
GaNAC99	Cotton_A_26263	OsNAC8	4.10E-25	89.6
GaNAC100	Cotton_A_26270	TERN	6.10E-22	79.4
GaNAC101	Cotton_A_26426	OsNAC7	7.20E-27	95.2
GaNAC102	Cotton_A_27343	No group	5.60E-23	82.7
GaNAC103	Cotton_A_27805	No group	2.00E-13	52.1
GaNAC104	Cotton_A_27806	No group	9.90E-14	53.1
GaNAC105	Cotton_A_28228	No group	1.60E-12	49.2
GaNAC106	Cotton_A_28334	NAC1	1.30E-27	97.6
GaNAC107	Cotton_A_28452	NAP	6.70E-27	95.3
GaNAC108	Cotton_A_29050	No group	6.10E-20	73
GaNAC109	Cotton_A_29108	No group	4.90E-24	86.1
GaNAC110	Cotton_A_29173	NAC2	1.60E-27	97.3
GaNAC111	Cotton_A_29176	No group	9.00E-28	98.1
GaNAC112	Cotton_A_29202	NAC1	1.20E-27	97.7
GaNAC113	Cotton_A_29358	ONAC022	1.60E-25	90.9
GaNAC114	Cotton_A_30404	No group	1.30E-26	94.3
GaNAC115	Cotton_A_30603	ONAC022	2.10E-25	90.5
GaNAC116	Cotton_A_31522	OsNAC7	1.30E-26	94.4
GaNAC117	Cotton_A_32448	No group	1.60E-25	90.9
GaNAC118	Cotton_A_32478	NAC2	7.90E-28	98.3
GaNAC119	Cotton_A_32870	NAP	1.00E-27	97.9
GaNAC120	Cotton_A_33158	ONAC022	2.70E-24	87

GaNAC121	Cotton_A_33477	ATAF	1.40E-26	94.3
GaNAC122	Cotton_A_33555	TIP	2.80E-27	96.5
GaNAC123	Cotton_A_34465	ONAC003	1.20E-13	52.8
GaNAC124	Cotton_A_34560	No group	4.30E-14	54.2
GaNAC125	Cotton_A_35053	NAM	1.00E-29	104.3
GaNAC126	Cotton_A_35198	NAP	1.10E-26	94.6
GaNAC127	Cotton_A_35235	TIP	1.50E-26	94.2
GaNAC128	Cotton_A_35583	NAM	5.40E-29	102
GaNAC129	Cotton_A_35637	ONAC003	2.50E-13	51.8
GaNAC130	Cotton_A_36063	OsNAC7	1.50E-27	97.3
GaNAC131	Cotton_A_36235	NAM	4.00E-28	99.2
GaNAC132	Cotton_A_36402	OsNAC7	1.70E-18	68.3
GaNAC133	Cotton_A_36420	TERN	6.10E-22	79.4
GaNAC134	Cotton_A_36695	ONAC022	1.80E-25	90.7
GaNAC135	Cotton_A_36752	NAP	4.20E-28	99.1
GaNAC136	Cotton_A_37610	ONAC022	1.40E-25	91
GaNAC137	Cotton_A_37659	ONAC003	8.10E-15	56.6
GaNAC138	Cotton_A_38266	No group	1.80E-21	77.9
GaNAC139	Cotton_A_38770	NAP	1.00E-28	101.1
GaNAC140	Cotton_A_39298	ONAC022	1.00E-25	91.5
GaNAC141	Cotton_A_39966	OsNAC7	1.70E-26	94
GaNAC142	Cotton_A_40150	No group	7.80E-25	88.7
GhNAC1	CotAD_00653	NAP	7.40E-24	86.4
GhNAC2	CotAD_01815	OsNAC7	1.20E-27	98.6
GhNAC3	CotAD_02099	TIP	2.80E-26	94.2
GhNAC4	CotAD_02200	SENU5	1.20E-26	95.4
GhNAC5	CotAD_02309	OsNAC7	1.50E-18	69.4
GhNAC6	CotAD_02648	TIP	2.50E-26	94.4
GhNAC7	CotAD_03014	No group	6.90E-25	89.7
GhNAC8	CotAD_03188	ANAC011	1.90E-29	104.4
GhNAC9	CotAD_03657	No group	5.60E-16	61.2
GhNAC10	CotAD_03788	OsNAC7	4.40E-29	103.2
GhNAC11	CotAD_03831	No group	5.00E-22	80.6
GhNAC12	CotAD_03895	NAM	1.10E-28	101.9
GhNAC13	CotAD_03965	ANAC011	3.40E-28	100.3
GhNAC14	CotAD_04129	OsNAC7	3.60E-27	97
GhNAC15	CotAD_04141	ANAC011	3.40E-28	100.4
GhNAC16	CotAD_04142	OsNAC7	1.80E-25	91.6
GhNAC17	CotAD_04161	ONAC022	4.50E-24	87.1
GhNAC18	CotAD_04162	ONAC022	5.30E-25	90.1
GhNAC19	CotAD_04163	ONAC022	1.50E-24	88.7
GhNAC20	CotAD_04459	NAP	2.60E-26	94.3
GhNAC21	CotAD_04813	No group	1.60E-19	72.6
GhNAC22	CotAD_05367	No group	1.20E-24	89

GhNAC23	CotAD_05449	NAM	8.90E-29	102.2
GhNAC24	CotAD_05613	ONAC022	2.10E-25	91.4
GhNAC25	CotAD_05908	No group	1.70E-26	94.9
GhNAC26	CotAD_06080	NAP	6.70E-28	99.4
GhNAC27	CotAD_06127	NAM	6.10E-28	99.5
GhNAC28	CotAD_06342	No group	2.80E-27	97.4
GhNAC29	CotAD_06343	No group	1.30E-27	98.5
GhNAC30	CotAD_08094	NAC2	3.70E-24	87.4
GhNAC31	CotAD_08843	No group	9.50E-23	82.9
GhNAC32	CotAD_08908	No group	3.90E-23	84.1
GhNAC33	CotAD_09032	ANAC011	4.00E-30	106.5
GhNAC34	CotAD_09298	ANAC011	4.90E-30	106.2
GhNAC35	CotAD_10002	No group	6.30E-25	89.9
GhNAC36	CotAD_10103	ONAC003	1.10E-12	50.6
GhNAC37	CotAD_10149	NAM	9.30E-29	102.1
GhNAC38	CotAD_11289	NAC1	7.70E-27	96
GhNAC39	CotAD_11909	TERN	1.60E-22	82.1
GhNAC40	CotAD_12132	No group	1.90E-23	85.1
GhNAC41	CotAD_12370	OsNAC8	1.90E-24	88.3
GhNAC42	CotAD_12779	ONAC003	1.90E-15	59.5
GhNAC43	CotAD_13031	No group	3.50E-23	84.3
GhNAC44	CotAD_13212	NAP	7.00E-27	96.1
GhNAC45	CotAD_13229	ONAC003	8.00E-13	51.1
GhNAC46	CotAD_13616	No group	1.70E-24	88.5
GhNAC47	CotAD_14694	ONAC022	1.60E-24	88.6
GhNAC48	CotAD_14695	ONAC022	5.00E-25	90.2
GhNAC49	CotAD_14696	ONAC022	5.40E-24	86.9
GhNAC50	CotAD_14713	OsNAC7	1.00E-27	98.8
GhNAC51	CotAD_14714	ANAC011	3.30E-28	100.4
GhNAC52	CotAD_15346	TIP	1.90E-27	98
GhNAC53	CotAD_15362	OsNAC7	9.70E-25	89.3
GhNAC54	CotAD_15553	No group	1.10E-20	76.3
GhNAC55	CotAD_15579	No group	1.80E-24	88.4
GhNAC56	CotAD_16206	NAP	6.90E-27	96.1
GhNAC57	CotAD_16600	ANAC011	3.00E-28	100.5
GhNAC58	CotAD_16876	NAM	2.50E-28	100.8
GhNAC59	CotAD_17446	No group	8.40E-20	73.4
GhNAC60	CotAD_17553	ANAC011	1.10E-28	101.9
GhNAC61	CotAD_17816	ANAC011	2.60E-30	107.1
GhNAC62	CotAD_18284	OsNAC7	1.20E-26	95.4
GhNAC63	CotAD_20051	No group	1.30E-24	88.9
GhNAC64	CotAD_20257	NAP	3.60E-26	93.9
GhNAC65	CotAD_20641	NAM	2.00E-29	104.3
GhNAC66	CotAD_20643	NAP	1.40E-27	98.3

GhNAC67	CotAD_20777	NAC1	7.30E-28	99.3
GhNAC68	CotAD_20843	SENU5	1.90E-26	94.8
GhNAC69	CotAD_21471	ANAC011	2.50E-30	107.2
GhNAC70	CotAD_22028	ONAC003	2.60E-13	52.6
GhNAC71	CotAD_22211	NAP	8.20E-28	99.1
GhNAC72	CotAD_22763	OsNAC7	2.10E-28	101
GhNAC73	CotAD_23565	OsNAC7	4.30E-26	93.6
GhNAC74	CotAD_23580	TIP	4.80E-27	96.7
GhNAC75	CotAD_23891	No group	4.10E-21	77.6
GhNAC76	CotAD_24346	NAP	1.20E-27	98.6
GhNAC77	CotAD_24523	TERN	7.40E-22	80
GhNAC78	CotAD_24873	NAC1	2.30E-27	97.7
GhNAC79	CotAD_25313	OsNAC7	1.00E-26	95.6
GhNAC80	CotAD_25813	No group	5.90E-19	70.7
GhNAC81	CotAD_25824	OsNAC7	3.10E-27	97.3
GhNAC82	CotAD_26175	No group	9.20E-24	86.1
GhNAC83	CotAD_27066	OsNAC7	3.20E-29	103.6
GhNAC84	CotAD_27466	OsNAC7	2.60E-26	94.3
GhNAC85	CotAD_27648	ATAF	5.20E-37	128.6
GhNAC86	CotAD_27788	No group	3.00E-25	90.9
GhNAC87	CotAD_27860	OsNAC7	2.60E-28	100.7
GhNAC88	CotAD_28105	NAP	5.40E-28	99.7
GhNAC89	CotAD_28138	NAM	6.50E-27	96.2
GhNAC90	CotAD_28513	No group	5.70E-21	77.2
GhNAC91	CotAD_28796	AtNAC3	9.90E-26	92.4
GhNAC92	CotAD_28797	NAP	4.40E-28	100
GhNAC93	CotAD_29099	OsNAC7	5.80E-22	80.4
GhNAC94	CotAD_29117	No group	9.60E-27	95.7
GhNAC95	CotAD_29662	NAP	2.50E-26	94.3
GhNAC96	CotAD_30367	OsNAC7	3.80E-26	93.8
GhNAC97	CotAD_30602	ONAC022	3.60E-25	90.7
GhNAC98	CotAD_30603	ONAC022	3.70E-25	90.6
GhNAC99	CotAD_31269	No group	3.10E-23	84.4
GhNAC100	CotAD_31337	No group	3.60E-16	61.8
GhNAC101	CotAD_31991	NAP	2.20E-26	94.6
GhNAC102	CotAD_32209	No group	2.20E-27	97.8
GhNAC103	CotAD_32858	ATAF	1.70E-27	98.1
GhNAC104	CotAD_33426	No group	3.80E-20	74.6
GhNAC105	CotAD_33904	OsNAC7	1.50E-18	69.4
GhNAC106	CotAD_34236	OsNAC7	2.90E-28	100.6
GhNAC107	CotAD_34624	No group	1.00E-22	82.7
GhNAC108	CotAD_34627	No group	3.60E-23	84.2
GhNAC109	CotAD_34628	No group	5.10E-22	80.6
GhNAC110	CotAD_34909	ANAC011	4.70E-28	99.9

GhNAC111	CotAD_34983	No group	1.50E-23	85.4
GhNAC112	CotAD_35736	ONAC022	3.50E-25	90.7
GhNAC113	CotAD_36338	OsNAC8	8.30E-25	89.5
GhNAC114	CotAD_36345	TERN	8.70E-22	79.8
GhNAC115	CotAD_36388	ATAF	5.40E-27	96.5
GhNAC116	CotAD_36441	ANAC011	1.30E-26	95.3
GhNAC117	CotAD_36819	No group	2.40E-19	72
GhNAC118	CotAD_37245	OsNAC7	7.60E-29	102.4
GhNAC119	CotAD_37972	No group	3.70E-24	87.4
GhNAC120	CotAD_38061	TERN	4.50E-22	80.7
GhNAC121	CotAD_38068	OsNAC8	8.70E-25	89.4
GhNAC122	CotAD_38778	No group	2.10E-25	91.4
GhNAC123	CotAD_39078	No group	2.20E-18	68.9
GhNAC124	CotAD_39079	No group	2.40E-21	78.4
GhNAC125	CotAD_39272	TIP	5.70E-27	96.4
GhNAC126	CotAD_39755	ONAC022	6.70E-24	86.6
GhNAC127	CotAD_42413	OsNAC8	1.90E-24	88.3
GhNAC128	CotAD_43048	No group	2.70E-24	87.8
GhNAC129	CotAD_43295	No group	7.20E-08	35.2
GhNAC130	CotAD_43532	No group	2.50E-19	72
GhNAC131	CotAD_43826	NAP	7.30E-27	96.1
GhNAC132	CotAD_44666	NAM	2.00E-28	101.1
GhNAC133	CotAD_45354	No group	1.00E-20	76.4
GhNAC134	CotAD_45578	ONAC022	3.50E-25	90.7
GhNAC135	CotAD_45771	ONAC022	2.10E-25	91.4
GhNAC136	CotAD_45977	OsNAC7	3.60E-28	100.3
GhNAC137	CotAD_46230	TERN	6.60E-09	38.5
GhNAC138	CotAD_46279	OsNAC7	1.40E-26	95.2
GhNAC139	CotAD_46383	ATAF	1.70E-27	98.1
GhNAC140	CotAD_46887	TERN	7.10E-23	83.3
GhNAC141	CotAD_47662	NAM	5.40E-29	102.9
GhNAC142	CotAD_48653	ONAC022	2.30E-25	91.3
GhNAC143	CotAD_48894	NAM	2.40E-27	97.6
GhNAC144	CotAD_49372	No group	1.50E-27	98.3
GhNAC145	CotAD_50063	NAP	1.40E-26	95.2
GhNAC146	CotAD_50199	NAC1	2.50E-27	97.6
GhNAC147	CotAD_50352	TIP	3.50E-24	87.5
GhNAC148	CotAD_50950	NAC2	2.20E-27	97.8
GhNAC149	CotAD_51059	NAM	1.10E-28	101.9
GhNAC150	CotAD_51423	ANAC011	4.70E-30	106.3
GhNAC151	CotAD_51810	No group	3.70E-19	71.4
GhNAC152	CotAD_52164	SENU5	1.30E-26	95.2
GhNAC153	CotAD_52166	SENU5	1.40E-26	95.2
GhNAC154	CotAD_52329	NAM	2.00E-28	101.1

GhNAC155	CotAD_52667	No group	2.90E-25	90.9
GhNAC156	CotAD_52769	ONAC003	1.00E-15	60.4
GhNAC157	CotAD_53114	No group	4.80E-25	90.2
GhNAC158	CotAD_53484	No group	1.20E-19	73
GhNAC159	CotAD_53640	ONAC003	1.80E-15	59.6
GhNAC160	CotAD_53818	NAP	2.30E-27	97.7
GhNAC161	CotAD_54844	NAC2	3.00E-27	97.3
GhNAC162	CotAD_55002	NAM	2.00E-29	104.3
GhNAC163	CotAD_55735	No group	4.80E-25	90.2
GhNAC164	CotAD_56163	NAM	2.10E-29	104.2
GhNAC165	CotAD_56167	NAP	2.50E-27	97.6
GhNAC166	CotAD_56445	No group	9.90E-27	95.6
GhNAC167	CotAD_57756	ATAF	3.00E-27	97.3
GhNAC168	CotAD_58138	NAM	7.50E-28	99.2
GhNAC169	CotAD_58753	No group	1.10E-22	82.6
GhNAC170	CotAD_58864	No group	2.50E-19	72
GhNAC171	CotAD_58949	OsNAC7	6.00E-29	102.8
GhNAC172	CotAD_59048	ONAC022	2.70E-25	91
GhNAC173	CotAD_59775	No group	9.50E-20	73.3
GhNAC174	CotAD_59778	NAC2	2.30E-27	97.7
GhNAC175	CotAD_59820	ONAC003	2.50E-13	52.7
GhNAC176	CotAD_59844	ONAC022	4.10E-25	90.5
GhNAC177	CotAD_60285	No group	3.70E-24	87.4
GhNAC178	CotAD_60461	No group	2.10E-20	75.4
GhNAC179	CotAD_60902	OsNAC7	1.40E-26	95.1
GhNAC180	CotAD_61393	TERN	6.90E-23	83.3
GhNAC181	CotAD_61763	NAC1	2.50E-27	97.6
GhNAC182	CotAD_62409	ONAC022	4.00E-25	90.5
GhNAC183	CotAD_63575	No group	1.80E-24	88.4
GhNAC184	CotAD_63894	OsNAC7	2.10E-26	94.6
GhNAC185	CotAD_64058	ONAC003	6.30E-15	57.8
GhNAC186	CotAD_64772	OsNAC7	2.80E-27	97.4
GhNAC187	CotAD_64811	OsNAC7	2.50E-26	94.4
GhNAC188	CotAD_65411	No group	2.40E-17	65.6
GhNAC189	CotAD_65695	ATAF	2.80E-26	94.2
GhNAC190	CotAD_65750	ONAC022	2.10E-25	91.4
GhNAC191	CotAD_65778	ONAC003	1.40E-14	56.7
GhNAC192	CotAD_66079	No group	5.10E-19	70.9
GhNAC193	CotAD_66100	OsNAC7	1.40E-27	98.4
GhNAC194	CotAD_66436	OsNAC7	6.40E-19	70.6
GhNAC195	CotAD_66954	NAP	1.70E-28	101.3
GhNAC196	CotAD_67441	TERN	1.20E-21	79.3
GhNAC197	CotAD_69006	ONAC022	9.80E-18	66.8
GhNAC198	CotAD_70488	ONAC003	1.40E-14	56.7

GhNAC199	CotAD_70745	NAP	1.80E-28	101.2
GhNAC200	CotAD_72992	No group	1.60E-24	88.6
GhNAC201	CotAD_73374	TIP	2.80E-26	94.2
GhNAC202	CotAD_73445	ONAC022	3.80E-25	90.6
GhNAC203	CotAD_73593	NAP	1.20E-26	95.3
GhNAC204	CotAD_73913	NAM	4.10E-29	103.3
GhNAC205	CotAD_73988	OsNAC7	3.30E-18	68.3
GhNAC206	CotAD_74122	ONAC022	2.10E-25	91.4
GhNAC207	CotAD_74448	No group	1.00E-26	95.6
GhNAC208	CotAD_75795	OsNAC7	4.10E-27	96.9
GhNAC209	CotAD_75926	No group	2.90E-24	87.8
GhNAC210	CotAD_76513	OsNAC7	1.20E-25	92.2
GhNAC211	CotAD_76690	OsNAC7	3.30E-26	94

TABLE S2 NAC proteins identified in *A.thaliana*, *T.cacao* and *V.vinifera*.

Name	Subfamily	E-value	Score
AT1G01010	No group	1.80E-19	71.3
AT1G01720	ATAF	1.80E-27	97
AT1G02220	No group	1.10E-19	72
AT1G02230	No group	2.00E-24	87.2
AT1G02250	No group	1.00E-21	78.5
AT1G12260	OsNAC7	1.20E-28	100.8
AT1G19040	No group	1.80E-13	52.1
AT1G25580	ONAC003	1.80E-12	48.9
AT1G26870	ONAC022	1.20E-25	91.1
AT1G28470	ONAC003	1.20E-14	55.8
AT1G32510	ANAC011	2.40E-27	96.5
AT1G32770	OsNAC7	6.60E-27	95.1
AT1G32870	No group	2.10E-26	93.5
AT1G33060	TIP	2.40E-27	96.6
AT1G33280	OsNAC7	4.30E-28	98.9
AT1G34180	No group	7.10E-26	91.8
AT1G34190	No group	1.90E-25	90.5
AT1G52880	NAP	1.70E-27	97
AT1G52890	AtNAC3	1.80E-25	90.6
AT1G54330	ANAC011	3.30E-29	102.5
AT1G56010	No group	1.00E-14	56
AT1G60240	No group	3.10E-07	32.1
AT1G60280	No group	3.80E-15	57.4
AT1G60300	No group	3.90E-16	60.6
AT1G60340	No group	7.50E-14	53.3
AT1G60350	No group	9.60E-17	62.6
AT1G60380	No group	6.90E-15	56.6
AT1G61110	NAP	1.70E-27	97
AT1G62700	OsNAC7	9.30E-29	101.1
AT1G64105	ANAC063	1.70E-10	42.6
AT1G65910	ANAC011	1.30E-29	103.8
AT1G69490	NAP	1.40E-26	94.1
AT1G71930	OsNAC7	8.00E-28	98.1
AT1G76420	No group	1.40E-26	94.1
AT1G77450	ATAF	7.70E-27	94.9
AT1G79580	OsNAC7	3.10E-27	96.2
AT2G02450	No group	3.40E-25	89.7
AT2G17040	No group	1.50E-24	87.6
AT2G18060	OsNAC7	6.40E-29	101.6
AT2G24430	NAM	1.40E-28	100.5
AT2G27300	OsNAC8	2.30E-24	87
AT2G33480	No group	1.40E-27	97.3

AT2G43000	ONAC022	2.40E-25	90.1
AT2G46770	OsNAC7	1.10E-27	97.6
AT3G01600	ONAC003	9.30E-13	49.8
AT3G03200	ANAC011	6.80E-29	101.5
AT3G04060	NAM	1.10E-28	100.8
AT3G04070	NAP	5.70E-28	98.5
AT3G04420	No group	5.80E-22	79.3
AT3G04430	No group	5.90E-18	66.5
AT3G10480	NAC2	1.70E-27	97
AT3G10490	NAC2	6.10E-27	95.2
AT3G10500	No group	1.20E-27	97.5
AT3G12910	ONAC022	8.50E-25	88.4
AT3G12977	No group	1.30E-25	91
AT3G15170	NAM	1.50E-28	100.4
AT3G15500	AtNAC3	1.70E-25	90.6
AT3G15510	NAP	8.50E-28	98
AT3G17730	ANAC011	2.90E-30	105.9
AT3G18400	NAM	1.50E-29	103.6
AT3G29035	NAM	3.90E-30	105.5
AT3G44290	OsNAC8	3.50E-24	86.4
AT3G44350	TERN	2.20E-19	71
AT3G49530	TIP	2.40E-23	83.7
AT3G55210	ANAC063	1.30E-15	58.9
AT3G56520	ANAC063	1.80E-10	42.5
AT3G56530	ANAC063	1.00E-16	62.5
AT3G61910	OsNAC7	9.70E-27	94.6
AT4G01520	No group	4.00E-23	83
AT4G01540	No group	6.40E-22	79.2
AT4G01550	No group	5.60E-25	89
AT4G10350	OsNAC7	6.40E-28	98.4
AT4G17980	ANAC011	7.30E-29	101.4
AT4G27410	AtNAC3	7.60E-26	91.7
AT4G28500	ONAC003	4.50E-15	57.2
AT4G28530	NAC1	1.50E-26	94
AT4G29230	ONAC003	8.60E-16	59.5
AT4G35580	TIP	1.10E-28	100.8
AT4G36160	OsNAC7	5.70E-29	101.8
AT5G04400	No group	3.90E-24	86.3
AT5G04410	No group	9.40E-28	97.8
AT5G07680	NAM	9.10E-30	104.3
AT5G08790	ATAF	9.50E-27	94.6
AT5G09330	No group	1.40E-25	90.9
AT5G13180	SENU5	1.60E-27	97.1
AT5G14000	No group	2.70E-19	70.7

AT5G14490	ONAC003	8.70E-13	49.9
AT5G17260	ANAC011	5.90E-29	101.7
AT5G18270	NAM	4.20E-29	102.2
AT5G18300	ANAC063	2.70E-15	57.9
AT5G22290	OsNAC8	4.70E-25	89.2
AT5G22380	TERN	6.30E-23	82.4
AT5G24590	TIP	7.80E-25	88.5
AT5G39610	NAM	9.10E-30	104.3
AT5G39820	ONAC022	2.00E-25	90.4
AT5G41090	ANAC063	6.50E-11	43.9
AT5G46590	ANAC011	1.30E-29	103.8
AT5G50820	No group	5.60E-18	66.5
AT5G53950	NAM	8.70E-29	101.2
AT5G56620	ONAC003	1.00E-15	59.3
AT5G61430	NAM	9.40E-30	104.3
AT5G62380	OsNAC7	4.40E-28	98.9
AT5G63790	ATAF	5.70E-26	92.1
AT5G64060	No group	3.10E-24	86.6
AT5G64530	No group	7.70E-22	78.9
AT5G66300	OsNAC7	1.30E-26	94.1
Thecc1EG000097	No group	2.90E-25	90.2
Thecc1EG000235	OsNAC7	1.30E-28	100.9
Thecc1EG001442	No group	7.10E-25	88.9
Thecc1EG001450	No group	3.70E-25	89.8
Thecc1EG001514	No group	6.40E-21	76.3
Thecc1EG001595	ATAF	2.40E-27	96.9
Thecc1EG001955	ONAC022	7.60E-25	88.8
Thecc1EG002839	No group	3.20E-20	74
Thecc1EG003998	NAC1	1.30E-27	97.7
Thecc1EG005007	No group	6.50E-19	69.8
Thecc1EG005008	No group	4.10E-22	80.1
Thecc1EG005009	No group	1.50E-21	78.3
Thecc1EG005010	No group	4.70E-22	79.9
Thecc1EG005011	No group	5.30E-18	66.9
Thecc1EG005047	OsNAC7	2.40E-27	96.8
Thecc1EG006499	No group	4.30E-25	89.7
Thecc1EG007543	No group	6.10E-23	82.7
Thecc1EG008229	ONAC003	1.20E-15	59.3
Thecc1EG008477	SENU5	8.80E-27	95.1
Thecc1EG011671	NAP	6.80E-27	95.4
Thecc1EG011718	ONAC022	2.80E-21	77.4
Thecc1EG011869	ONAC003	1.50E-13	52.6
Thecc1EG012350	NAM	1.50E-28	100.7
Thecc1EG014073	NAM	1.70E-29	103.7

Thecc1EG014713	No group	5.10E-26	92.6
Thecc1EG014939	OsNAC7	1.20E-28	101
Thecc1EG015535	TIP	1.70E-27	97.3
Thecc1EG015584	TIP	4.30E-28	99.2
Thecc1EG015621	OsNAC7	1.70E-26	94.1
Thecc1EG015749	ANAC011	2.20E-29	103.4
Thecc1EG016035	OsNAC7	1.10E-28	101.2
Thecc1EG016984	ONAC003	3.90E-13	51.3
Thecc1EG017048	No group	3.40E-23	83.5
Thecc1EG017070	No group	6.40E-23	82.7
Thecc1EG017284	No group	1.70E-20	74.9
Thecc1EG017681	ANAC011	3.80E-30	105.8
Thecc1EG018508	ONAC022	3.50E-25	89.9
Thecc1EG018621	NAM	3.50E-30	105.9
Thecc1EG019937	No group	2.80E-13	51.8
Thecc1EG019938	No group	7.00E-16	60.1
Thecc1EG020121	No group	3.50E-16	61.1
Thecc1EG020125	No group	2.10E-16	61.8
Thecc1EG020127	No group	1.40E-15	59.1
Thecc1EG022059	No group	7.40E-16	60
Thecc1EG022126	NAP	8.30E-28	98.3
Thecc1EG022676	TERN	5.70E-23	82.8
Thecc1EG022690	TERN	1.80E-21	78
Thecc1EG024689	No group	5.50E-25	89.3
Thecc1EG024695	No group	1.70E-23	84.5
Thecc1EG025165	No group	1.10E-12	49.9
Thecc1EG025180	No group	7.40E-13	50.4
Thecc1EG025211	No group	6.50E-13	50.6
Thecc1EG025212	No group	7.80E-13	50.3
Thecc1EG025221	No group	7.00E-13	50.5
Thecc1EG025473	No group	3.10E-13	51.6
Thecc1EG026028	NAM	1.30E-29	104.1
Thecc1EG026030	NAP	4.00E-27	96.1
Thecc1EG027945	No group	8.00E-25	88.8
Thecc1EG028313	No group	1.00E-22	82
Thecc1EG029321	ONAC022	1.10E-24	88.3
Thecc1EG029796	NAP	5.00E-25	89.4
Thecc1EG029798	ONAC003	4.90E-15	57.4
Thecc1EG029931	NAP	3.50E-28	99.5
Thecc1EG030287	No group	2.70E-16	61.5
Thecc1EG031363	NAP	4.80E-28	99.1
Thecc1EG031364	AtNAC3	2.60E-25	90.4
Thecc1EG031619	NAM	1.80E-28	100.4
Thecc1EG032389	ONAC022	2.20E-25	90.6

Thecc1EG032393	ONAC022	1.70E-24	87.8
Thecc1EG032479	No group	3.20E-27	96.5
Thecc1EG033629	ANAC011	7.30E-29	101.7
Thecc1EG033728	OsNAC7	5.80E-28	98.8
Thecc1EG033799	No group	2.10E-17	65
Thecc1EG033990	ANAC063	0.00012	24.1
Thecc1EG034835	No group	2.80E-27	96.6
Thecc1EG035160	ATAF	6.30E-28	98.7
Thecc1EG036034	OsNAC7	1.10E-27	97.9
Thecc1EG036193	No group	7.00E-19	69.7
Thecc1EG036197	No group	1.10E-18	69.1
Thecc1EG036236	No group	9.20E-19	69.3
Thecc1EG036247	No group	2.10E-08	36.2
Thecc1EG036364	OsNAC7	1.50E-28	100.8
Thecc1EG036587	No group	3.10E-27	96.5
Thecc1EG036791	NAM	1.30E-28	100.9
Thecc1EG037747	ONAC003	2.30E-16	61.6
Thecc1EG040737	No group	2.40E-25	90.4
Thecc1EG041055	NAP	2.50E-26	93.6
Thecc1EG041332	OsNAC8	6.30E-25	89.1
Thecc1EG041364	TERN	1.40E-22	81.5
Thecc1EG042655	No group	1.80E-24	87.6
Thecc1EG043185	No group	3.00E-14	54.9
Thecc1EG043195	No group	3.10E-12	48.4
Thecc1EG043199	No group	1.60E-14	55.7
Thecc1EG043357	NAC2	2.10E-27	97.1
Thecc1EG043360	No group	8.10E-28	98.4
Thecc1EG044619	No group	1.40E-08	36.7
Thecc1EG044633	No group	1.30E-13	52.8
Thecc1EG044667	No group	3.00E-12	48.5
Thecc1EG044739	No group	5.80E-13	50.8
Thecc1EG044911	ANAC011	3.40E-30	106
GSVIVT01000940001	SENU5	5.30E-27	95
GSVIVT01001264001	OsNAC7	4.20E-29	101.8
GSVIVT01006485001	NAP	3.60E-28	98.8
GSVIVT01007194001	No group	5.40E-25	88.6
GSVIVT01007982001	NAM	6.00E-30	104.5
GSVIVT01008291001	NAM	7.90E-30	104.1
GSVIVT01008601001	SENU5	4.00E-26	92.2
GSVIVT01008839001	ATAF	1.10E-27	97.2
GSVIVT01011420001	ONAC022	1.40E-25	90.4
GSVIVT01011445001	NAM	3.10E-30	105.4
GSVIVT01011954001	ONAC003	9.90E-14	52.5
GSVIVT01013182001	ANAC011	2.20E-30	105.8

GSVIVT01013419001	No group	5.50E-28	98.2
GSVIVT01013671001	No group	1.30E-27	97
GSVIVT01014287001	NAM	2.80E-28	99.1
GSVIVT01014403001	AtNAC3	1.60E-26	93.5
GSVIVT01014405001	NAP	2.60E-28	99.2
GSVIVT01015274001	ONAC003	1.90E-16	61.2
GSVIVT01016175001	No group	1.80E-27	96.6
GSVIVT01016176001	NAC2	6.10E-27	94.8
GSVIVT01018623001	OsNAC7	4.60E-29	101.6
GSVIVT01018809001	OsNAC7	1.70E-29	103
GSVIVT01019670001	OsNAC7	2.60E-27	96
GSVIVT01019702001	TIP	1.30E-26	93.8
GSVIVT01019952001	NAP	3.00E-27	95.8
GSVIVT01019993001	ONAC022	9.20E-26	91.1
GSVIVT01020384001	ONAC022	3.20E-25	89.3
GSVIVT01020387001	ONAC022	3.50E-26	92.4
GSVIVT01020388001	ONAC022	4.50E-26	92.1
GSVIVT01020389001	ONAC022	7.20E-26	91.4
GSVIVT01020394001	ONAC022	4.70E-26	92
GSVIVT01020478001	No group	1.70E-26	93.4
GSVIVT01020609001	ONAC003	1.40E-15	58.4
GSVIVT01020834001	ONAC022	1.90E-25	90
GSVIVT01021120001	ONAC003	1.50E-15	58.3
GSVIVT01022354001	ATAF	2.10E-27	96.3
GSVIVT01023123001	No group	1.90E-25	90.1
GSVIVT01023921001	No group	1.00E-24	87.7
GSVIVT01025165001	NAP	3.70E-26	92.3
GSVIVT01025244001	No group	1.50E-23	83.9
GSVIVT01025515001	No group	1.90E-27	96.4
GSVIVT01025657001	NAP	2.30E-27	96.2
GSVIVT01026055001	TERN	5.40E-24	85.4
GSVIVT01026468001	No group	6.30E-22	78.8
GSVIVT01026495001	No group	2.40E-25	89.7
GSVIVT01027431001	OsNAC7	1.40E-27	96.9
GSVIVT01027470001	No group	1.40E-25	90.5
GSVIVT01027472001	No group	1.80E-23	83.7
GSVIVT01027473001	No group	9.80E-21	74.9
GSVIVT01027475001	No group	4.20E-47	159.5
GSVIVT01027477001	No group	2.80E-27	95.9
GSVIVT01028354001	NAC1	5.80E-28	98.1
GSVIVT01029392001	ANAC011	3.00E-30	105.4
GSVIVT01029709001	No group	1.80E-23	83.7
GSVIVT01032388001	ONAC003	8.50E-13	49.5
GSVIVT01033032001	No group	3.40E-25	89.3

GSVIVT01033372001	NAP	4.10E-27	95.4
GSVIVT01033374001	NAM	1.20E-29	103.5
GSVIVT01033886001	TERN	1.10E-11	45.9
GSVIVT01034485001	OsNAC7	2.30E-28	99.4
GSVIVT01035214001	ANAC011	3.40E-30	105.3
GSVIVT01035554001	NAM	3.30E-28	98.9
GSVIVT01036071001	OsNAC8	1.10E-25	90.8
GSVIVT01036091001	TERN	2.10E-25	89.9
GSVIVT01036682001	OsNAC7	6.50E-28	98
GSVIVT01036711001	ANAC011	2.70E-29	102.4
GSVIVT01037113001	OsNAC7	3.40E-28	98.8
GSVIVT01038666001	TIP	7.20E-27	94.6

TABLE S3 List of the orthologous groups of GaNAC, GrNAC and GhNAC through OrthoMCL clustering.

Name	Subfamily	Orthomcl_group	Seq_id_of_best_hit	E value_mantissa	E value_exponent	Percent_identity	Percent_match
GrNAC1	ATAF	OG5_135169	rcom|29648.m002012	1	-141	79	99
GrNAC2	ONAC003	OG5_213021	rcom|29738.m001046	1	-171	68	100
GrNAC3	ONAC022	OG5_190119	rcom|30169.m006343	1	-101	63	99
GrNAC4	ONAC022	OG5_190119	rcom|30169.m006343	6	-79	51	94
GrNAC5	ONAC022	OG5_177548	rcom|29032.m000021	1	-143	66	100
GrNAC6	SENU5	OG5_212763	rcom|30128.m009035	1	-113	79	100
GrNAC7	No group	NO_GROUP	rcom|29686.m000865	7	-25	36	56
GrNAC8	NAM	OG5_150285	rcom|27950.m000105	1	-125	70	100
GrNAC9	OsNAC7	OG5_170479	rcom|30068.m002591	1	-172	73	100
GrNAC10	AtNAC3	OG5_135169	rcom|28219.m000090	1	-131	70	100
GrNAC11	NAP	OG5_135169	rcom|27961.m000091	1	-153	76	100
GrNAC12	No group	OG5_167608	rcom|30076.m004477	2	-29	49	66
GrNAC13	NAM	OG5_164656	rcom|30138.m004055	1	-159	74	100
GrNAC14	No group	OG5_164225	rcom|29650.m000275	2	-69	59	100
GrNAC15	No group	NO_GROUP	rcom|29686.m000865	2	-31	48	72
GrNAC16	NAP	OG5_177391	rcom|29950.m001173	1	-114	62	95
GrNAC17	No group	NO_GROUP	rcom|28011.m000080	3	-46	46	78
GrNAC18	No group	NO_GROUP	rcom|28011.m000080	4	-35	54	76
GrNAC19	OsNAC7	OG5_212584	rcom|29382.m000084	1	-131	65	95
GrNAC20	ANAC011	OG5_139552	rcom|30171.m000407	1	-179	53	100
GrNAC21	NAC2	OG5_160086	atha|NP_850554	1	-127	55	96
GrNAC22	No group	OG5_164698	rcom|30063.m001417	1	-139	67	100
GrNAC23	NAP	OG5_213067	rcom|29813.m001519	1	-114	70	98
GrNAC24	OsNAC7	OG5_140455	rcom|30147.m014211	1	-140	70	100

GrNAC25	No group	OG5_178238	rcom|29797.m000353	1	-104	65	100
GrNAC26	NAC2	OG5_160086	atha|NP_850554	1	-124	54	100
GrNAC27	OsNAC7	OG5_140455	rcom|28200.m000189	1	-166	80	100
GrNAC28	OsNAC7	OG5_140455	rcom|28200.m000189	1	-163	77	100
GrNAC29	ANAC011	OG5_190411	rcom|29827.m002555	1	-116	65	97
GrNAC30	ONAC022	OG5_177548	rcom|29032.m000021	2	-94	48	99
GrNAC31	ANAC011	OG5_139552	rcom|30172.m000206	4	-87	82	100
GrNAC32	No group	NO_GROUP	rcom|30131.m007019	4	-54	58	99
GrNAC33	No group	NO_GROUP	rcom|29830.m001415	4	-67	69	92
GrNAC34	ONAC003	OG5_177766	rcom|29917.m002016	1	-135	63	100
GrNAC35	No group	OG5_135169	osat|NP_001060017	1	-34	48	86
GrNAC36	TIP	OG5_160072	rcom|30068.m002619	1	-131	44	98
GrNAC37	TIP	OG5_190295	rcom|29764.m000758	1	-102	44	88
GrNAC38	NAM	OG5_150285	rcom|27950.m000105	1	-120	70	100
GrNAC39	No group	OG5_167608	rcom|30076.m004487	3	-32	40	50
GrNAC40	NAC1	OG5_170224	osat|NP_001053312	2	-78	58	92
GrNAC41	SENU5	OG5_212763	rcom|30128.m009035	1	-116	80	100
GrNAC42	ANAC011	OG5_139552	rcom|29770.m000336	1	-123	65	100
GrNAC43	OsNAC7	OG5_160167	rcom|29725.m000235	1	-137	73	100
GrNAC44	ONAC022	OG5_190119	rcom|30169.m006343	5	-76	52	98
GrNAC45	ONAC022	OG5_190119	rcom|30169.m006343	2	-78	53	93
GrNAC46	ONAC022	OG5_190119	rcom|30169.m006343	3	-82	55	98
GrNAC47	OsNAC7	OG5_170479	rcom|30068.m002591	1	-141	64	95
GrNAC48	TERN	OG5_156371	atha|NP_001118771	8	-71	56	99
GrNAC49	OsNAC8	OG5_212233	rcom|30055.m001612	1	-123	63	93
GrNAC50	OsNAC7	OG5_170479	rcom|27964.m000369	1	-144	74	99

GrNAC51	TIP	OG5_160072	rcom|30068.m002619	1	-122	44	91
GrNAC52	OsNAC7	OG5_170479	rcom|30068.m002591	1	-152	67	100
GrNAC53	TIP	OG5_190295	rcom|29764.m000758	5	-92	55	59
GrNAC54	No group	NO_GROUP	rcom|29686.m000865	8	-20	31	67
GrNAC55	No group	OG5_135169	osat|NP_001044617	1	-38	39	65
GrNAC56	No group	OG5_178238	rcom|29797.m000353	6	-98	62	97
GrNAC57	No group	OG5_178238	rcom|29797.m000353	1	-104	66	95
GrNAC58	ANAC011	OG5_139552	rcom|29770.m000336	1	-132	70	98
GrNAC59	NAP	OG5_135169	rcom|27961.m000091	1	-144	72	100
GrNAC60	AtNAC3	OG5_135169	rcom|28219.m000090	1	-136	73	100
GrNAC61	OsNAC7	OG5_212126	rcom|29728.m000815	1	-104	59	99
GrNAC62	No group	OG5_178154	rcom|29683.m000467	1	-48	48	97
GrNAC63	ATAF	NO_GROUP	rcom|30193.m000709	3	-97	67	78
GrNAC64	No group	OG5_160025	atha|NP_564440	1	-121	43	98
GrNAC65	NAP	OG5_245102	rcom|30078.m002348	2	-63	66	54
GrNAC66	ONAC022	OG5_190119	rcom|30169.m006343	1	-89	57	95
GrNAC67	No group	OG5_213067	rcom|29813.m001519	4	-44	49	57
GrNAC68	No group	OG5_160286	atha|NP_195080	7	-57	52	52
GrNAC69	No group	OG5_213067	rcom|29813.m001519	2	-47	49	62
GrNAC70	No group	OG5_213067	rcom|29813.m001519	3	-47	49	62
GrNAC71	TERN	OG5_244818	rcom|30032.m000479	6	-74	72	78
GrNAC72	NAC1	OG5_170224	osat|NP_001053312	9	-85	57	97
GrNAC73	TIP	OG5_190295	rcom|29764.m000758	1	-102	44	87
GrNAC74	ANAC011	OG5_139552	rcom|30172.m000206	1	-110	79	99
GrNAC75	No group	NO_GROUP	rcom|30147.m014441	1	-14	28	61
GrNAC76	No group	NO_GROUP	rcom|30076.m004475	5	-18	31	55

GrNAC77	No group	NO_GROUP	rcom|30076.m004475	1	-17	36	67
GrNAC78	No group	OG5_135169	ppat|e_gw1.164.18.1	7	-14	29	92
GrNAC79	No group	OG5_177548	rcom|29813.m001479	7	-12	27	53
GrNAC80	No group	OG5_160025	atha|NP_564440	1	-123	43	98
GrNAC81	NAM	OG5_164511	rcom|29950.m001171	1	-111	57	100
GrNAC82	ONAC022	OG5_190119	rcom|30169.m006343	7	-71	47	98
GrNAC83	OsNAC7	OG5_140455	rcom|28200.m000189	1	-172	83	100
GrNAC84	ANAC011	OG5_190411	rcom|29827.m002555	1	-123	70	96
GrNAC85	No group	NO_GROUP	rcom|30169.m006458	1	-55	56	63
GrNAC86	No group	OG5_139552	rcom|28872.m000248	1	-163	52	99
GrNAC87	NAC2	OG5_160086	atha|NP_974272	1	-121	54	99
GrNAC88	NAP	OG5_245102	rcom|30078.m002348	1	-100	69	87
GrNAC89	NAC1	OG5_170224	osat|NP_001053312	3	-80	58	96
GrNAC90	TERN	OG5_156371	atha|NP_001118771	5	-67	56	99
GrNAC91	OsNAC7	OG5_170479	rcom|27964.m000369	1	-141	74	99
GrNAC92	No group	OG5_160072	atha|NP_567986	3	-37	33	57
GrNAC93	No group	OG5_189953	rcom|30170.m014236	1	-125	65	97
GrNAC94	No group	OG5_178154	rcom|29683.m000467	2	-81	78	100
GrNAC95	No group	OG5_178154	rcom|29683.m000467	1	-71	71	98
GrNAC96	No group	NO_GROUP	rcom|29686.m000865	4	-23	36	61
GrNAC97	NAC1	OG5_170224	osat|NP_001053312	2	-81	55	96
GrNAC98	No group	OG5_190295	atha|NP_197847	2	-32	38	51
GrNAC99	OsNAC7	OG5_170479	rcom|30068.m002591	1	-157	69	100
GrNAC100	NAP	OG5_245102	rcom|30111.m000742	6	-65	70	60
GrNAC101	ONAC022	OG5_190119	osat|NP_001051438	1	-49	69	96
GrNAC102	OsNAC8	OG5_212233	rcom|30055.m001612	1	-119	60	91

GrNAC103	OsNAC7	OG5_140455	rcom|30147.m014211	1	-123	67	98
GrNAC104	No group	OG5_164698	rcom|30063.m001417	1	-136	71	91
GrNAC105	NAM	OG5_164656	rcom|30138.m004055	1	-107	56	100
GrNAC106	ONAC003	OG5_137520	rcom|29917.m002005	0	-181	61	98
GrNAC107	No group	OG5_156359	atha|NP_001154700	8	-78	50	86
GrNAC108	OsNAC7	OG5_170428	rcom|28623.m000400	1	-122	65	100
GrNAC109	No group	NO_GROUP	rcom|30147.m014441	1	-55	69	90
GrNAC110	NAM	OG5_164656	rcom|30138.m004055	1	-128	65	100
GrNAC111	ANAC011	OG5_190411	rcom|29827.m002555	1	-102	62	98
GrNAC112	ONAC022	OG5_177548	rcom|29813.m001479	1	-126	62	100
GrNAC113	ONAC003	OG5_170647	rcom|30190.m010767	1	-129	71	100
GrNAC114	NAP	OG5_135169	rcom|29851.m002359	1	-130	69	99
GrNAC115	No group	OG5_189953	rcom|30170.m014236	1	-137	64	100
GrNAC116	ANAC063	OG5_243120	rcom|30170.m013995	3	-46	40	99
GrNAC117	OsNAC7	OG5_170428	rcom|28623.m000400	1	-131	70	99
GrNAC118	ANAC011	OG5_139552	rcom|30171.m000407	1	-179	52	99
GrNAC119	ONAC003	OG5_170647	rcom|29983.m003125	1	-146	87	100
GrNAC120	No group	NO_GROUP	rcom|30131.m007019	1	-70	57	93
GrNAC121	TERN	OG5_244818	rcom|30032.m000479	1	-81	60	96
GrNAC122	OsNAC7	OG5_170479	rcom|27964.m000369	1	-147	77	99
GrNAC123	No group	OG5_167608	rcom|30076.m004477	4	-29	37	86
GrNAC124	No group	OG5_167608	rcom|30076.m004477	3	-37	44	84
GrNAC125	No group	NO_GROUP	rcom|30169.m006458	3	-62	58	55
GrNAC126	ONAC003	OG5_190453	rcom|29912.m005354	0	-181	76	100
GrNAC127	ANAC011	OG5_190411	rcom|29827.m002555	1	-129	72	96
GrNAC128	NAP	OG5_213067	rcom|29813.m001519	1	-114	73	96

GrNAC129	ONAC022	OG5_177548	rcom|29813.m001479	1	-136	65	100
GrNAC130	No group	OG5_178154	rcom|29683.m000467	1	-56	75	71
GrNAC131	ONAC003	OG5_190453	rcom|29912.m005354	0	-181	84	96
GrNAC132	ATAF	OG5_135169	rcom|29648.m002012	1	-128	75	98
GrNAC133	NAM	OG5_150285	rcom|28226.m000851	1	-130	72	100
GrNAC134	NAP	OG5_135169	rcom|27961.m000091	1	-140	72	100
GrNAC135	NAM	OG5_150285	rcom|27950.m000105	1	-123	69	100
GrNAC136	NAM	OG5_164511	rcom|29950.m001171	1	-121	62	100
GrNAC137	NAP	OG5_177391	rcom|29950.m001173	1	-133	69	99
GrNAC138	NAM	OG5_190207	rcom|29974.m000235	1	-115	69	100
GrNAC139	OsNAC7	OG5_170428	rcom|28623.m000400	1	-137	70	99
GrNAC140	ONAC022	OG5_190119	rcom|30169.m006343	1	-80	55	97
GrNAC141	ONAC003	OG5_170647	rcom|30190.m010767	1	-124	72	99
GrNAC142	NAP	OG5_135169	rcom|29851.m002359	1	-104	74	93
GaNAC1	No group	OG5_139552	atha|NP_188400	6	-35	43	65
GaNAC2	No group	OG5_178238	rcom|29797.m000353	1	-97	63	97
GaNAC3	No group	OG5_178238	rcom|29797.m000353	1	-104	66	98
GaNAC4	No group	OG5_178154	rcom|29683.m000467	6	-72	71	98
GaNAC5	No group	OG5_139552	atha|NP_176766	1	-6	29	72
GaNAC6	No group	NO_GROUP	rcom|29830.m001415	1	-67	69	93
GaNAC7	OsNAC7	OG5_170428	rcom|28623.m000400	1	-103	59	100
GaNAC8	No group	NO_GROUP	rcom|30169.m006458	3	-62	58	55
GaNAC9	No group	OG5_212763	rcom|30128.m009035	1	-63	55	96
GaNAC10	OsNAC7	OG5_170428	rcom|28623.m000400	8	-78	89	97
GaNAC11	ANAC011	OG5_139552	rcom|29770.m000336	1	-132	68	98
GaNAC12	TIP	OG5_190295	rcom|29764.m000758	1	-80	77	93

GaNAC13	TIP	OG5_190295	rcom|29764.m000758	2	-90	54	61
GaNAC14	ANAC011	OG5_139552	rcom|30172.m000206	1	-109	79	99
GaNAC15	NAC1	OG5_170224	osat|NP_001053312	3	-80	56	97
GaNAC16	OsNAC7	OG5_140455	rcom|30147.m014211	1	-141	70	100
GaNAC17	NAP	OG5_245102	rcom|30111.m000742	2	-64	69	60
GaNAC18	No group	OG5_190411	atha|NP_193532	5	-13	31	50
GaNAC19	No group	OG5_140951	rcom|29657.m000482	1	-12	32	55
GaNAC20	ONAC003	OG5_170647	rcom|29983.m003125	1	-145	86	100
GaNAC21	No group	OG5_160025	atha|NP_564440	1	-126	44	99
GaNAC22	No group	OG5_189953	rcom|30170.m014236	1	-136	64	100
GaNAC23	No group	NO_GROUP	rcom|29686.m000865	8	-31	47	72
GaNAC24	No group	OG5_190295	atha|NP_197847	9	-31	42	85
GaNAC25	ONAC022	OG5_190119	osat|NP_001051438	2	-60	73	74
GaNAC26	No group	OG5_139552	osat|NP_001062518	1	-20	33	79
GaNAC27	OsNAC7	OG5_212584	atha|NP_192773	4	-81	90	90
GaNAC28	ANAC011	OG5_139552	rcom|29770.m000336	1	-122	65	100
GaNAC29	OsNAC7	OG5_160167	rcom|29725.m000235	1	-136	73	100
GaNAC30	NAC2	OG5_160086	atha|NP_850554	1	-125	53	96
GaNAC31	NAP	OG5_213067	rcom|29813.m001519	1	-115	70	98
GaNAC32	NAM	OG5_150285	rcom|27950.m000105	1	-120	72	97
GaNAC33	AtNAC3	OG5_135169	rcom|28219.m000090	1	-132	70	100
GaNAC34	NAP	OG5_135169	rcom|27961.m000091	1	-152	76	100
GaNAC35	ANAC063	OG5_243120	rcom|30170.m013995	6	-50	44	88
GaNAC36	OsNAC7	OG5_140455	rcom|28200.m000189	1	-171	82	100
GaNAC37	No group	NO_GROUP	rcom|30131.m007019	9	-73	55	93
GaNAC38	ANAC011	OG5_190411	rcom|29827.m002555	1	-120	69	88

GaNAC39	TIP	OG5_190295	rcom|29764.m000758	1	-104	44	92
GaNAC40	OsNAC7	OG5_170479	rcom|30068.m002591	1	-141	64	99
GaNAC41	No group	NO_GROUP	rcom|30170.m013849	4	-74	55	97
GaNAC42	No group	OG5_160025	atha|NP_564440	1	-124	45	98
GaNAC43	ANAC011	OG5_190411	rcom|29827.m002555	1	-101	62	98
GaNAC44	ONAC003	OG5_190453	rcom|29912.m005354	0	-181	75	98
GaNAC45	No group	OG5_156359	atha|NP_001154700	1	-77	49	86
GaNAC46	TERN	OG5_156371	rcom|30055.m001588	1	-78	62	98
GaNAC47	SENU5	OG5_212763	rcom|30128.m009035	1	-114	79	100
GaNAC48	SENU5	OG5_212763	rcom|30128.m009035	1	-112	79	100
GaNAC49	TERN	OG5_244818	rcom|30032.m000479	3	-75	67	89
GaNAC50	ONAC003	OG5_170647	rcom|30190.m010767	1	-130	71	100
GaNAC51	OsNAC7	OG5_170428	rcom|28623.m000400	4	-77	88	98
GaNAC52	ANAC011	OG5_139552	rcom|30171.m000407	1	-171	55	93
GaNAC53	No group	OG5_189953	rcom|30170.m014236	1	-124	65	97
GaNAC54	ANAC011	OG5_139552	rcom|30172.m000206	1	-118	83	100
GaNAC55	ANAC011	OG5_190411	rcom|29827.m002555	1	-128	71	89
GaNAC56	OsNAC7	OG5_170479	rcom|30068.m002591	1	-152	68	100
GaNAC57	NAM	OG5_164656	rcom|30138.m004055	1	-158	73	100
GaNAC58	No group	OG5_213067	rcom|29813.m001519	1	-40	50	56
GaNAC59	NAP	OG5_135169	rcom|27961.m000091	1	-143	72	100
GaNAC60	AtNAC3	OG5_135169	rcom|28219.m000090	1	-140	74	100
GaNAC61	ANAC063	OG5_243120	rcom|30170.m013995	4	-47	39	99
GaNAC62	OsNAC8	OG5_212233	rcom|30055.m001612	1	-115	59	90
GaNAC63	No group	OG5_160072	atha|NP_001119122	2	-38	33	58
GaNAC64	No group	OG5_167608	rcom|30076.m004487	8	-32	37	50

GaNAC65	NAM	OG5_164656	rcom|30138.m004055	1	-107	56	100
GaNAC66	OsNAC7	OG5_170479	rcom|27964.m000369	1	-148	78	99
GaNAC67	No group	OG5_167608	rcom|30076.m004477	1	-28	38	79
GaNAC68	No group	OG5_167608	rcom|30076.m004477	1	-36	44	84
GaNAC69	No group	OG5_178154	rcom|29683.m000467	6	-79	73	99
GaNAC70	ONAC022	OG5_190119	rcom|30169.m006343	3	-90	55	100
GaNAC71	ATAF	OG5_135169	rcom|29648.m002012	1	-141	79	99
GaNAC72	NAM	OG5_150285	rcom|27950.m000105	1	-124	70	100
GaNAC73	NAP	OG5_135169	rcom|27961.m000091	1	-140	72	100
GaNAC74	OsNAC7	OG5_140455	rcom|28200.m000189	1	-164	78	100
GaNAC75	NAM	OG5_150285	rcom|27950.m000105	1	-120	69	100
GaNAC76	TIP	OG5_160072	rcom|30068.m002619	1	-124	44	91
GaNAC77	ONAC003	OG5_190453	rcom|29912.m005354	0	-181	82	100
GaNAC78	NAM	OG5_164511	rcom|29950.m001171	1	-123	62	100
GaNAC79	NAP	OG5_177391	rcom|29950.m001173	1	-131	69	99
GaNAC80	OsNAC7	OG5_140455	rcom|28200.m000189	1	-166	81	100
GaNAC81	OsNAC7	OG5_170428	rcom|28623.m000400	3	-82	97	98
GaNAC82	ONAC022	OG5_177548	rcom|29032.m000021	2	-94	48	99
GaNAC83	ONAC003	OG5_177766	rcom|29917.m002016	1	-116	59	91
GaNAC84	NAC1	OG5_170224	osat|NP_001053312	2	-82	55	96
GaNAC85	No group	OG5_164698	rcom|30063.m001417	1	-136	71	93
GaNAC86	No group	NO_GROUP	rcom|30131.m007019	2	-69	57	93
GaNAC87	ANAC011	OG5_190411	rcom|29827.m002555	1	-115	65	98
GaNAC88	OsNAC7	OG5_140455	rcom|30147.m014211	1	-121	66	98
GaNAC89	NAP	OG5_177391	rcom|29950.m001173	1	-113	61	95
GaNAC90	ONAC022	OG5_190119	rcom|30169.m006343	2	-77	53	98

GaNAC91	ONAC022	OG5_190119	rcom|30169.m006343	2	-77	52	95
GaNAC92	ONAC022	OG5_190119	rcom|30169.m006343	6	-82	55	98
GaNAC93	SENU5	OG5_212763	rcom|30128.m009035	1	-116	80	100
GaNAC94	No group	OG5_164225	rcom|29650.m000275	2	-82	71	100
GaNAC95	No group	OG5_135169	osat|NP_001060017	2	-47	44	57
GaNAC96	No group	OG5_135169	osat|NP_001060017	2	-46	43	54
GaNAC97	No group	OG5_213067	rcom|29813.m001519	4	-41	46	76
GaNAC98	ATAF	NO_GROUP	rcom|30193.m000709	1	-109	68	100
GaNAC99	OsNAC8	OG5_212233	rcom|30055.m001612	1	-120	62	94
GaNAC100	TERN	OG5_156371	atha|NP_001118771	6	-71	55	98
GaNAC101	OsNAC7	OG5_170479	rcom|27964.m000369	1	-144	74	99
GaNAC102	No group	OG5_178154	rcom|29683.m000467	3	-81	78	100
GaNAC103	No group	NO_GROUP	rcom|29686.m000865	4	-26	35	62
GaNAC104	No group	NO_GROUP	rcom|29686.m000865	6	-26	36	57
GaNAC105	No group	NO_GROUP	rcom|29686.m000865	2	-23	36	60
GaNAC106	NAC1	OG5_170224	osat|NP_001053312	2	-80	58	96
GaNAC107	NAP	OG5_213067	rcom|29813.m001519	1	-117	72	100
GaNAC108	No group	NO_GROUP	rcom|30131.m007019	1	-53	49	95
GaNAC109	No group	NO_GROUP	rcom|30169.m006458	3	-56	58	63
GaNAC110	NAC2	OG5_160086	atha|NP_974272	1	-121	54	99
GaNAC111	No group	OG5_139552	rcom|28872.m000248	1	-153	51	99
GaNAC112	NAC1	OG5_170224	osat|NP_001053312	1	-82	57	97
GaNAC113	ONAC022	OG5_190119	rcom|30169.m006343	7	-98	61	99
GaNAC114	No group	OG5_164225	rcom|29650.m000275	3	-94	60	97
GaNAC115	ONAC022	OG5_177548	rcom|29032.m000021	1	-145	65	100
GaNAC116	OsNAC7	OG5_170479	rcom|30068.m002591	1	-160	70	100

GaNAC117	No group	OG5_178238	rcom|29797.m000353	1	-104	66	96
GaNAC118	NAC2	OG5_160086	atha|NP_850554	1	-121	52	99
GaNAC119	NAP	OG5_245102	rcom|30078.m002348	1	-97	68	88
GaNAC120	ONAC022	OG5_190119	rcom|30169.m006343	2	-79	52	94
GaNAC121	ATAF	OG5_135169	rcom|29648.m002012	1	-127	74	98
GaNAC122	TIP	OG5_190295	rcom|29764.m000758	1	-103	45	87
GaNAC123	ONAC003	OG5_213021	rcom|29738.m001046	1	-169	67	100
GaNAC124	No group	NO_GROUP	rcom|29686.m000865	2	-24	36	54
GaNAC125	NAM	OG5_164511	rcom|29950.m001171	1	-112	57	100
GaNAC126	NAP	OG5_245102	rcom|30078.m002348	1	-63	66	54
GaNAC127	TIP	OG5_160072	rcom|30068.m002619	1	-131	44	98
GaNAC128	NAM	OG5_164656	rcom|30138.m004055	1	-128	65	100
GaNAC129	ONAC003	OG5_177766	rcom|29917.m002016	1	-137	65	94
GaNAC130	OsNAC7	OG5_170479	rcom|27964.m000369	1	-138	73	99
GaNAC131	NAM	OG5_150285	rcom|28226.m000851	1	-132	73	100
GaNAC132	OsNAC7	OG5_212126	rcom|29728.m000815	3	-96	56	99
GaNAC133	TERN	OG5_244818	rcom|30032.m000479	5	-79	59	96
GaNAC134	ONAC022	OG5_177548	rcom|29813.m001479	1	-134	65	100
GaNAC135	NAP	OG5_135169	rcom|29851.m002359	1	-133	69	99
GaNAC136	ONAC022	OG5_190119	rcom|30169.m006343	7	-81	56	95
GaNAC137	ONAC003	OG5_170647	rcom|30190.m010767	1	-125	73	99
GaNAC138	No group	OG5_167608	rcom|30076.m004477	2	-29	49	66
GaNAC139	NAP	OG5_135169	rcom|29851.m002359	1	-124	66	96
GaNAC140	ONAC022	OG5_177548	rcom|29813.m001479	1	-120	61	100
GaNAC141	OsNAC7	OG5_170479	rcom|30068.m002591	1	-171	73	100
GaNAC142	No group	OG5_164698	rcom|30063.m001417	1	-136	72	100

GhNAC1	NAP	OG5_245102	rcom|30111.m000742	2	-64	69	60
GhNAC2	OsNAC7	OG5_140455	rcom|30147.m014211	1	-123	67	98
GhNAC3	TIP	OG5_160072	rcom|30068.m002619	1	-131	45	92
GhNAC4	SENU5	OG5_212763	rcom|30128.m009035	1	-114	80	100
GhNAC5	OsNAC7	OG5_170428	rcom|28623.m000400	1	-103	59	100
GhNAC6	TIP	OG5_190295	rcom|29764.m000758	2	-91	55	62
GhNAC7	No group	OG5_178238	rcom|29797.m000353	1	-104	66	95
GhNAC8	ANAC011	OG5_139552	rcom|30172.m000206	1	-115	75	100
GhNAC9	No group	NO_GROUP	rcom|30170.m013849	6	-64	60	73
GhNAC10	OsNAC7	OG5_170428	rcom|28623.m000400	8	-78	90	95
GhNAC11	No group	NO_GROUP	rcom|30147.m014441	1	-54	69	90
GhNAC12	NAM	OG5_164656	rcom|30138.m004055	1	-129	65	100
GhNAC13	ANAC011	OG5_190411	rcom|29827.m002555	1	-102	62	98
GhNAC14	OsNAC7	OG5_212584	rcom|29382.m000084	1	-131	65	95
GhNAC15	ANAC011	OG5_139552	rcom|29770.m000336	1	-122	65	100
GhNAC16	OsNAC7	OG5_160167	rcom|29725.m000235	1	-132	72	100
GhNAC17	ONAC022	OG5_190119	rcom|30169.m006343	2	-76	52	98
GhNAC18	ONAC022	OG5_190119	rcom|30169.m006343	4	-77	51	95
GhNAC19	ONAC022	OG5_190119	rcom|30169.m006343	4	-82	55	98
GhNAC20	NAP	OG5_177391	rcom|29950.m001173	1	-114	62	95
GhNAC21	No group	OG5_167608	rcom|30076.m004487	3	-32	40	50
GhNAC22	No group	OG5_156359	atha|NP_001154700	1	-77	50	86
GhNAC23	NAM	OG5_164656	rcom|30138.m004055	1	-107	56	100
GhNAC24	ONAC022	OG5_190119	osat|NP_001051438	1	-60	73	76
GhNAC25	No group	OG5_160025	atha|NP_564440	1	-121	43	98
GhNAC26	NAP	OG5_135169	rcom|27961.m000091	1	-140	72	100

GhNAC27	NAM	OG5_150285	rcom|27950.m000105	1	-122	69	100
GhNAC28	No group	OG5_160025	atha|NP_564440	1	-114	45	89
GhNAC29	No group	OG5_160025	atha|NP_564440	9	-98	52	97
GhNAC30	NAC2	OG5_160086	atha|NP_001154602	1	-112	45	99
GhNAC31	No group	OG5_178238	rcom|29797.m000353	6	-83	62	97
GhNAC32	No group	OG5_213067	rcom|29813.m001519	2	-38	44	68
GhNAC33	ANAC011	OG5_139552	rcom|30172.m000206	1	-109	78	99
GhNAC34	ANAC011	OG5_139552	rcom|30171.m000407	0	-181	53	100
GhNAC35	No group	OG5_156359	atha|NP_001154700	9	-78	49	86
GhNAC36	ONAC003	OG5_177766	rcom|29917.m002016	1	-99	65	83
GhNAC37	NAM	OG5_164656	rcom|30138.m004055	1	-107	55	100
GhNAC38	NAC1	OG5_170224	osat|NP_001053312	4	-82	55	96
GhNAC39	TERN	OG5_244818	rcom|30032.m000479	1	-74	64	94
GhNAC40	No group	OG5_178154	rcom|29683.m000467	2	-71	71	98
GhNAC41	OsNAC8	OG5_212233	rcom|30055.m001612	1	-119	60	91
GhNAC42	ONAC003	OG5_170647	rcom|29983.m003125	1	-146	87	100
GhNAC43	No group	OG5_178154	rcom|29683.m000467	2	-56	72	98
GhNAC44	NAP	OG5_213067	rcom|29813.m001519	1	-114	70	98
GhNAC45	ONAC003	OG5_177766	rcom|29917.m002016	1	-135	63	94
GhNAC46	No group	NO_GROUP	rcom|30169.m006458	3	-61	58	55
GhNAC47	ONAC022	OG5_190119	rcom|30169.m006343	4	-81	55	98
GhNAC48	ONAC022	OG5_190119	rcom|30169.m006343	2	-78	54	95
GhNAC49	ONAC022	OG5_190119	rcom|30169.m006343	2	-76	53	98
GhNAC50	OsNAC7	OG5_160167	rcom|29725.m000235	1	-137	73	100
GhNAC51	ANAC011	OG5_139552	rcom|29770.m000336	1	-122	65	100
GhNAC52	TIP	OG5_160072	rcom|30068.m002619	1	-121	46	83

GhNAC53	OsNAC7	OG5_170479	rcom|30068.m002591	1	-150	67	100
GhNAC54	No group	NO_GROUP	rcom|30131.m007019	2	-72	54	98
GhNAC55	No group	OG5_164698	rcom|30063.m001417	1	-139	67	100
GhNAC56	NAP	OG5_213067	rcom|29813.m001519	1	-115	70	98
GhNAC57	ANAC011	OG5_190411	rcom|29827.m002555	1	-128	71	96
GhNAC58	NAM	OG5_150285	rcom|27950.m000105	1	-122	69	100
GhNAC59	No group	OG5_213067	rcom|29813.m001519	2	-39	45	82
GhNAC60	ANAC011	OG5_190411	rcom|29827.m002555	1	-110	70	100
GhNAC61	ANAC011	OG5_139552	rcom|30172.m000206	3	-78	92	97
GhNAC62	OsNAC7	OG5_170479	rcom|30068.m002591	1	-141	64	98
GhNAC63	No group	NO_GROUP	rcom|30169.m006458	2	-62	57	56
GhNAC64	NAP	OG5_245102	rcom|30078.m002348	1	-63	66	54
GhNAC65	NAM	OG5_164511	rcom|29950.m001171	1	-121	62	100
GhNAC66	NAP	OG5_177391	rcom|29950.m001173	1	-133	69	99
GhNAC67	NAC1	OG5_170224	osat|NP_001053312	4	-83	57	97
GhNAC68	SENU5	OG5_212763	rcom|30128.m009035	1	-117	81	100
GhNAC69	ANAC011	OG5_139552	rcom|30171.m000407	1	-172	55	91
GhNAC70	ONAC003	OG5_213021	rcom|29738.m001046	1	-170	67	95
GhNAC71	NAP	OG5_135169	rcom|29851.m002359	1	-131	69	99
GhNAC72	OsNAC7	OG5_140455	rcom|30147.m014211	1	-141	70	100
GhNAC73	OsNAC7	OG5_170479	rcom|30068.m002591	1	-141	64	95
GhNAC74	TIP	OG5_190295	rcom|29764.m000758	1	-102	44	88
GhNAC75	No group	OG5_167608	rcom|30076.m004476	4	-30	39	75
GhNAC76	NAP	OG5_135169	rcom|27961.m000091	1	-142	71	100
GhNAC77	TERN	OG5_244818	rcom|30032.m000479	2	-67	58	70
GhNAC78	NAC1	OG5_170224	osat|NP_001053312	6	-83	57	97

GhNAC79	OsNAC7	OG5_170428	rcom|28623.m000400	4	-77	89	98
GhNAC80	No group	OG5_139552	atha|NP_188400	3	-31	50	52
GhNAC81	OsNAC7	OG5_170479	rcom|27964.m000369	1	-140	74	99
GhNAC82	No group	NO_GROUP	rcom|30169.m006458	3	-56	56	66
GhNAC83	OsNAC7	OG5_170428	rcom|28623.m000400	2	-77	89	98
GhNAC84	OsNAC7	OG5_170479	rcom|30068.m002591	1	-158	69	100
GhNAC85	ATAF	NO_GROUP	rcom|30193.m000709	8	-88	53	99
GhNAC86	No group	OG5_178238	rcom|29797.m000353	1	-104	65	100
GhNAC87	OsNAC7	OG5_140455	rcom|28200.m000189	1	-163	77	100
GhNAC88	NAP	OG5_135169	rcom|27961.m000091	1	-140	72	100
GhNAC89	NAM	OG5_150285	rcom|27950.m000105	1	-123	70	100
GhNAC90	No group	OG5_178154	rcom|29683.m000467	7	-49	51	95
GhNAC91	AtNAC3	OG5_135169	rcom|28219.m000090	1	-132	70	100
GhNAC92	NAP	OG5_135169	rcom|27961.m000091	1	-154	77	100
GhNAC93	OsNAC7	OG5_160167	rcom|29725.m000235	8	-75	67	100
GhNAC94	No group	OG5_189953	rcom|30170.m014236	1	-125	65	97
GhNAC95	NAP	OG5_177391	rcom|29950.m001173	1	-112	61	95
GhNAC96	OsNAC7	OG5_170479	rcom|30068.m002591	1	-170	74	100
GhNAC97	ONAC022	OG5_177548	rcom|29813.m001479	1	-135	65	100
GhNAC98	ONAC022	OG5_177548	rcom|29813.m001479	1	-137	65	100
GhNAC99	No group	OG5_213067	rcom|29813.m001519	7	-38	44	68
GhNAC100	No group	NO_GROUP	rcom|30170.m013849	5	-75	57	97
GhNAC101	NAP	OG5_245102	rcom|30078.m002348	1	-63	64	56
GhNAC102	No group	OG5_189953	rcom|30170.m014236	1	-137	64	100
GhNAC103	ATAF	OG5_135169	rcom|29648.m002012	1	-141	79	99
GhNAC104	No group	NO_GROUP	rcom|30131.m007019	1	-71	55	93

GhNAC105	OsNAC7	OG5_170428	rcom|28623.m000400	1	-102	59	100
GhNAC106	OsNAC7	OG5_140455	rcom|28200.m000189	1	-172	82	100
GhNAC107	No group	OG5_213067	rcom|29813.m001519	2	-43	50	90
GhNAC108	No group	NO_GROUP	rcom|30169.m006458	3	-46	50	65
GhNAC109	No group	OG5_213067	rcom|29813.m001519	6	-46	49	62
GhNAC110	ANAC011	OG5_190411	rcom|29827.m002555	1	-115	65	97
GhNAC111	No group	OG5_178154	rcom|29683.m000467	1	-56	72	95
GhNAC112	ONAC022	OG5_190119	rcom|30169.m006343	2	-89	54	100
GhNAC113	OsNAC8	OG5_212233	rcom|30055.m001612	1	-123	63	93
GhNAC114	TERN	OG5_156371	atha|NP_001118771	1	-69	54	98
GhNAC115	ATAF	OG5_135169	rcom|29648.m002012	1	-128	75	98
GhNAC116	ANAC011	OG5_190411	rcom|29827.m002555	1	-130	72	96
GhNAC117	No group	NO_GROUP	rcom|30131.m007019	4	-53	48	95
GhNAC118	OsNAC7	OG5_170428	rcom|28623.m000400	3	-77	88	97
GhNAC119	No group	OG5_178238	rcom|29797.m000353	1	-97	63	97
GhNAC120	TERN	OG5_156371	atha|NP_001118771	1	-70	56	99
GhNAC121	OsNAC8	OG5_212233	rcom|30055.m001612	1	-123	62	93
GhNAC122	No group	NO_GROUP	rcom|29830.m001415	1	-66	69	92
GhNAC123	No group	OG5_167608	rcom|30076.m004477	3	-29	37	86
GhNAC124	No group	OG5_167608	rcom|30076.m004477	1	-37	44	84
GhNAC125	TIP	OG5_190295	rcom|29764.m000758	1	-102	44	87
GhNAC126	ONAC022	OG5_190119	rcom|30169.m006343	6	-78	51	94
GhNAC127	OsNAC8	OG5_212233	rcom|30055.m001612	1	-119	60	91
GhNAC128	No group	OG5_164698	rcom|30063.m001417	1	-136	70	91
GhNAC129	No group	OG5_177391	atha|NP_001118568	9	-22	34	87
GhNAC130	No group	NO_GROUP	rcom|30131.m007019	2	-69	56	93

GhNAC131	NAP	OG5_245102	rcom|30078.m002348	1	-102	70	87
GhNAC132	NAM	OG5_190207	rcom|29974.m000235	1	-115	71	100
GhNAC133	No group	OG5_178154	rcom|29683.m000467	2	-48	48	97
GhNAC134	ONAC022	OG5_190119	rcom|30169.m006343	1	-89	57	95
GhNAC135	ONAC022	OG5_177548	rcom|29813.m001479	1	-125	62	100
GhNAC136	OsNAC7	OG5_140455	rcom|28200.m000189	1	-164	80	100
GhNAC137	TERN	OG5_244818	rcom|30032.m000479	3	-60	50	96
GhNAC138	OsNAC7	OG5_170479	rcom|27964.m000369	1	-143	73	99
GhNAC139	ATAF	OG5_135169	rcom|29648.m002012	1	-141	78	99
GhNAC140	TERN	OG5_156371	atha|NP_001118771	8	-77	56	99
GhNAC141	NAM	OG5_164656	rcom|30138.m004055	1	-160	74	100
GhNAC142	ONAC022	OG5_190119	rcom|30169.m006343	2	-80	55	97
GhNAC143	NAM	OG5_150285	rcom|28226.m000851	1	-128	68	100
GhNAC144	No group	OG5_189953	rcom|30170.m014236	1	-136	64	100
GhNAC145	NAP	OG5_213067	rcom|29813.m001519	1	-113	73	96
GhNAC146	NAC1	OG5_170224	osat|NP_001053312	1	-80	57	96
GhNAC147	TIP	OG5_190295	rcom|29764.m000758	1	-101	44	87
GhNAC148	NAC2	OG5_160086	atha|NP_850554	1	-123	53	100
GhNAC149	NAM	OG5_164511	rcom|29950.m001171	1	-109	56	100
GhNAC150	ANAC011	OG5_139552	rcom|30171.m000407	1	-178	52	100
GhNAC151	No group	OG5_135169	ppat|e_gw1.140.53.1	2	-28	39	86
GhNAC152	SENU5	OG5_212763	rcom|30128.m009035	1	-114	79	100
GhNAC153	SENU5	OG5_212763	rcom|30128.m009035	1	-112	79	100
GhNAC154	NAM	OG5_190207	rcom|29974.m000235	1	-114	70	100
GhNAC155	No group	OG5_178238	rcom|29797.m000353	1	-105	67	96
GhNAC156	ONAC003	OG5_190453	rcom|29912.m005354	1	-179	73	96

GhNAC157	No group	OG5_178238	rcom|29797.m000353	1	-103	66	95
GhNAC158	No group	NO_GROUP	rcom|30131.m007019	1	-54	49	95
GhNAC159	ONAC003	OG5_170647	rcom|29983.m003125	1	-144	85	100
GhNAC160	NAP	OG5_135169	rcom|27961.m000091	1	-144	72	100
GhNAC161	NAC2	OG5_160086	atha|NP_566374	1	-121	54	99
GhNAC162	NAM	OG5_164511	rcom|29950.m001171	1	-111	57	100
GhNAC163	No group	NO_GROUP	rcom|29830.m001415	6	-68	69	93
GhNAC164	NAM	OG5_164511	rcom|29950.m001171	1	-123	62	100
GhNAC165	NAP	OG5_177391	rcom|29950.m001173	1	-131	69	100
GhNAC166	No group	OG5_189953	rcom|30170.m014236	1	-124	65	93
GhNAC167	ATAF	NO_GROUP	rcom|30193.m000709	1	-109	68	100
GhNAC168	NAM	OG5_150285	rcom|28226.m000851	1	-132	73	100
GhNAC169	No group	NO_GROUP	rcom|30147.m014441	1	-54	69	90
GhNAC170	No group	NO_GROUP	rcom|30131.m007019	2	-69	56	93
GhNAC171	OsNAC7	OG5_170428	rcom|28623.m000400	1	-76	87	98
GhNAC172	ONAC022	OG5_190119	rcom|30169.m006343	7	-81	56	95
GhNAC173	No group	OG5_139552	rcom|28872.m000248	1	-146	48	99
GhNAC174	NAC2	OG5_160086	atha|NP_974272	1	-118	53	99
GhNAC175	ONAC003	OG5_213021	rcom|29738.m001046	1	-169	67	99
GhNAC176	ONAC022	OG5_177548	rcom|29032.m000021	1	-143	65	100
GhNAC177	No group	OG5_178238	rcom|29797.m000353	1	-97	63	97
GhNAC178	No group	OG5_177627	rcom|29168.m000389	2	-82	49	61
GhNAC179	OsNAC7	OG5_170479	rcom|27964.m000369	1	-144	74	99
GhNAC180	TERN	OG5_156371	rcom|30055.m001588	1	-78	62	98
GhNAC181	NAC1	OG5_170224	osat|NP_001053312	2	-80	58	96
GhNAC182	ONAC022	OG5_177548	rcom|29032.m000021	1	-143	66	100

GhNAC183	No group	OG5_164698	rcom|30063.m001417	1	-139	67	100
GhNAC184	OsNAC7	OG5_170479	rcom|30068.m002591	1	-153	68	100
GhNAC185	ONAC003	OG5_170647	rcom|30190.m010767	1	-130	71	100
GhNAC186	OsNAC7	OG5_170479	rcom|27964.m000369	1	-138	73	99
GhNAC187	OsNAC7	OG5_170479	rcom|30068.m002591	1	-159	70	100
GhNAC188	No group	OG5_139552	rcom|28872.m000248	1	-133	46	99
GhNAC189	ATAF	OG5_135169	rcom|29648.m002012	1	-126	73	98
GhNAC190	ONAC022	OG5_177548	rcom|29813.m001479	1	-125	61	100
GhNAC191	ONAC003	OG5_170647	rcom|30190.m010767	1	-124	72	99
GhNAC192	No group	OG5_160072	atha|NP_567986	2	-38	29	82
GhNAC193	OsNAC7	OG5_140455	rcom|30147.m014211	1	-120	66	98
GhNAC194	OsNAC7	OG5_170428	rcom|28623.m000400	1	-129	70	100
GhNAC195	NAP	OG5_135169	rcom|29851.m002359	1	-124	66	100
GhNAC196	TERN	OG5_244818	rcom|30032.m000479	1	-79	60	96
GhNAC197	ONAC022	OG5_190119	osat|NP_001051438	1	-47	59	55
GhNAC198	ONAC003	OG5_170647	rcom|30190.m010767	1	-124	72	99
GhNAC199	NAP	OG5_135169	rcom|29851.m002359	1	-125	67	100
GhNAC200	No group	OG5_164698	rcom|30063.m001417	1	-138	72	96
GhNAC201	TIP	OG5_160072	rcom|30068.m002619	1	-131	44	98
GhNAC202	ONAC022	OG5_177548	rcom|29813.m001479	1	-136	65	100
GhNAC203	NAP	OG5_213067	rcom|29813.m001519	1	-118	72	100
GhNAC204	NAM	OG5_164656	rcom|30138.m004055	1	-158	73	100
GhNAC205	OsNAC7	OG5_212126	rcom|29728.m000815	2	-96	56	99
GhNAC206	ONAC022	OG5_177548	rcom|29813.m001479	1	-125	61	100
GhNAC207	No group	OG5_160025	atha|NP_564440	1	-120	44	98
GhNAC208	OsNAC7	OG5_170479	rcom|27964.m000369	1	-147	77	99

GhNAC209	No group	OG5_164698	rcom|30063.m001417	1	-136	70	92
GhNAC210	OsNAC7	OG5_170479	rcom|30068.m002591	1	-150	67	100
GhNAC211	OsNAC7	OG5_170479	rcom|30068.m002591	1	-170	73	100

TABLE S4 The structural analysis of GrNAC, GaNAC and GhNAC identified in this study.

Name	Extron number	Intron number	Protein length (aa)	Molecular weight	Theoretical pI	α-helix	Extended strand	β-turn	Random coil
GrNAC1	3	2	299	33896.23	6.10	48	51	19	181
GrNAC2	6	5	435	49270.34	4.82	113	82	38	202
GrNAC3	3	2	300	33773.98	8.10	55	62	28	155
GrNAC4	3	2	334	38433.15	8.67	74	63	31	166
GrNAC5	3	2	386	43363.87	7.55	85	57	35	209
GrNAC6	3	2	255	29069.11	9.41	56	49	22	128
GrNAC7	2	1	271	30619.28	4.96	88	46	20	117
GrNAC8	3	2	359	39577.22	7.72	75	81	31	172
GrNAC9	3	2	405	46062.24	5.62	99	77	39	190
GrNAC10	3	2	346	38429.11	9.00	66	81	24	175
GrNAC11	3	2	335	37069.62	8.32	57	70	20	188
GrNAC12	6	5	450	51543.90	5.90	134	99	53	164
GrNAC13	3	2	358	40651.09	7.69	55	97	24	182
GrNAC14	3	2	201	22945.42	8.62	52	37	15	97
GrNAC15	2	1	201	23406.44	5.07	47	49	19	86
GrNAC16	3	2	358	40537.64	8.79	81	63	22	192
GrNAC17	3	2	257	29845.33	5.31	62	64	24	107
GrNAC18	2	1	183	21455.65	8.85	25	57	20	81
GrNAC19	3	2	389	43877.79	7.02	81	84	27	197
GrNAC20	6	5	607	69181.85	5.25	167	121	34	285
GrNAC21	4	3	443	49577.76	6.30	144	53	37	209
GrNAC22	3	2	419	47739.32	6.85	97	70	22	230
GrNAC23	4	3	281	32536.67	8.28	57	62	20	142
GrNAC24	2	1	335	39102.88	6.43	114	55	25	141

GrNAC25	3	2	279	32440.95	7.69	97	50	10	122
GrNAC26	4	3	449	49852.03	5.65	155	57	38	199
GrNAC27	3	2	357	41577.26	6.15	122	62	19	154
GrNAC28	3	2	366	42408.28	6.52	123	57	19	167
GrNAC29	4	3	327	37368.53	5.54	49	83	18	177
GrNAC30	3	2	321	37013.74	6.76	63	51	27	180
GrNAC31	3	2	327	38075.39	5.38	118	52	20	137
GrNAC32	2	1	155	17709.55	9.51	22	39	18	76
GrNAC33	3	2	226	26260.68	7.01	75	38	17	96
GrNAC34	7	6	397	44986.01	5.25	84	90	33	190
GrNAC35	2	1	177	20557.48	9.41	32	50	15	80
GrNAC36	6	5	635	70808.10	5.01	173	118	54	290
GrNAC37	5	4	541	60544.78	4.91	151	118	36	236
GrNAC38	3	2	357	39531.29	7.71	78	74	32	173
GrNAC39	5	4	397	45147.69	4.68	96	88	36	177
GrNAC40	3	2	254	29340.96	6.11	79	51	25	99
GrNAC41	3	2	254	28879.76	9.23	58	56	13	127
GrNAC42	4	3	322	37027.44	4.94	103	42	19	158
GrNAC43	3	2	344	39060.69	7.33	86	46	26	186
GrNAC44	3	2	285	32326.17	5.57	65	58	29	133
GrNAC45	3	2	288	32859.70	5.80	70	54	23	141
GrNAC46	3	2	289	33220.07	5.81	73	63	24	129
GrNAC47	3	2	406	46322.59	5.86	67	97	40	202
GrNAC48	3	2	228	25888.05	6.10	75	32	15	106
GrNAC49	4	3	371	41637.21	8.54	98	83	37	153
GrNAC50	3	2	383	43560.52	6.14	93	85	34	171

GrNAC51	9	8	859	94097.41	4.86	211	149	60	439
GrNAC52	3	2	388	44086.39	6.67	81	84	40	183
GrNAC53	5	4	525	58544.30	5.78	142	106	45	232
GrNAC54	1	0	256	28955.24	5.61	81	60	25	90
GrNAC55	3	2	576	63139.19	4.91	117	140	72	247
GrNAC56	3	2	279	32349.10	8.89	98	47	12	122
GrNAC57	3	2	281	32687.37	8.59	85	49	8	139
GrNAC58	4	3	317	36080.31	5.21	92	39	15	171
GrNAC59	3	2	344	38484.08	6.76	67	71	17	189
GrNAC60	3	2	342	38353.29	8.85	85	74	31	152
GrNAC61	3	2	302	35068.26	5.76	64	69	21	148
GrNAC62	3	2	204	23728.96	5.36	64	42	26	72
GrNAC63	4	3	643	49018.97	8.02	207	107	54	275
GrNAC64	5	4	643	72404.10	5.05	207	107	54	275
GrNAC65	4	3	467	53677.29	5.68	95	96	46	230
GrNAC66	3	2	321	36507.11	8.49	67	77	27	150
GrNAC67	3	2	368	42599.72	5.47	79	44	37	208
GrNAC68	11	10	573	65618.03	6.53	162	86	49	276
GrNAC69	3	2	373	42466.94	5.08	83	75	43	172
GrNAC70	3	2	355	40618.80	5.21	74	77	40	164
GrNAC71	3	2	233	27292.81	8.31	55	52	23	103
GrNAC72	3	2	294	34068.11	6.11	56	69	26	143
GrNAC73	5	4	562	62549.69	5.42	177	117	49	219
GrNAC74	3	2	256	29592.98	4.91	55	51	27	123
GrNAC75	6	5	424	47788.04	4.70	99	94	22	209
GrNAC76	6	5	359	40305.81	4.74	54	98	28	179

GrNAC77	4	3	219	25228.65	8.22	37	54	26	102
GrNAC78	6	5	375	42996.98	4.92	90	83	32	170
GrNAC79	8	7	482	55123.98	4.35	129	93	32	228
GrNAC80	4	3	651	73602.67	4.84	228	105	35	283
GrNAC81	3	2	327	36980.51	5.47	92	55	26	154
GrNAC82	3	2	312	36471.81	6.19	94	71	18	129
GrNAC83	3	2	350	40710.44	6.36	117	66	30	137
GrNAC84	4	3	326	37200.40	5.67	36	77	22	191
GrNAC85	3	2	260	30580.24	4.94	69	49	21	121
GrNAC86	6	5	591	66330.23	4.75	157	116	40	278
GrNAC87	4	3	450	50281.27	5.76	150	53	33	214
GrNAC88	3	2	340	39095.80	6.03	74	65	18	183
GrNAC89	3	2	279	32171.24	5.77	88	64	29	98
GrNAC90	3	2	215	24594.50	6.23	45	44	9	117
GrNAC91	3	2	349	39816.46	5.96	95	57	29	168
GrNAC92	7	6	558	63434.81	5.53	177	113	48	220
GrNAC93	3	2	349	38900.07	5.73	121	50	32	146
GrNAC94	3	2	195	22444.95	4.96	46	26	16	107
GrNAC95	3	2	202	23198.73	5.53	49	40	21	92
GrNAC96	1	0	239	26905.38	5.99	43	59	27	110
GrNAC97	3	2	268	30969.66	5.81	71	60	26	111
GrNAC98	5	4	440	50432.71	4.58	124	89	49	178
GrNAC99	3	2	393	44314.22	6.05	83	78	33	199
GrNAC100	3	2	263	29703.82	5.44	81	50	18	114
GrNAC101	2	1	138	16089.78	9.64	26	39	17	56
GrNAC102	4	3	410	45820.59	8.76	127	68	29	186

GrNAC103	3	2	310	36572.03	6.43	104	55	17	134
GrNAC104	3	2	404	45898.28	6.63	115	71	35	183
GrNAC105	3	2	318	36123.06	8.05	69	80	24	145
GrNAC106	8	7	1190	132488.52	8.31	326	210	91	563
GrNAC107	1	0	347	38362.15	4.74	84	55	39	169
GrNAC108	3	2	310	35816.99	5.03	119	51	23	117
GrNAC109	2	1	157	18128.01	9.60	47	24	13	73
GrNAC110	3	2	331	37447.50	7.62	65	79	27	160
GrNAC111	3	2	276	31551.30	5.92	50	52	23	151
GrNAC112	3	2	380	42415.17	8.35	73	61	27	219
GrNAC113	3	2	321	36026.69	9.20	64	79	36	142
GrNAC114	3	2	340	37985.76	9.36	71	64	26	179
GrNAC115	3	2	395	43922.54	5.97	118	77	23	177
GrNAC116	5	4	360	39624.93	4.76	93	72	41	154
GrNAC117	3	2	323	37177.67	5.11	104	68	23	128
GrNAC118	6	5	658	74559.97	5.54	216	118	55	269
GrNAC119	3	2	288	32683.88	9.12	56	83	41	119
GrNAC120	3	2	245	27879.00	9.57	49	53	19	124
GrNAC121	3	2	257	29221.60	6.26	77	42	21	117
GrNAC122	3	2	377	42972.71	6.28	98	67	42	170
GrNAC123	6	5	378	43067.96	5.25	79	98	28	173
GrNAC124	7	6	452	51291.10	5.05	126	85	36	205
GrNAC125	3	2	343	39752.64	5.16	99	64	24	156
GrNAC126	5	4	447	50223.45	6.81	102	102	37	206
GrNAC127	4	3	338	38469.82	5.62	42	61	16	219
GrNAC128	4	3	298	34180.50	8.16	53	69	19	157

GrNAC129	3	2	386	43032.85	6.76	101	52	30	203
GrNAC130	2	1	178	20529.66	8.67	45	36	10	87
GrNAC131	6	5	474	53600.39	6.34	104	90	34	246
GrNAC132	3	2	298	33899.42	6.54	79	53	20	146
GrNAC133	3	2	321	35923.62	9.00	57	67	25	172
GrNAC134	3	2	349	38829.65	8.42	38	83	30	198
GrNAC135	3	2	349	38640.15	6.32	59	80	34	176
GrNAC136	3	2	349	39481.39	6.71	51	93	43	162
GrNAC137	3	2	359	40355.39	8.12	109	50	24	176
GrNAC138	3	2	324	36222.92	8.22	70	50	21	183
GrNAC139	3	2	367	42396.47	5.26	121	76	29	141
GrNAC140	3	2	291	33579.92	8.48	93	54	27	117
GrNAC141	3	2	306	34192.33	8.88	38	88	31	149
GrNAC142	3	2	256	29186.96	9.48	48	67	15	126
GaNAC1	3	2	567	62298.96	4.93	131	119	79	238
GaNAC2	3	2	279	32328.17	9.12	99	42	12	126
GaNAC3	3	2	281	32706.48	8.64	89	47	10	135
GaNAC4	3	2	197	22509.93	5.38	42	197	23	88
GaNAC5	2	1	216	24442.19	4.97	66	47	24	79
GaNAC6	3	2	226	26288.71	6.76	82	33	22	89
GaNAC7	3	2	283	32336.36	5.22	97	53	25	108
GaNAC8	3	2	340	39456.27	5.16	102	70	22	146
GaNAC9	3	2	234	26988.69	9.16	86	36	16	96
GaNAC10	2	1	151	17753.18	8.67	34	37	13	67
GaNAC11	5	4	331	37730.43	5.21	109	37	17	168
GaNAC12	3	2	185	21152.18	8.54	37	51	20	77

GaNAC13	5	4	533	59328.98	5.71	136	105	50	242
GaNAC14	3	2	256	29647.15	4.98	62	48	28	118
GaNAC15	3	2	260	30113.91	6.23	92	55	24	89
GaNAC16	2	1	335	39019.74	6.43	120	52	25	138
GaNAC17	3	2	263	29635.67	5.73	79	50	20	114
GaNAC18	6	5	433	49261.38	4.51	105	100	39	189
GaNAC19	1	0	362	42135.90	5.24	59	123	27	153
GaNAC20	3	2	288	32743.97	9.12	48	80	37	123
GaNAC21	5	4	626	70866.06	4.89	235	94	35	262
GaNAC22	3	2	395	43930.58	6.09	122	73	23	177
GaNAC23	2	1	201	23377.36	4.96	40	55	21	85
GaNAC24	3	2	226	26119.38	5.63	66	32	22	106
GaNAC25	3	2	194	22581.87	7.06	58	42	11	83
GaNAC26	2	1	170	20569.83	9.08	49	49	18	54
GaNAC27	2	1	162	18924.28	9.46	41	26	21	74
GaNAC28	4	3	323	37188.69	5.00	90	50	21	162
GaNAC29	3	2	340	38739.28	7.05	76	49	27	188
GaNAC30	4	3	442	49478.61	6.30	141	51	37	213
GaNAC31	3	2	281	32531.68	7.71	61	60	20	140
GaNAC32	4	3	343	37823.35	7.74	74	76	29	164
GaNAC33	3	2	348	38636.31	8.94	67	76	28	177
GaNAC34	3	2	335	37095.66	8.31	58	72	23	182
GaNAC35	5	4	322	35926.05	4.49	110	72	22	118
GaNAC36	3	2	350	40724.38	6.12	121	63	31	135
GaNAC37	3	2	248	28197.10	9.07	43	56	28	121
GaNAC38	6	5	375	42948.22	6.04	62	89	23	201

GaNAC39	5	4	559	62297.76	4.85	144	132	39	244
GaNAC40	3	2	406	46476.83	6.02	73	87	29	217
GaNAC41	3	2	281	31760.73	7.09	78	58	26	119
GaNAC42	4	3	631	70908.19	5.22	218	100	46	267
GaNAC43	3	2	276	31592.34	5.90	46	57	22	151
GaNAC44	6	5	484	54499.02	6.56	112	106	38	228
GaNAC45	1	0	348	38726.52	4.70	90	53	31	174
GaNAC46	3	2	246	28370.70	5.83	69	42	12	123
GaNAC47	3	2	255	29012.69	9.27	46	62	18	129
GaNAC48	3	2	254	28902.78	9.21	47	57	22	128
GaNAC49	3	2	232	27092.54	7.70	56	48	22	106
GaNAC50	3	2	321	36105.79	9.19	58	86	35	142
GaNAC51	2	1	153	18126.54	9.08	37	41	15	60
GaNAC52	7	6	608	69045.60	5.32	168	117	36	287
GaNAC53	3	2	349	38929.09	5.95	127	52	28	142
GaNAC54	3	2	238	27337.56	4.91	55	41	13	129
GaNAC55	5	4	382	43840.24	7.03	74	77	21	210
GaNAC56	3	2	388	44113.23	6.50	85	75	47	181
GaNAC57	4	3	407	45653.96	8.42	75	103	30	199
GaNAC58	7	6	423	47190.44	6.28	76	111	45	191
GaNAC59	3	2	347	38932.54	7.79	71	71	14	191
GaNAC60	3	2	342	38355.12	8.89	89	71	27	155
GaNAC61	5	4	352	38418.59	4.79	93	71	34	154
GaNAC62	4	3	399	44732.40	8.76	113	72	30	184
GaNAC63	7	6	487	55404.96	5.76	150	82	37	218
GaNAC64	5	4	398	44981.53	4.69	96	83	36	183

GaNAC65	3	2	318	36112.98	8.05	69	78	23	148
GaNAC66	3	2	377	43016.76	6.28	98	66	38	175
GaNAC67	6	5	378	43351.40	5.33	76	101	30	171
GaNAC68	7	6	451	51229.84	5.14	130	87	45	189
GaNAC69	3	2	198	22848.45	4.83	52	31	11	104
GaNAC70	3	2	319	36279.96	8.84	68	75	23	153
GaNAC71	3	2	299	33878.22	6.18	49	49	21	180
GaNAC72	3	2	349	38651.13	6.16	61	76	33	179
GaNAC73	3	2	349	38902.70	8.67	41	80	29	199
GaNAC74	3	2	363	42233.11	6.52	121	60	21	161
GaNAC75	3	2	357	39411.01	7.12	80	71	24	182
GaNAC76	9	8	859	94091.62	4.94	215	153	71	420
GaNAC77	6	5	485	54807.95	6.40	112	96	36	241
GaNAC78	3	2	348	39480.45	7.07	52	91	39	161
GaNAC79	3	2	356	40110.18	8.46	106	58	26	166
GaNAC80	3	2	357	41556.29	6.15	126	59	17	155
GaNAC81	2	1	145	17123.39	8.78	34	38	16	57
GaNAC82	3	2	321	36981.83	7.04	69	46	26	180
GaNAC83	6	5	447	50242.56	6.24	132	98	39	178
GaNAC84	3	2	268	31045.73	5.69	68	62	28	110
GaNAC85	3	2	398	45338.64	6.63	105	71	36	186
GaNAC86	3	2	245	27945.97	9.55	45	57	18	125
GaNAC87	4	3	325	37178.39	5.26	66	71	18	170
GaNAC88	3	2	310	36564.21	6.56	110	48	19	133
GaNAC89	3	2	358	40547.52	8.59	88	64	22	184
GaNAC90	3	2	285	32437.43	5.77	74	56	33	122

GaNAC91	3	2	288	32961.81	5.88	69	55	24	140
GaNAC92	3	2	288	33102.96	5.95	63	62	25	138
GaNAC93	3	2	254	28950.77	9.28	55	59	10	130
GaNAC94	3	2	203	23562.94	9.03	52	48	17	86
GaNAC95	3	2	366	42053.43	5.76	84	48	40	194
GaNAC96	3	2	370	42504.82	5.76	124	45	33	168
GaNAC97	3	2	249	28409.01	9.11	53	49	21	126
GaNAC98	3	2	276	31902.29	6.03	62	53	19	142
GaNAC99	4	3	359	40311.65	8.81	82	86	39	152
GaNAC100	3	2	228	25821.95	6.10	70	33	14	111
GaNAC101	3	2	383	43661.58	6.19	94	84	34	171
GaNAC102	3	2	195	22458.98	4.96	44	27	15	109
GaNAC103	2	1	268	30136.85	4.81	82	62	25	99
GaNAC104	2	1	268	30383.35	4.96	81	56	32	99
GaNAC105	2	1	263	29617.52	5.26	77	59	28	99
GaNAC106	3	2	279	32210.22	5.66	87	62	32	98
GaNAC107	3	2	286	33069.32	8.21	39	72	19	156
GaNAC108	3	2	203	23013.21	9.41	58	45	19	84
GaNAC109	3	2	260	30574.36	5.00	73	43	27	117
GaNAC110	4	3	461	51332.36	5.50	142	58	34	227
GaNAC111	8	7	632	70749.75	4.77	169	126	48	289
GaNAC112	3	2	293	33859.77	5.67	63	73	27	130
GaNAC113	3	2	300	33746.93	7.04	61	64	31	144
GaNAC114	3	2	312	35020.69	6.22	73	69	27	143
GaNAC115	3	2	386	43410.93	7.55	69	59	42	216
GaNAC116	3	2	393	44237.09	6.05	85	79	33	196

GaNAC117	3	2	291	33594.15	7.64	100	49	12	130
GaNAC118	4	3	452	50079.23	5.65	161	59	35	197
GaNAC119	4	3	305	34893.48	7.01	70	63	19	153
GaNAC120	3	2	310	35849.23	8.73	67	57	21	165
GaNAC121	3	2	298	33939.52	6.26	87	49	25	137
GaNAC122	5	4	562	62592.63	5.60	153	130	47	232
GaNAC123	5	4	429	48652.54	4.86	118	84	37	188
GaNAC124	2	1	266	30096.95	4.90	89	46	32	99
GaNAC125	3	2	327	37021.56	5.39	91	61	34	141
GaNAC126	3	2	315	36135.64	6.06	62	66	26	161
GaNAC127	6	5	635	70748.30	5.10	166	112	49	308
GaNAC128	3	2	332	37458.50	8.33	66	74	29	163
GaNAC129	6	5	429	48742.28	5.35	106	93	29	201
GaNAC130	3	2	349	39714.40	6.27	108	50	30	161
GaNAC131	3	2	320	35764.47	9.00	52	71	27	170
GaNAC132	3	2	282	32615.35	5.42	63	61	18	140
GaNAC133	3	2	254	28877.40	6.67	76	44	16	118
GaNAC134	3	2	377	41989.74	7.64	90	54	32	201
GaNAC135	3	2	341	38011.71	9.29	66	73	27	175
GaNAC136	3	2	291	33554.89	8.68	92	55	28	118
GaNAC137	3	2	306	34249.36	8.64	30	88	25	163
GaNAC138	7	6	441	50516.50	5.14	116	93	48	184
GaNAC139	3	2	354	39717.70	9.41	56	87	21	190
GaNAC140	3	2	371	41425.21	8.50	79	63	30	199
GaNAC141	3	2	406	46118.31	5.62	101	76	37	192
GaNAC142	3	2	374	42785.03	8.87	95	56	24	199

GhNAC1	3	2	263	29617.63	5.73	78	51	18	116
GhNAC2	3	2	310	36560.08	6.57	113	53	18	126
GhNAC3	7	6	660	73742.58	5.01	199	108	51	302
GhNAC4	3	2	255	28944.84	9.22	56	51	21	127
GhNAC5	3	2	283	32392.43	5.33	108	49	25	101
GhNAC6	5	4	494	55452.69	5.87	154	82	48	210
GhNAC7	3	2	281	32612.30	8.40	82	49	9	141
GhNAC8	4	3	266	30507.26	5.09	66	52	22	126
GhNAC9	2	1	317	36318.32	9.13	76	92	37	112
GhNAC10	2	1	152	17938.35	8.36	34	39	13	66
GhNAC11	2	1	157	18100.92	9.51	46	27	13	71
GhNAC12	3	2	332	37715.81	8.09	73	72	25	162
GhNAC13	3	2	276	31537.28	5.92	52	52	24	148
GhNAC14	3	2	388	43860.80	7.02	86	81	28	193
GhNAC15	4	3	323	37208.68	5.00	88	54	18	163
GhNAC16	3	2	338	38357.69	6.33	76	52	28	182
GhNAC17	3	2	285	32369.27	5.77	68	59	30	128
GhNAC18	3	2	288	32975.84	5.88	71	53	24	140
GhNAC19	3	2	288	33156.97	5.95	62	63	24	139
GhNAC20	3	2	356	40399.56	9.01	83	64	26	183
GhNAC21	5	4	402	45472.16	4.69	103	86	38	175
GhNAC22	1	0	347	38363.19	4.78	79	58	37	173
GhNAC23	3	2	318	36216.15	8.34	70	82	21	145
GhNAC24	2	1	192	22422.75	6.54	71	45	10	66
GhNAC25	6	5	613	68674.83	5.08	215	101	44	253
GhNAC26	3	2	349	38895.78	8.61	37	85	31	196

GhNAC27	3	2	349	38670.28	6.52	59	83	33	174
GhNAC28	7	6	582	65701.86	4.85	213	95	35	239
GhNAC29	3	2	375	42878.87	4.78	140	62	22	151
GhNAC30	6	5	527	58895.17	7.98	169	93	51	214
GhNAC31	3	2	239	27555.59	9.13	72	47	14	106
GhNAC32	3	2	576	63347.31	4.86	121	138	69	248
GhNAC33	3	2	256	29582.95	4.84	57	51	27	121
GhNAC34	6	5	656	74293.71	5.48	212	114	49	281
GhNAC35	1	0	348	38754.57	4.70	87	55	32	174
GhNAC36	5	4	344	38985.08	8.67	87	74	33	150
GhNAC37	3	2	318	36112.00	8.34	69	77	25	147
GhNAC38	3	2	268	31018.71	5.69	68	62	27	111
GhNAC39	3	2	232	27179.71	8.32	56	47	20	109
GhNAC40	3	2	202	23126.67	5.66	49	41	22	90
GhNAC41	4	3	410	45848.67	8.61	121	70	31	188
GhNAC42	5	4	288	32683.88	9.12	45	83	41	119
GhNAC43	3	2	149	17139.50	9.30	31	28	11	79
GhNAC44	3	2	281	32478.57	7.71	59	60	22	140
GhNAC45	7	6	440	50051.87	5.68	100	101	37	202
GhNAC46	3	2	343	39798.71	5.24	97	64	27	155
GhNAC47	3	2	289	33078.85	5.81	68	66	22	133
GhNAC48	3	2	286	32646.39	5.63	66	55	30	135
GhNAC49	3	2	285	32318.19	5.48	64	57	28	136
GhNAC50	3	2	345	39199.81	7.35	83	48	27	187
GhNAC51	4	3	323	37198.64	5.00	96	50	17	160
GhNAC52	10	9	827	90918.40	4.92	210	148	65	404

GhNAC53	3	2	388	44094.17	6.64	85	79	48	176
GhNAC54	3	2	248	28206.07	9.11	42	54	26	126
GhNAC55	3	2	419	47766.35	6.85	98	69	24	228
GhNAC56	3	2	281	32531.68	7.71	61	60	20	140
GhNAC57	4	3	338	38524.87	6.03	57	67	14	200
GhNAC58	4	3	363	39995.69	7.12	80	73	28	182
GhNAC59	4	3	231	26627.15	9.27	52	49	19	111
GhNAC60	4	3	276	31468.31	7.53	32	65	16	163
GhNAC61	2	1	150	17634.01	8.65	23	37	9	81
GhNAC62	3	2	406	46484.85	6.13	69	86	31	220
GhNAC63	3	2	340	39555.41	5.24	104	71	19	146
GhNAC64	3	2	313	36029.74	6.31	55	72	31	155
GhNAC65	3	2	349	39529.43	6.71	51	93	41	164
GhNAC66	3	2	359	40341.36	8.12	109	50	24	176
GhNAC67	3	2	293	33878.83	5.92	63	73	29	128
GhNAC68	3	2	253	28808.63	9.16	59	58	11	125
GhNAC69	7	6	609	69125.59	5.22	170	113	43	283
GhNAC70	6	5	454	51512.04	4.85	118	91	41	204
GhNAC71	3	2	343	38165.88	9.29	66	71	22	184
GhNAC72	2	1	335	39009.79	6.74	121	49	26	139
GhNAC73	3	2	406	46465.76	5.97	67	93	36	210
GhNAC74	5	4	541	60604.84	4.92	153	118	36	234
GhNAC75	6	5	460	52622.03	5.76	132	105	51	172
GhNAC76	3	2	345	38559.10	6.32	68	72	19	186
GhNAC77	4	3	277	32611.33	9.48	87	54	24	112
GhNAC78	3	2	293	33827.73	5.56	71	69	29	124

GhNAC79	2	1	156	18253.47	5.67	43	39	18	56
GhNAC80	7	6	465	53250.58	5.59	149	77	48	191
GhNAC81	3	2	349	39675.32	5.96	98	55	25	171
GhNAC82	3	2	260	30595.38	5.00	80	41	25	114
GhNAC83	2	1	153	18138.60	9.08	36	43	14	60
GhNAC84	3	2	393	44266.17	6.05	83	79	35	196
GhNAC85	4	3	336	38802.24	8.08	68	68	22	178
GhNAC86	3	2	279	32466.99	7.69	97	47	9	126
GhNAC87	3	2	366	42435.31	6.52	122	60	19	165
GhNAC88	3	2	349	38858.66	8.61	42	78	30	199
GhNAC89	3	2	349	38629.11	6.16	61	75	32	181
GhNAC90	3	2	200	23215.36	5.61	61	41	29	69
GhNAC91	3	2	346	38436.06	8.87	65	80	25	176
GhNAC92	3	2	335	37084.59	8.32	55	70	20	190
GhNAC93	3	2	204	23746.98	9.64	66	38	21	79
GhNAC94	3	2	349	38908.11	5.73	117	54	32	146
GhNAC95	3	2	358	40584.63	8.74	94	63	20	181
GhNAC96	3	2	401	45531.69	5.82	97	79	36	189
GhNAC97	3	2	387	43220.09	6.76	109	49	30	199
GhNAC98	3	2	387	43134.00	7.11	101	53	32	201
GhNAC99	3	2	575	63141.93	4.89	132	122	72	249
GhNAC100	3	2	281	31594.61	6.51	81	54	29	120
GhNAC101	3	2	315	36137.70	5.93	63	66	23	163
GhNAC102	3	2	395	43943.55	5.91	117	76	25	177
GhNAC103	3	2	299	33878.22	6.18	49	49	21	180
GhNAC104	3	2	248	28093.04	9.28	45	54	27	122

GhNAC105	3	2	283	32320.32	5.22	97	52	23	111
GhNAC106	3	2	350	40706.37	6.17	125	60	29	136
GhNAC107	3	2	185	21489.93	9.75	42	45	26	72
GhNAC108	3	2	280	32499.63	7.77	87	39	18	136
GhNAC109	3	2	356	40793.95	5.13	72	74	35	175
GhNAC110	4	3	327	37350.52	5.74	52	83	20	172
GhNAC111	3	2	154	17561.00	8.96	32	32	10	80
GhNAC112	3	2	320	36382.00	8.50	68	74	27	151
GhNAC113	4	3	371	41536.04	8.50	88	89	42	152
GhNAC114	3	2	228	25839.97	6.10	72	35	13	108
GhNAC115	3	2	298	33857.34	6.54	85	49	23	141
GhNAC116	4	3	338	38386.65	5.61	53	62	16	207
GhNAC117	3	2	204	23204.43	8.90	58	44	11	91
GhNAC118	2	1	151	17739.15	8.67	33	38	11	69
GhNAC119	3	2	279	32357.12	8.91	103	43	12	121
GhNAC120	3	2	228	25855.99	6.10	74	34	17	103
GhNAC121	4	3	371	41625.14	8.54	98	82	37	154
GhNAC122	3	2	226	26245.71	7.01	70	40	16	100
GhNAC123	6	5	378	43097.00	5.20	80	96	28	174
GhNAC124	7	6	451	51302.94	5.01	124	83	41	203
GhNAC125	5	4	562	62656.77	5.68	153	131	48	230
GhNAC126	3	2	332	38376.98	8.58	71	62	32	167
GhNAC127	4	3	410	45801.54	8.64	131	68	31	180
GhNAC128	3	2	404	45831.19	6.63	119	66	35	184
GhNAC129	3	2	196	22172.85	5.40	56	35	15	90
GhNAC130	3	2	245	27824.90	9.55	46	59	21	119

GhNAC131	3	2	342	39266.08	6.41	78	64	22	178
GhNAC132	3	2	323	36121.82	8.22	70	50	20	183
GhNAC133	3	2	202	23449.61	5.44	57	46	29	70
GhNAC134	3	2	321	36477.02	8.49	67	76	27	151
GhNAC135	3	2	380	42405.23	8.32	73	62	28	217
GhNAC136	3	2	357	41596.40	6.43	130	61	22	144
GhNAC137	4	3	226	25770.68	5.51	74	30	14	108
GhNAC138	3	2	383	43508.47	6.14	95	87	34	167
GhNAC139	3	2	301	34019.30	6.10	50	51	20	180
GhNAC140	3	2	246	28360.79	5.65	56	41	9	140
GhNAC141	4	3	414	46689.29	8.45	67	111	28	208
GhNAC142	3	2	291	33549.89	8.48	89	56	29	117
GhNAC143	4	3	341	38126.22	9.02	60	80	30	171
GhNAC144	3	2	395	43986.65	5.97	132	67	24	172
GhNAC145	4	3	292	33609.75	6.91	50	67	20	155
GhNAC146	3	2	279	32304.39	5.56	98	57	31	93
GhNAC147	5	4	562	62565.69	5.49	165	124	49	224
GhNAC148	4	3	449	49884.07	5.59	151	59	38	201
GhNAC149	3	2	327	36969.51	5.55	92	55	26	154
GhNAC150	6	5	655	74236.68	5.74	192	127	54	282
GhNAC151	6	5	481	54764.49	4.76	159	82	51	189
GhNAC152	3	2	255	29012.69	9.27	46	62	18	129
GhNAC153	3	2	254	28902.78	9.21	47	57	22	128
GhNAC154	3	2	322	35969.64	8.22	74	49	19	180
GhNAC155	3	2	291	33666.22	6.97	102	49	10	130
GhNAC156	7	6	479	53908.22	6.50	102	101	42	234

GhNAC157	3	2	281	32746.55	8.64	93	42	13	133
GhNAC158	3	2	204	23142.33	9.27	60	43	16	85
GhNAC159	3	2	288	32806.95	9.12	45	82	38	123
GhNAC160	3	2	346	38817.39	7.13	70	72	16	188
GhNAC161	4	3	462	51417.51	5.44	144	61	36	221
GhNAC162	3	2	327	36971.50	5.39	91	56	26	154
GhNAC163	3	2	226	26318.74	6.76	82	33	20	91
GhNAC164	3	2	348	39480.45	7.07	52	91	39	166
GhNAC165	3	2	354	39895.96	8.17	106	56	23	169
GhNAC166	3	2	366	40963.50	6.17	137	55	28	146
GhNAC167	3	2	276	31932.31	5.83	61	54	19	142
GhNAC168	3	2	320	35734.39	9.00	46	71	29	174
GhNAC169	3	2	157	18097.88	9.37	47	27	12	71
GhNAC170	3	2	245	27824.90	9.55	46	59	21	119
GhNAC171	2	1	153	18127.49	8.56	37	42	14	60
GhNAC172	3	2	291	33554.89	8.68	92	55	28	116
GhNAC173	8	7	601	67208.04	4.59	152	123	42	284
GhNAC174	4	3	461	51470.56	5.78	148	59	36	218
GhNAC175	6	5	432	48939.88	4.86	120	82	41	189
GhNAC176	3	2	386	43356.78	6.71	71	60	41	214
GhNAC177	3	2	279	32357.12	8.91	103	43	12	121
GhNAC178	10	9	941	106306.48	5.24	321	175	95	359
GhNAC179	3	2	383	43627.57	6.19	94	86	36	167
GhNAC180	3	2	246	28369.76	6.25	67	42	12	125
GhNAC181	3	2	279	32210.22	5.66	87	62	32	98
GhNAC182	3	2	387	43488.03	7.55	78	63	36	210

GhNAC183	3	2	419	47723.28	6.85	96	67	24	232
GhNAC184	3	2	388	44140.39	6.70	79	82	43	184
GhNAC185	3	2	321	36109.78	9.19	65	81	35	140
GhNAC186	3	2	349	39728.45	6.22	105	50	27	167
GhNAC187	3	2	393	44235.08	6.05	86	79	30	198
GhNAC188	6	5	581			146	125	42	268
GhNAC189	3	2	298	33972.55	6.16	85	55	26	132
GhNAC190	3	2	380	42517.35	8.03	84	63	29	204
GhNAC191	3	2	306	34192.33	8.88	38	88	31	149
GhNAC192	8	7	632	71711.84	6.44	177	125	54	276
GhNAC193	3	2	310	36392.97	6.43	105	50	17	138
GhNAC194	3	2	321	36888.28	5.10	99	72	22	128
GhNAC195	3	2	341	38060.68	9.33	48	88	20	185
GhNAC196	3	2	254	28951.54	7.05	80	43	14	117
GhNAC197	6	5	275	31668.24	8.83	65	61	23	126
GhNAC198	3	2	306	34340.52	8.92	30	88	28	160
GhNAC199	3	2	341	37923.55	9.16	48	87	21	185
GhNAC200	3	2	390	44586.90	8.22	95	57	24	214
GhNAC201	6	5	635	70786.26	5.10	158	121	51	305
GhNAC202	3	2	387	43182.00	7.11	97	54	30	206
GhNAC203	3	2	286	33131.40	8.70	47	74	20	145
GhNAC204	3	2	358	40569.97	7.69	58	91	24	185
GhNAC205	3	2	283	32731.53	5.52	62	63	19	139
GhNAC206	3	2	380	42517.35	8.03	84	63	29	204
GhNAC207	5	4	643	72460.08	5.27	224	108	49	262
GhNAC208	3	2	377	43002.73	6.28	98	67	41	171

GhNAC209	3	2	403	45813.21	6.36	111	68	37	187
GhNAC210	3	2	388	44196.35	6.86	89	77	47	175
GhNAC211	3	2	406	46107.28	5.62	102	80	38	186

TABLE S5 Subcellular localization of NAC proteins in *G.raimondii, G.arboreum* and *G.hirsutum* through PSORT and Cello software.

Name	PSORT result	Cello result
GrNAC1	Nuclear protein	Cytoplasmic protein
GrNAC2	Nuclear protein	Nuclear protein
GrNAC3	Nuclear protein	Nuclear protein
GrNAC4	Nuclear protein	Nuclear protein
GrNAC5	Nuclear protein	Nuclear protein
GrNAC6	Nuclear protein	Nuclear protein
GrNAC7	Nuclear protein	Nuclear protein
GrNAC8	Nuclear protein	Nuclear protein
GrNAC9	Nuclear protein	Nuclear protein
GrNAC10	Nuclear protein	Nuclear protein
GrNAC11	Nuclear protein	Nuclear protein
GrNAC12	Nuclear protein	Nuclear protein
GrNAC13	Nuclear protein	Nuclear protein
GrNAC14	Nuclear protein	Nuclear protein
GrNAC15	Cytoplasmic protein	Nuclear protein
GrNAC16	Nuclear protein	Nuclear protein
GrNAC17	Nuclear protein	Nuclear protein
GrNAC18	Mitochondrial protein	PlasmaMembrane protein
GrNAC19	Nuclear protein	Nuclear protein
GrNAC20	PlasmaMembrane protein	Cytoplasmic protein
GrNAC21	Chloroplast protein	Nuclear protein
GrNAC22	Nuclear protein	Nuclear protein
GrNAC23	Nuclear protein	Nuclear protein
GrNAC24	Peroxisomal protein	Nuclear protein
GrNAC25	Cytoplasmic protein	Nuclear protein
GrNAC26	Chloroplast protein	Nuclear protein
GrNAC27	Nuclear protein	Nuclear protein
GrNAC28	Peroxisomal protein	Nuclear protein
GrNAC29	Nuclear protein	Nuclear protein
GrNAC30	Nuclear protein	Nuclear protein
GrNAC31	Chloroplast protein	PlasmaMembrane protein
GrNAC32	Chloroplast protein	Cytoplasmic protein
GrNAC33	Cytoplasmic protein	Nuclear protein
GrNAC34	Nuclear protein	Nuclear protein
GrNAC35	Nuclear protein	Nuclear protein
GrNAC36	Nuclear protein	Nuclear protein
GrNAC37	Cytoplasmic protein	Nuclear protein
GrNAC38	Nuclear protein	Chloroplast protein
GrNAC39	Nuclear protein	Nuclear protein
GrNAC40	Chloroplast protein	Nuclear protein
GrNAC41	Nuclear protein	Nuclear protein

GrNAC42	Nuclear protein	Nuclear protein
GrNAC43	Nuclear protein	Nuclear protein
GrNAC44	Nuclear protein	Nuclear protein
GrNAC45	Nuclear protein	Nuclear protein
GrNAC46	Nuclear protein	Nuclear protein
GrNAC47	Nuclear protein	Nuclear protein
GrNAC48	Nuclear protein	Extracellular protein
GrNAC49	Cytoplasmic protein	Nuclear protein
GrNAC50	Nuclear protein	Nuclear protein
GrNAC51	Nuclear protein	Nuclear protein
GrNAC52	Nuclear protein	Nuclear protein
GrNAC53	Chloroplast protein	Cytoplasmic protein
GrNAC54	Chloroplast protein	Nuclear protein
GrNAC55	Nuclear protein	Extracellular protein
GrNAC56	Nuclear protein	Nuclear protein
GrNAC57	Nuclear protein	Nuclear protein
GrNAC58	Nuclear protein	Nuclear protein
GrNAC59	Nuclear protein	Nuclear protein
GrNAC60	Nuclear protein	Nuclear protein
GrNAC61	Nuclear protein	Nuclear protein
GrNAC62	Nuclear protein	Cytoplasmic protein
GrNAC63	Nuclear protein	Nuclear protein
GrNAC64	Nuclear protein	Nuclear protein
GrNAC65	Nuclear protein	Nuclear protein
GrNAC66	Nuclear protein	Nuclear protein
GrNAC67	Nuclear protein	Nuclear protein
GrNAC68	Nuclear protein	Nuclear protein
GrNAC69	Nuclear protein	Nuclear protein
GrNAC70	Nuclear protein	Cytoplasmic protein
GrNAC71	Nuclear protein	Nuclear protein
GrNAC72	Peroxisomal protein	Nuclear protein
GrNAC73	Chloroplast protein	Nuclear protein
GrNAC74	Nuclear protein	Nuclear protein
GrNAC75	Nuclear protein	Nuclear protein
GrNAC76	Nuclear protein	Nuclear protein
GrNAC77	Cytoplasmic protein	Nuclear protein
GrNAC78	Nuclear protein	Nuclear protein
GrNAC79	Nuclear protein	Nuclear protein
GrNAC80	Nuclear protein	Nuclear protein
GrNAC81	Nuclear protein	Nuclear protein
GrNAC82	Nuclear protein	Nuclear protein
GrNAC83	Nuclear protein	Nuclear protein
GrNAC84	Nuclear protein	Nuclear protein
GrNAC85	Nuclear protein	Nuclear protein

GrNAC86	Vacuolar protein	Nuclear protein
GrNAC87	Nuclear protein	Nuclear protein
GrNAC88	Nuclear protein	Nuclear protein
GrNAC89	Chloroplast protein	Nuclear protein
GrNAC90	Nuclear protein	Nuclear protein
GrNAC91	Nuclear protein	Nuclear protein
GrNAC92	Nuclear protein	Nuclear protein
GrNAC93	Nuclear protein	Nuclear protein
GrNAC94	Nuclear protein	Nuclear protein
GrNAC95	Nuclear protein	Nuclear protein
GrNAC96	Nuclear protein	Cytoplasmic protein
GrNAC97	Peroxisomal protein	Nuclear protein
GrNAC98	Nuclear protein	Nuclear protein
GrNAC99	Nuclear protein	Nuclear protein
GrNAC100	Cytoplasmic protein	Cytoplasmic protein
GrNAC101	Cytoplasmic protein	Cytoplasmic protein
GrNAC102	Golgi protein	Nuclear protein
GrNAC103	Nuclear protein	Nuclear protein
GrNAC104	Nuclear protein	Nuclear protein
GrNAC105	Nuclear protein	Nuclear protein
GrNAC106	Nuclear protein	Nuclear protein
GrNAC107	Cytoplasmic protein	Extracellular protein
GrNAC108	Nuclear protein	Nuclear protein
GrNAC109	Nuclear protein	Mitochondrial protein
GrNAC110	Nuclear protein	Cytoplasmic protein
GrNAC111	Nuclear protein	Nuclear protein
GrNAC112	Nuclear protein	Nuclear protein
GrNAC113	Nuclear protein	Nuclear protein
GrNAC114	Nuclear protein	Nuclear protein
GrNAC115	Nuclear protein	Nuclear protein
GrNAC116	Nuclear protein	Nuclear protein
GrNAC117	Nuclear protein	Nuclear protein
GrNAC118	Nuclear protein	Nuclear protein
GrNAC119	Nuclear protein	Nuclear protein
GrNAC120	Nuclear protein	Nuclear protein
GrNAC121	Nuclear protein	Nuclear protein
GrNAC122	Nuclear protein	Nuclear protein
GrNAC123	Nuclear protein	Nuclear protein
GrNAC124	Cytoplasmic protein	Nuclear protein
GrNAC125	Nuclear protein	Nuclear protein
GrNAC126	Nuclear protein	Nuclear protein
GrNAC127	Nuclear protein	Nuclear protein
GrNAC128	Nuclear protein	Nuclear protein
GrNAC129	Nuclear protein	Nuclear protein

GrNAC130	Cytoplasmic protein	Extracellular protein
GrNAC131	Nuclear protein	Nuclear protein
GrNAC132	Nuclear protein	Cytoplasmic protein
GrNAC133	Mitochondrial protein	Nuclear protein
GrNAC134	Nuclear protein	Nuclear protein
GrNAC135	Nuclear protein	Nuclear protein
GrNAC136	Nuclear protein	Nuclear protein
GrNAC137	Nuclear protein	Nuclear protein
GrNAC138	Nuclear protein	Nuclear protein
GrNAC139	Nuclear protein	Nuclear protein
GrNAC140	Nuclear protein	Nuclear protein
GrNAC141	Nuclear protein	Extracellular protein
GrNAC142	Nuclear protein	Nuclear protein
GaNAC1	Nuclear protein	Nuclear protein
GaNAC2	Cytoplasmic protein	Nuclear protein
GaNAC3	Nuclear protein	Nuclear protein
GaNAC4	Nuclear protein	Nuclear protein
GaNAC5	Nuclear protein	Nuclear protein
GaNAC6	Cytoplasmic protein	Cytoplasmic protein
GaNAC7	Cytoplasmic protein	Nuclear protein
GaNAC8	Nuclear protein	Nuclear protein
GaNAC9	Nuclear protein	Nuclear protein
GaNAC10	Cytoplasmic protein	Cytoplasmic protein
GaNAC11	Nuclear protein	Nuclear protein
GaNAC12	Nuclear protein	Cytoplasmic protein
GaNAC13	Chloroplast protein	Cytoplasmic protein
GaNAC14	Nuclear protein	Nuclear protein
GaNAC15	Chloroplast protein	Nuclear protein
GaNAC16	Nuclear protein	Nuclear protein
GaNAC17	Nuclear protein	Cytoplasmic protein
GaNAC18	Cytoplasmic protein	Nuclear protein
GaNAC19	Chloroplast protein	Nuclear protein
GaNAC20	Nuclear protein	Nuclear protein
GaNAC21	Nuclear protein	Nuclear protein
GaNAC22	Nuclear protein	Nuclear protein
GaNAC23	Extracellular protein	Cytoplasmic protein
GaNAC24	Cytoplasmic protein	Nuclear protein
GaNAC25	Cytoplasmic protein	Cytoplasmic protein
GaNAC26	Cytoplasmic protein	Cytoplasmic protein
GaNAC27	Cytoplasmic protein	Nuclear protein
GaNAC28	Nuclear protein	Nuclear protein
GaNAC29	Nuclear protein	Nuclear protein
GaNAC30	Chloroplast protein	Nuclear protein
GaNAC31	Nuclear protein	Nuclear protein

GaNAC32	Nuclear protein	Nuclear protein
GaNAC33	Nuclear protein	Nuclear protein
GaNAC34	Nuclear protein	Nuclear protein
GaNAC35	Chloroplast protein	Nuclear protein
GaNAC36	Nuclear protein	Nuclear protein
GaNAC37	Nuclear protein	Nuclear protein
GaNAC38	Nuclear protein	Nuclear protein
GaNAC39	Nuclear protein	Nuclear protein
GaNAC40	Nuclear protein	Nuclear protein
GaNAC41	Chloroplast protein	Nuclear protein
GaNAC42	Nuclear protein	Nuclear protein
GaNAC43	Nuclear protein	Nuclear protein
GaNAC44	Nuclear protein	Nuclear protein
GaNAC45	Cytoplasmic protein	Cytoplasmic protein
GaNAC46	Nuclear protein	Nuclear protein
GaNAC47	Nuclear protein	Nuclear protein
GaNAC48	Nuclear protein	Nuclear protein
GaNAC49	Nuclear protein	Nuclear protein
GaNAC50	Nuclear protein	Periplasmic protein
GaNAC51	Cytoplasmic protein	Cytoplasmic protein
GaNAC52	Nuclear protein	Nuclear protein
GaNAC53	Nuclear protein	Extracellular protein
GaNAC54	Nuclear protein	Nuclear protein
GaNAC55	Chloroplast protein	Nuclear protein
GaNAC56	Nuclear protein	Extracellular protein
GaNAC57	Nuclear protein	Cytoplasmic protein
GaNAC58	Nuclear protein	Nuclear protein
GaNAC59	Nuclear protein	Extracellular protein
GaNAC60	Nuclear protein	Nuclear protein
GaNAC61	Nuclear protein	Nuclear protein
GaNAC62	Chloroplast protein	OuterMembrane protein
GaNAC63	Nuclear protein	Nuclear protein
GaNAC64	Nuclear protein	Nuclear protein
GaNAC65	Nuclear protein	Periplasmic protein
GaNAC66	Nuclear protein	Nuclear protein
GaNAC67	Nuclear protein	Nuclear protein
GaNAC68	Cytoplasmic protein	Extracellular protein
GaNAC69	Nuclear protein	Nuclear protein
GaNAC70	Nuclear protein	Nuclear protein
GaNAC71	Nuclear protein	Periplasmic protein
GaNAC72	Nuclear protein	Nuclear protein
GaNAC73	Nuclear protein	Nuclear protein
GaNAC74	Peroxisomal protein	Extracellular protein
GaNAC75	Nuclear protein	Chloroplast protein

GaNAC76	Nuclear protein	Nuclear protein
GaNAC77	Nuclear protein	Extracellular protein
GaNAC78	Nuclear protein	Nuclear protein
GaNAC79	Nuclear protein	Nuclear protein
GaNAC80	Nuclear protein	Extracellular protein
GaNAC81	Mitochondrial protein	Cytoplasmic protein
GaNAC82	Nuclear protein	Nuclear protein
GaNAC83	Nuclear protein	Periplasmic protein
GaNAC84	Peroxisomal protein	Nuclear protein
GaNAC85	Nuclear protein	Nuclear protein
GaNAC86	Nuclear protein	Periplasmic protein
GaNAC87	Nuclear protein	Nuclear protein
GaNAC88	Nuclear protein	Nuclear protein
GaNAC89	Nuclear protein	OuterMembrane protein
GaNAC90	Nuclear protein	Nuclear protein
GaNAC91	Nuclear protein	Nuclear protein
GaNAC92	Nuclear protein	Extracellular protein
GaNAC93	Nuclear protein	Nuclear protein
GaNAC94	Nuclear protein	Nuclear protein
GaNAC95	Nuclear protein	Periplasmic protein
GaNAC96	Nuclear protein	Nuclear protein
GaNAC97	Nuclear protein	Nuclear protein
GaNAC98	Nuclear protein	Periplasmic protein
GaNAC99	Chloroplast protein	Nuclear protein
GaNAC100	Nuclear protein	Extracellular protein
GaNAC101	Nuclear protein	Extracellular protein
GaNAC102	Nuclear protein	Nuclear protein
GaNAC103	Nuclear protein	Nuclear protein
GaNAC104	Cytoplasmic protein	Periplasmic protein
GaNAC105	Extracellular protein	Cytoplasmic protein
GaNAC106	Chloroplast protein	Nuclear protein
GaNAC107	Nuclear protein	Periplasmic protein
GaNAC108	Nuclear protein	Nuclear protein
GaNAC109	Nuclear protein	Nuclear protein
GaNAC110	Nuclear protein	Periplasmic protein
GaNAC111	Vacuolar protein	Cytoplasmic protein
GaNAC112	Peroxisomal protein	Nuclear protein
GaNAC113	Nuclear protein	Extracellular protein
GaNAC114	Nuclear protein	Nuclear protein
GaNAC115	Nuclear protein	Nuclear protein
GaNAC116	Nuclear protein	Extracellular protein
GaNAC117	Chloroplast protein	Nuclear protein
GaNAC118	Nuclear protein	Nuclear protein
GaNAC119	Nuclear protein	Extracellular protein

GaNAC120	Nuclear protein	Nuclear protein
GaNAC121	Nuclear protein	Cytoplasmic protein
GaNAC122	Nuclear protein	OuterMembrane protein
GaNAC123	Nuclear protein	Nuclear protein
GaNAC124	Nuclear protein	Nuclear protein
GaNAC125	Nuclear protein	Periplasmic protein
GaNAC126	Nuclear protein	Nuclear protein
GaNAC127	Nuclear protein	Nuclear protein
GaNAC128	Nuclear protein	Periplasmic protein
GaNAC129	Nuclear protein	Nuclear protein
GaNAC130	Nuclear protein	Nuclear protein
GaNAC131	Cytoplasmic protein	Extracellular protein
GaNAC132	Nuclear protein	Nuclear protein
GaNAC133	Nuclear protein	Nuclear protein
GaNAC134	Nuclear protein	Periplasmic protein
GaNAC135	Nuclear protein	Nuclear protein
GaNAC136	Nuclear protein	Nuclear protein
GaNAC137	Nuclear protein	Extracellular protein
GaNAC138	Nuclear protein	Nuclear protein
GaNAC139	Nuclear protein	Nuclear protein
GaNAC140	Nuclear protein	Extracellular protein
GaNAC141	Nuclear protein	Nuclear protein
GaNAC142	Nuclear protein	Nuclear protein
GhNAC1	Mitochondrial protein	Cytoplasmic protein
GhNAC2	Nuclear protein	Nuclear protein
GhNAC3	Nuclear protein	Nuclear protein
GhNAC4	Nuclear protein	Nuclear protein
GhNAC5	Cytoplasmic protein	Nuclear protein
GhNAC6	Vacuolar protein	Nuclear protein
GhNAC7	Nuclear protein	Nuclear protein
GhNAC8	Nuclear protein	Nuclear protein
GhNAC9	Chloroplast protein	PlasmaMembrane protein
GhNAC10	Cytoplasmic protein	Nuclear protein
GhNAC11	Nuclear protein	Mitochondrial protein
GhNAC12	Nuclear protein	Nuclear protein
GhNAC13	Nuclear protein	Nuclear protein
GhNAC14	Nuclear protein	Nuclear protein
GhNAC15	Nuclear protein	Nuclear protein
GhNAC16	Nuclear protein	Nuclear protein
GhNAC17	Nuclear protein	Nuclear protein
GhNAC18	Nuclear protein	Nuclear protein
GhNAC19	Nuclear protein	Nuclear protein
GhNAC20	Nuclear protein	Nuclear protein
GhNAC21	Cytoplasmic protein	Nuclear protein

GhNAC22	Cytoplasmic protein	Extracellular protein
GhNAC23	Nuclear protein	Nuclear protein
GhNAC24	Cytoplasmic protein	Cytoplasmic protein
GhNAC25	Nuclear protein	Nuclear protein
GhNAC26	Nuclear protein	Nuclear protein
GhNAC27	Nuclear protein	Nuclear protein
GhNAC28	Nuclear protein	Nuclear protein
GhNAC29	Cytoplasmic protein	Cytoplasmic protein
GhNAC30	Vacuolar protein	Nuclear protein
GhNAC31	Nuclear protein	Nuclear protein
GhNAC32	Nuclear protein	Nuclear protein
GhNAC33	Nuclear protein	Nuclear protein
GhNAC34	Integral membrane protein	Nuclear protein
GhNAC35	Cytoplasmic protein	Cytoplasmic protein
GhNAC36	Nuclear protein	Nuclear protein
GhNAC37	Nuclear protein	Nuclear protein
GhNAC38	Peroxisomal protein	Nuclear protein
GhNAC39	Nuclear protein	Nuclear protein
GhNAC40	Nuclear protein	Nuclear protein
GhNAC41	Nuclear protein	Nuclear protein
GhNAC42	Nuclear protein	Nuclear protein
GhNAC43	Cytoplasmic protein	Mitochondrial protein
GhNAC44	Nuclear protein	Nuclear protein
GhNAC45	Nuclear protein	Nuclear protein
GhNAC46	Nuclear protein	Nuclear protein
GhNAC47	Nuclear protein	Nuclear protein
GhNAC48	Nuclear protein	Nuclear protein
GhNAC49	Nuclear protein	Nuclear protein
GhNAC50	Nuclear protein	Nuclear protein
GhNAC51	Nuclear protein	Nuclear protein
GhNAC52	Nuclear protein	Nuclear protein
GhNAC53	Nuclear protein	Nuclear protein
GhNAC54	Nuclear protein	Nuclear protein
GhNAC55	Nuclear protein	Nuclear protein
GhNAC56	Nuclear protein	Nuclear protein
GhNAC57	Nuclear protein	Nuclear protein
GhNAC58	Nuclear protein	Nuclear protein
GhNAC59	Nuclear protein	Nuclear protein
GhNAC60	Nuclear protein	Nuclear protein
GhNAC61	Nuclear protein	Cytoplasmic protein
GhNAC62	Nuclear protein	Nuclear protein
GhNAC63	Nuclear protein	Nuclear protein
GhNAC64	Nuclear protein	Nuclear protein
GhNAC65	Nuclear protein	Nuclear protein

GhNAC66	Nuclear protein	Nuclear protein
GhNAC67	Peroxisomal protein	Nuclear protein
GhNAC68	Nuclear protein	Nuclear protein
GhNAC69	Nuclear protein	Nuclear protein
GhNAC70	Nuclear protein	Nuclear protein
GhNAC71	Nuclear protein	Nuclear protein
GhNAC72	Nuclear protein	Nuclear protein
GhNAC73	Nuclear protein	Nuclear protein
GhNAC74	Cytoplasmic protein	Nuclear protein
GhNAC75	Nuclear protein	Nuclear protein
GhNAC76	Nuclear protein	Nuclear protein
GhNAC77	Cytoplasmic protein	Nuclear protein
GhNAC78	Peroxisomal protein	Nuclear protein
GhNAC79	Cytoplasmic protein	Nuclear protein
GhNAC80	Nuclear protein	Nuclear protein
GhNAC81	Nuclear protein	Nuclear protein
GhNAC82	Nuclear protein	Nuclear protein
GhNAC83	Cytoplasmic protein	Cytoplasmic protein
GhNAC84	Nuclear protein	Nuclear protein
GhNAC85	Nuclear protein	Cytoplasmic protein
GhNAC86	Cytoplasmic protein	Nuclear protein
GhNAC87	Peroxisomal protein	Nuclear protein
GhNAC88	Nuclear protein	Nuclear protein
GhNAC89	Nuclear protein	Nuclear protein
GhNAC90	Cytoplasmic protein	Cytoplasmic protein
GhNAC91	Nuclear protein	Nuclear protein
GhNAC92	Nuclear protein	Nuclear protein
GhNAC93	Peroxisomal protein	Nuclear protein
GhNAC94	Nuclear protein	Extracellular protein
GhNAC95	Nuclear protein	Nuclear protein
GhNAC96	Nuclear protein	Nuclear protein
GhNAC97	Nuclear protein	Nuclear protein
GhNAC98	Nuclear protein	Nuclear protein
GhNAC99	Nuclear protein	Extracellular protein
GhNAC100	Chloroplast protein	Nuclear protein
GhNAC101	Nuclear protein	Nuclear protein
GhNAC102	Nuclear protein	Nuclear protein
GhNAC103	Nuclear protein	Cytoplasmic protein
GhNAC104	Nuclear protein	Nuclear protein
GhNAC105	Cytoplasmic protein	Nuclear protein
GhNAC106	Nuclear protein	Nuclear protein
GhNAC107	Nuclear protein	Mitochondrial protein
GhNAC108	Nuclear protein	Nuclear protein
GhNAC109	Nuclear protein	Nuclear protein

GhNAC110	Nuclear protein	Nuclear protein
GhNAC111	Nuclear protein	Nuclear protein
GhNAC112	Nuclear protein	Nuclear protein
GhNAC113	Cytoplasmic protein	Nuclear protein
GhNAC114	Nuclear protein	Extracellular protein
GhNAC115	Nuclear protein	Cytoplasmic protein
GhNAC116	Nuclear protein	Nuclear protein
GhNAC117	Nuclear protein	Nuclear protein
GhNAC118	Cytoplasmic protein	Cytoplasmic protein
GhNAC119	Cytoplasmic protein	Nuclear protein
GhNAC120	Nuclear protein	Extracellular protein
GhNAC121	Cytoplasmic protein	Nuclear protein
GhNAC122	Cytoplasmic protein	Nuclear protein
GhNAC123	Nuclear protein	Nuclear protein
GhNAC124	Nuclear protein	Nuclear protein
GhNAC125	Nuclear protein	Nuclear protein
GhNAC126	Nuclear protein	Nuclear protein
GhNAC127	Nuclear protein	Nuclear protein
GhNAC128	Nuclear protein	Nuclear protein
GhNAC129	Nuclear protein	Nuclear protein
GhNAC130	Nuclear protein	Nuclear protein
GhNAC131	Nuclear protein	Nuclear protein
GhNAC132	Nuclear protein	Nuclear protein
GhNAC133	Nuclear protein	Cytoplasmic protein
GhNAC134	Nuclear protein	Nuclear protein
GhNAC135	Nuclear protein	Nuclear protein
GhNAC136	Nuclear protein	Nuclear protein
GhNAC137	Nuclear protein	Nuclear protein
GhNAC138	Nuclear protein	Nuclear protein
GhNAC139	Nuclear protein	Nuclear protein
GhNAC140	Nuclear protein	Nuclear protein
GhNAC141	Chloroplast protein	Cytoplasmic protein
GhNAC142	Nuclear protein	Nuclear protein
GhNAC143	Mitochondrial protein	Nuclear protein
GhNAC144	Nuclear protein	Nuclear protein
GhNAC145	Nuclear protein	Nuclear protein
GhNAC146	Cytoplasmic protein	Nuclear protein
GhNAC147	Chloroplast protein	Nuclear protein
GhNAC148	Chloroplast protein	Nuclear protein
GhNAC149	Nuclear protein	Nuclear protein
GhNAC150	Integral membrane protein	Nuclear protein
GhNAC151	Nuclear protein	Nuclear protein
GhNAC152	Nuclear protein	Nuclear protein
GhNAC153	Nuclear protein	Nuclear protein

GhNAC154	Nuclear protein	Nuclear protein
GhNAC155	Cytoplasmic protein	Nuclear protein
GhNAC156	Nuclear protein	Nuclear protein
GhNAC157	Nuclear protein	Nuclear protein
GhNAC158	Nuclear protein	Nuclear protein
GhNAC159	Nuclear protein	Nuclear protein
GhNAC160	Nuclear protein	Nuclear protein
GhNAC161	Nuclear protein	Nuclear protein
GhNAC162	Nuclear protein	Nuclear protein
GhNAC163	Cytoplasmic protein	Nuclear protein
GhNAC164	Nuclear protein	Nuclear protein
GhNAC165	Nuclear protein	Nuclear protein
GhNAC166	Chloroplast protein	Extracellular protein
GhNAC167	Nuclear protein	Cytoplasmic protein
GhNAC168	Cytoplasmic protein	Nuclear protein
GhNAC169	Nuclear protein	Mitochondrial protein
GhNAC170	Nuclear protein	Nuclear protein
GhNAC171	Cytoplasmic protein	Cytoplasmic protein
GhNAC172	Nuclear protein	Nuclear protein
GhNAC173	Chloroplast protein	Nuclear protein
GhNAC174	Nuclear protein	Nuclear protein
GhNAC175	Nuclear protein	Nuclear protein
GhNAC176	Nuclear protein	Nuclear protein
GhNAC177	Nuclear protein	Nuclear protein
GhNAC178	Nuclear protein	Nuclear protein
GhNAC179	Nuclear protein	Nuclear protein
GhNAC180	Nuclear protein	Nuclear protein
GhNAC181	Chloroplast protein	Nuclear protein
GhNAC182	Nuclear protein	Nuclear protein
GhNAC183	Nuclear protein	Nuclear protein
GhNAC184	Nuclear protein	Nuclear protein
GhNAC185	Nuclear protein	Nuclear protein
GhNAC186	Nuclear protein	Nuclear protein
GhNAC187	Nuclear protein	Nuclear protein
GhNAC188	Vacuolar protein	Cytoplasmic protein
GhNAC189	Cytoplasmic protein	Cytoplasmic protein
GhNAC190	Nuclear protein	Nuclear protein
GhNAC191	Nuclear protein	Extracellular protein
GhNAC192	Nuclear protein	Nuclear protein
GhNAC193	Nuclear protein	Nuclear protein
GhNAC194	Nuclear protein	Nuclear protein
GhNAC195	Nuclear protein	Nuclear protein
GhNAC196	Nuclear protein	Nuclear protein
GhNAC197	Cytoplasmic protein	Nuclear protein

GhNAC198	Nuclear protein	Extracellular protein
GhNAC199	Nuclear protein	Nuclear protein
GhNAC200	Nuclear protein	Nuclear protein
GhNAC201	Nuclear protein	Nuclear protein
GhNAC202	Nuclear protein	Nuclear protein
GhNAC203	Nuclear protein	Nuclear protein
GhNAC204	Nuclear protein	Nuclear protein
GhNAC205	Nuclear protein	Nuclear protein
GhNAC206	Nuclear protein	Nuclear protein
GhNAC207	Nuclear protein	Nuclear protein
GhNAC208	Nuclear protein	Nuclear protein
GhNAC209	Nuclear protein	Nuclear protein
GhNAC210	Nuclear protein	Nuclear protein
GhNAC211	Nuclear protein	Nuclear protein

TABLE S6 Genomic locations of NAC genes in *G.raimondii*, *G.arboreum and G.hirsutum*.

Name	Chromosome	Start (bp)	End (bp)	Subfamily
GrNAC1	scaffold523	18607	20782	ATAF
GrNAC2	DD08	19446506	19449470	ONAC003
GrNAC3	scaffold427	207831	210957	ONAC022
GrNAC4	scaffold424	140069	145461	ONAC022
GrNAC5	scaffold398	23287	24644	ONAC022
GrNAC6	DD05	35767759	35770302	SENU5
GrNAC7	DD11	42931041	42931997	No group
GrNAC8	scaffold336	402980	404984	NAM
GrNAC9	DD05	5193668	5195087	OsNAC7
GrNAC10	scaffold349	325918	327492	AtNAC3
GrNAC11	scaffold349	381611	383493	NAP
GrNAC12	scaffold385	245120	248463	No group
GrNAC13	DD08	19041902	19043723	NAM
GrNAC14	DD08	30696835	30700915	No group
GrNAC15	DD10	48276683	48277373	No group
GrNAC16	DD13	13658888	13660938	NAP
GrNAC17	scaffold370	358351	362994	No group
GrNAC18	scaffold370	421612	422391	No group
GrNAC19	DD04	1467712	1471997	OsNAC7
GrNAC20	DD04	23789102	23792926	ANAC011
GrNAC21	scaffold285	124061	128984	NAC2
GrNAC22	DD11	13836892	13838992	No group
GrNAC23	DD08	20485845	20487089	NAP
GrNAC24	scaffold141	555239	556329	OsNAC7
GrNAC25	DD10	14336080	14337130	No group
GrNAC26	DD11	39765702	39769804	NAC2
GrNAC27	scaffold136	248497	249907	OsNAC7
GrNAC28	DD03	1768060	1770029	OsNAC7
GrNAC29	DD03	551984	554250	ANAC011
GrNAC30	DD01	6883547	6884703	ONAC022
GrNAC31	DD06	41679482	41681413	ANAC011
GrNAC32	DD11	10477387	10478014	No group
GrNAC33	DD03	25539214	25540841	No group
GrNAC34	DD06	4903122	4905091	ONAC003
GrNAC35	DD12	19206632	19207328	No group
GrNAC36	scaffold214	810487	813956	TIP
GrNAC37	DD06	43211222	43213361	TIP
GrNAC38	DD07	823559	825297	NAM
GrNAC39	DD01	4631377	4632919	No group
GrNAC40	scaffold224	1242876	1247919	NAC1
GrNAC41	DD12	6946131	6947831	SENU5
GrNAC42	DD01	32995624	32998901	ANAC011

GrNAC43	DD01	33008607	33009812	OsNAC7
GrNAC44	DD01	33177700	33179063	ONAC022
GrNAC45	DD01	33188004	33189085	ONAC022
GrNAC46	DD01	33194754	33195918	ONAC022
GrNAC47	DD06	43331208	43332631	OsNAC7
GrNAC48	DD06	32694289	32695151	TERN
GrNAC49	DD06	32761230	32762686	OsNAC8
GrNAC50	DD08	34364388	34365740	OsNAC7
GrNAC51	DD08	7069518	7074605	TIP
GrNAC52	DD08	7167399	7168740	OsNAC7
GrNAC53	DD07	54120905	54123143	TIP
GrNAC54	DD08	21174074	21174844	No group
GrNAC55	DD08	21962301	21965258	No group
GrNAC56	DD08	22413298	22414382	No group
GrNAC57	DD05	42360912	42362623	No group
GrNAC58	DD13	15174710	15177101	ANAC011
GrNAC59	DD09	42650266	42651461	NAP
GrNAC60	DD09	42571526	42573614	AtNAC3
GrNAC61	scaffold132	1569562	1570980	OsNAC7
GrNAC62	DD12	21837258	21838067	No group
GrNAC63	DD12	21626462	21629282	ATAF
GrNAC64	DD05	4323141	4326047	No group
GrNAC65	DD06	22373332	22376861	NAP
GrNAC66	DD02	35017903	35019654	ONAC022
GrNAC67	DD11	44405926	44407205	No group
GrNAC68	DD11	44417086	44421233	No group
GrNAC69	DD11	44655323	44656617	No group
GrNAC70	DD11	44671055	44672296	No group
GrNAC71	DD02	2614449	2615825	TERN
GrNAC72	DD02	3092548	3096211	NAC1
GrNAC73	DD04	8916328	8919435	TIP
GrNAC74	DD01	30607437	30611829	ANAC011
GrNAC75	DD01	614143	615829	No group
GrNAC76	DD01	622491	624491	No group
GrNAC77	DD01	636108	637166	No group
GrNAC78	DD01	637594	639424	No group
GrNAC79	DD01	642353	644449	No group
GrNAC80	DD09	29877369	29881659	No group
GrNAC81	scaffold149	2456784	2458547	NAM
GrNAC82	DD01	23852587	23855805	ONAC022
GrNAC83	DD08	11642561	11643800	OsNAC7
GrNAC84	DD08	12488564	12489946	ANAC011
GrNAC85	DD06	3551727	3554127	No group
GrNAC86	DD09	33294580	33298447	No group

GrNAC87	DD09	33324003	33328221	NAC2
GrNAC88	DD12	10758005	10759260	NAP
GrNAC89	DD01	11350742	11355326	NAC1
GrNAC90	DD01	21588250	21589089	TERN
GrNAC91	DD01	16516147	16517568	OsNAC7
GrNAC92	DD01	16871349	16874497	No group
GrNAC93	DD02	36680837	36682300	No group
GrNAC94	DD05	37547737	37549110	No group
GrNAC95	DD13	26829030	26830440	No group
GrNAC96	DD13	27220018	27220737	No group
GrNAC97	DD08	4809111	4812549	NAC1
GrNAC98	DD08	5377884	5380239	No group
GrNAC99	DD03	10418387	10419718	OsNAC7
GrNAC100	DD11	2317890	2318835	NAP
GrNAC101	scaffold84	3303775	3304287	ONAC022
GrNAC102	DD09	11006055	11009206	OsNAC8
GrNAC103	DD09	13329161	13330760	OsNAC7
GrNAC104	DD10	43487102	43488952	No group
GrNAC105	DD07	49701005	49702174	NAM
GrNAC106	DD07	50076824	50082881	ONAC003
GrNAC107	DD07	50815838	50817800	No group
GrNAC108	DD07	52082867	52084474	OsNAC7
GrNAC109	DD07	52398635	52399279	No group
GrNAC110	DD07	52804748	52805927	NAM
GrNAC111	DD07	53281543	53283054	ANAC011
GrNAC112	DD13	24124865	24126196	ONAC022
GrNAC113	DD09	20304294	20306087	ONAC003
GrNAC114	DD09	20706068	20707256	NAP
GrNAC115	DD09	21860375	21863001	No group
GrNAC116	DD09	23405269	23407026	ANAC063
GrNAC117	DD07	32802328	32804256	OsNAC7
GrNAC118	DD07	34191535	34194611	ANAC011
GrNAC119	DD08	29060608	29062123	ONAC003
GrNAC120	DD10	35972793	35974826	No group
GrNAC121	DD09	37600464	37601810	TERN
GrNAC122	DD07	44187728	44189216	OsNAC7
GrNAC123	DD07	44409047	44410820	No group
GrNAC124	DD07	44412989	44414940	No group
GrNAC125	DD06	28834871	28836078	No group
GrNAC126	DD09	4427421	4430027	ONAC003
GrNAC127	DD08	59803660	59805145	ANAC011
GrNAC128	DD05	17484576	17486704	NAP
GrNAC129	DD05	18338937	18340288	ONAC022
GrNAC130	DD08	65567916	65568556	No group

GrNAC131	DD10	11076771	11081152	ONAC003
GrNAC132	DD05	9663934	9666068	ATAF
GrNAC133	DD10	19965112	19966728	NAM
GrNAC134	DD07	14962361	14964144	NAP
GrNAC135	DD07	14221860	14223937	NAM
GrNAC136	DD07	11707619	11709759	NAM
GrNAC137	DD07	11651526	11654152	NAP
GrNAC138	DD06	16916975	16918370	NAM
GrNAC139	DD08	42468606	42470636	OsNAC7
GrNAC140	DD10	34648233	34649284	ONAC022
GrNAC141	DD10	29163833	29165261	ONAC003
GrNAC142	DD10	25489422	25490392	NAP
GaNAC1	AA07	38796850	38800358	No group
GaNAC2	AA07	39244251	39245326	No group
GaNAC3	AA05	8782518	8783751	No group
GaNAC4	AA13	72659117	72660522	No group
GaNAC5	AA13	72865884	72866692	No group
GaNAC6	AA01	134813812	134815763	No group
GaNAC7	AA01	135231885	135233924	OsNAC7
GaNAC8	AA11	79945697	79946898	No group
GaNAC9	AA06	28949406	28950373	No group
GaNAC10	AA06	29304653	29305334	OsNAC7
GaNAC11	AA13	128581627	128584049	ANAC011
GaNAC12	AA11	21973017	21973797	TIP
GaNAC13	AA11	21969745	21971699	TIP
GaNAC14	AA08	96914418	96919026	ANAC011
GaNAC15	AA09	72325006	72329547	NAC1
GaNAC16	AA01	66529160	66530250	OsNAC7
GaNAC17	AA09	61938175	61939120	NAP
GaNAC18	AA05	25454953	25456821	No group
GaNAC19	AA05	25252224	25253312	No group
GaNAC20	AA06	106538088	106539610	ONAC003
GaNAC21	AA06	105220071	105223072	No group
GaNAC22	AA10	19703687	19706168	No group
GaNAC23	AA07	14050164	14050856	No group
GaNAC24	AA03	89510562	89512144	No group
GaNAC25	AA12	48470419	48473940	ONAC022
GaNAC26	AA03	40961402	40962292	No group
GaNAC27	AA03	40022239	40024990	OsNAC7
GaNAC28	AA03	39915872	39919052	ANAC011
GaNAC29	AA03	39904263	39905455	OsNAC7
GaNAC30	AA12	94506529	94510351	NAC2
GaNAC31	AA06	68304952	68306008	NAP
GaNAC32	AA07	91804306	91806510	NAM

GaNAC33	AA07	90838807	90840038	AtNAC3
GaNAC34	AA07	90755195	90756412	NAP
GaNAC35	AA07	115428614	115430494	ANAC063
GaNAC36	AA06	74392190	74393447	OsNAC7
GaNAC37	AA09	49645246	49646251	No group
GaNAC38	AA06	50563859	50565484	ANAC011
GaNAC39	AA11	63168505	63170698	TIP
GaNAC40	AA11	63284750	63286163	OsNAC7
GaNAC41	AA10	17176312	17178497	No group
GaNAC42	AA05	52700475	52703383	No group
GaNAC43	AA13	44395362	44396816	ANAC011
GaNAC44	AA08	59626428	59630363	ONAC003
GaNAC45	AA04	55219777	55220823	No group
GaNAC46	AA01	106229226	106230158	TERN
GaNAC47	AA08	20627817	20629042	SENU5
GaNAC48	AA08	20671327	20672479	SENU5
GaNAC49	AA02	66461535	66462876	TERN
GaNAC50	AA10	19105833	19107638	ONAC003
GaNAC51	AA06	77076903	77077520	OsNAC7
GaNAC52	AA03	37862931	37866926	ANAC011
GaNAC53	AA07	87959835	87961286	No group
GaNAC54	AA11	61920414	61922290	ANAC011
GaNAC55	AA03	12598977	12600718	ANAC011
GaNAC56	AA06	15727862	15729203	OsNAC7
GaNAC57	AA06	99596159	99601807	NAM
GaNAC58	AA10	97328410	97336345	No group
GaNAC59	AA10	110547688	110548894	NAP
GaNAC60	AA10	110618671	110620323	AtNAC3
GaNAC61	AA08	15378997	15380755	ANAC063
GaNAC62	AA10	70846339	70849476	OsNAC8
GaNAC63	AA12	123497903	123500989	No group
GaNAC64	AA01	128686787	128688331	No group
GaNAC65	AA04	57251943	57253112	NAM
GaNAC66	AA04	137181348	137182861	OsNAC7
GaNAC67	AA04	136879163	136880907	No group
GaNAC68	AA04	136846462	136848414	No group
GaNAC69	AA06	33207788	33208575	No group
GaNAC70	AA13	66974533	66976275	ONAC022
GaNAC71	AA08	45570146	45571677	ATAF
GaNAC72	AA13	111510815	111512855	NAM
GaNAC73	AA13	112537381	112538632	NAP
GaNAC74	AA07	110873497	110875760	OsNAC7
GaNAC75	AA04	134098620	134100349	NAM
GaNAC76	AA09	30748743	30752613	TIP

GaNAC77	AA08	58058041	58061699	ONAC003
GaNAC78	AA04	47812133	47813511	NAM
GaNAC79	AA04	47749278	47750895	NAP
GaNAC80	AA03	91578452	91579864	OsNAC7
GaNAC81	AA04	85324573	85325208	OsNAC7
GaNAC82	AA12	44005068	44006224	ONAC022
GaNAC83	AA04	56378466	56380835	ONAC003
GaNAC84	AA03	1695717	1699103	NAC1
GaNAC85	AA08	63618599	63620427	No group
GaNAC86	AA01	99180845	99181754	No group
GaNAC87	AA07	110625482	110627803	ANAC011
GaNAC88	AA07	71951424	71953017	OsNAC7
GaNAC89	AA13	34067654	34069020	NAP
GaNAC90	AA05	19253822	19255214	ONAC022
GaNAC91	AA05	19263347	19264430	ONAC022
GaNAC92	AA05	19267591	19268771	ONAC022
GaNAC93	AA12	128941829	128942775	SENU5
GaNAC94	AA11	28289455	28293510	No group
GaNAC95	AA13	93689307	93690610	No group
GaNAC96	AA13	93890451	93891753	No group
GaNAC97	AA12	18083582	18084851	No group
GaNAC98	AA12	139034113	139035292	ATAF
GaNAC99	AA11	82061767	82063188	OsNAC8
GaNAC100	AA11	81989128	81989996	TERN
GaNAC101	AA06	89764411	89765758	OsNAC7
GaNAC102	AA05	18915612	18916926	No group
GaNAC103	AA13	67630552	67631498	No group
GaNAC104	AA13	67636007	67636966	No group
GaNAC105	AA13	108849254	108850186	No group
GaNAC106	AA01	31217817	31221934	NAC1
GaNAC107	AA07	51584680	51585748	NAP
GaNAC108	AA12	55623877	55625509	No group
GaNAC109	AA11	101999497	102001894	No group
GaNAC110	AA10	47042086	47045365	NAC2
GaNAC111	AA10	47027657	47031166	No group
GaNAC112	AA01	73425629	73430008	NAC1
GaNAC113	AA10	83927673	83930773	ONAC022
GaNAC114	AA07	106666513	106671272	No group
GaNAC115	AA11	10802655	10804010	ONAC022
GaNAC116	AA04	100671007	100672367	OsNAC7
GaNAC117	AA06	62420244	62421319	No group
GaNAC118	AA09	24180680	24183960	NAC2
GaNAC119	AA12	24058491	24059701	NAP
GaNAC120	AA11	18703973	18709108	ONAC022

GaNAC121	AA11	38870296	38871539	ATAF
GaNAC122	AA03	48763376	48765712	TIP
GaNAC123	AA01	68144390	68147039	ONAC003
GaNAC124	AA09	77547336	77548289	No group
GaNAC125	AA13	96788700	96789987	NAM
GaNAC126	AA11	92473027	92475658	NAP
GaNAC127	AA13	11677129	11679821	TIP
GaNAC128	AA10	63385531	63386720	NAM
GaNAC129	AA11	101574624	101577789	ONAC003
GaNAC130	AA08	101283142	101284571	OsNAC7
GaNAC131	AA08	115936993	115938619	NAM
GaNAC132	AA13	54389321	54390730	OsNAC7
GaNAC133	AA12	59546705	59547847	TERN
GaNAC134	AA06	42857750	42859086	ONAC022
GaNAC135	AA11	22686782	22687972	NAP
GaNAC136	AA08	104363190	104364241	ONAC022
GaNAC137	AA10	105705297	105706727	ONAC003
GaNAC138	AA03	50492674	50496954	No group
GaNAC139	AA08	2319168	2320434	NAP
GaNAC140	AA13	138567858	138569163	ONAC022
GaNAC141	AA06	102438224	102439647	OsNAC7
GaNAC142	AA08	87769062	87770996	No group
GhNAC1	scaffold26.1	802451	803396	NAP
GhNAC2	Dt03	10804784	10806380	OsNAC7
GhNAC3	Dt03	17416915	17420804	TIP
GhNAC4	Dt05	18192141	18193281	SENU5
GhNAC5	Dt05	17072946	17074993	OsNAC7
GhNAC6	Dt09	41090557	41092454	TIP
GhNAC7	Dt05	7121212	7122439	No group
GhNAC8	Dt06	6570560	6572429	ANAC011
GhNAC9	At09	42867893	42869653	No group
GhNAC10	Dt07	6580248	6580933	OsNAC7
GhNAC11	Dt07	6930685	6931316	No group
GhNAC12	Dt07	7360261	7361444	NAM
GhNAC13	Dt07	7859999	7861510	ANAC011
GhNAC14	Dt05	13084713	13089023	OsNAC7
GhNAC15	Dt05	13201920	13205091	ANAC011
GhNAC16	Dt05	13220542	13221728	OsNAC7
GhNAC17	Dt05	13435846	13437181	ONAC022
GhNAC18	Dt05	13445309	13446392	ONAC022
GhNAC19	Dt05	13449625	13450937	ONAC022
GhNAC20	At08	75913882	75915226	NAP
GhNAC21	scaffold56.1	47493	49038	No group
GhNAC22	Dt07	3292885	3293928	No group

GhNAC23	Dt07	4421250	4422419	NAM
GhNAC24	At09	84935515	84937173	ONAC022
GhNAC25	Dt05	51029009	51031911	No group
GhNAC26	Dt13	23877386	23878630	NAP
GhNAC27	Dt13	23128200	23130274	NAM
GhNAC28	Dt11	5062051	5065054	No group
GhNAC29	Dt11	5065345	5067167	No group
GhNAC30	Dt09	36686817	36690650	NAC2
GhNAC31	scaffold280.1	69848	70810	No group
GhNAC32	scaffold280.1	537030	540030	No group
GhNAC33	Dt01	40072969	40077403	ANAC011
GhNAC34	Dt07	18841508	18844585	ANAC011
GhNAC35	At07	6197059	6198105	No group
GhNAC36	At07	7056340	7058221	ONAC003
GhNAC37	At07	7424736	7426278	NAM
GhNAC38	scaffold171.1	1620990	1624348	NAC1
GhNAC39	scaffold42.1	1407742	1409085	TERN
GhNAC40	Dt13	50548515	50549930	No group
GhNAC41	At09	54038986	54042135	OsNAC8
GhNAC42	Dt08	40576670	40578184	ONAC003
GhNAC43	At08	42194413	42195045	No group
GhNAC44	Dt08	44725569	44726626	NAP
GhNAC45	Dt06	39501261	39504184	ONAC003
GhNAC46	Dt06	13022312	13023520	No group
GhNAC47	scaffold246.1	427765	428956	ONAC022
GhNAC48	scaffold246.1	431957	433034	ONAC022
GhNAC49	scaffold246.1	442092	443468	ONAC022
GhNAC50	scaffold246.1	642420	643628	OsNAC7
GhNAC51	scaffold246.1	653635	656925	ANAC011
GhNAC52	scaffold304.1	823705	827578	TIP
GhNAC53	scaffold304.1	957135	958476	OsNAC7
GhNAC54	Dt11	7297426	7298408	No group
GhNAC55	Dt11	7686008	7688098	No group
GhNAC56	Dt13	42607744	42608800	NAP
GhNAC57	Dt13	8212709	8214185	ANAC011
GhNAC58	At02	7741248	7743259	NAM
GhNAC59	At07	11959745	11961047	No group
GhNAC60	Dt08	49402253	49403485	ANAC011
GhNAC61	scaffold120.1	537943	538475	ANAC011
GhNAC62	scaffold377.1	993115	994523	OsNAC7
GhNAC63	At06	49063318	49064519	No group
GhNAC64	Dt06	18218180	18220778	NAP
GhNAC65	At10	26941953	26943315	NAM
GhNAC66	At10	26978352	26979980	NAP

GhNAC67	At09	48615958	48619658	NAC1
GhNAC68	Dt12	8141148	8142090	SENU5
GhNAC69	Dt04	31615008	31618944	ANAC011
GhNAC70	Dt08	45475968	45482922	ONAC003
GhNAC71	Dt11	3597167	3598363	NAP
GhNAC72	Dt07	24908295	24909385	OsNAC7
GhNAC73	Dt06	5809930	5811353	OsNAC7
GhNAC74	Dt06	5710218	5712357	TIP
GhNAC75	Dt04	9540364	9543748	No group
GhNAC76	Dt09	4343006	4344204	NAP
GhNAC77	scaffold238.1	870527	871881	TERN
GhNAC78	At07	30766914	30770834	NAC1
GhNAC79	Dt07	21731777	21732408	OsNAC7
GhNAC80	Dt01	55156828	55159763	No group
GhNAC81	Dt01	55506619	55508037	OsNAC7
GhNAC82	At06	5747770	5750165	No group
GhNAC83	Dt08	32473928	32474547	OsNAC7
GhNAC84	Dt03	40989093	40990418	OsNAC7
GhNAC85	At09	62829978	62831367	ATAF
GhNAC86	Dt08	1221482	1222529	No group
GhNAC87	Dt13	12501153	12503117	OsNAC7
GhNAC88	At13	17537663	17538914	NAP
GhNAC89	At13	16606261	16608301	NAM
GhNAC90	At09	59527628	59532065	No group
GhNAC91	At02	4983711	4984929	AtNAC3
GhNAC92	At02	5058438	5059652	NAP
GhNAC93	Dt02	42118042	42118909	OsNAC7
GhNAC94	Dt02	42614718	42616174	No group
GhNAC95	At13	21672208	21673564	NAP
GhNAC96	Dt04	13512221	13513630	OsNAC7
GhNAC97	Dt05	2675998	2677358	ONAC022
GhNAC98	Dt05	2677885	2679240	ONAC022
GhNAC99	scaffold944.1	522541	526031	No group
GhNAC100	Dt09	53428860	53430932	No group
GhNAC101	At06	42330515	42333149	NAP
GhNAC102	Dt09	54485378	54488004	No group
GhNAC103	At10	67551053	67552584	ATAF
GhNAC104	At11	58136248	58137253	No group
GhNAC105	scaffold1588.1	176523	178555	OsNAC7
GhNAC106	At05	8501297	8502550	OsNAC7
GhNAC107	scaffold1227.1	260257	260987	No group
GhNAC108	scaffold1227.1	271878	272905	No group
GhNAC109	scaffold1227.1	275571	276814	No group
GhNAC110	scaffold854.1	98467	100729	ANAC011

GhNAC111	At08	41668721	41669384	No group
GhNAC112	scaffold732.1	260229	261971	ONAC022
GhNAC113	At06	46040186	46041643	OsNAC8
GhNAC114	At06	45972154	45973022	TERN
GhNAC115	Dt05	48560986	48562226	ATAF
GhNAC116	scaffold842.1	245216	246700	ANAC011
GhNAC117	At04	76333856	76335696	No group
GhNAC118	At07	5567333	5568014	OsNAC7
GhNAC119	scaffold1755.1	248281	249355	No group
GhNAC120	Dt06	14563745	14564607	TERN
GhNAC121	Dt06	14625717	14627173	OsNAC8
GhNAC122	scaffold1977.1	229103	230878	No group
GhNAC123	Dt07	8667290	8669052	No group
GhNAC124	Dt07	8663041	8664993	No group
GhNAC125	Dt02	24916547	24918883	TIP
GhNAC126	Dt13	14194745	14199922	ONAC022
GhNAC127	Dt09	63144883	63148034	OsNAC8
GhNAC128	Dt10	55662322	55664168	No group
GhNAC129	At06	50723534	50724914	No group
GhNAC130	scaffold1383.1	314814	315723	No group
GhNAC131	Dt12	31714359	31715600	NAP
GhNAC132	Dt06	26985063	26986456	NAM
GhNAC133	scaffold1379.1	215432	216235	No group
GhNAC134	scaffold1464.1	6593	8344	ONAC022
GhNAC135	Dt13	45700306	45701654	ONAC022
GhNAC136	scaffold544.1	324928	326340	OsNAC7
GhNAC137	Dt12	38657634	38658769	TERN
GhNAC138	Dt08	34497069	34498413	OsNAC7
GhNAC139	Dt10	25345004	25346509	ATAF
GhNAC140	Dt01	48081157	48082439	TERN
GhNAC141	Dt08	46921207	46922947	NAM
GhNAC142	Dt10	48762619	48763669	ONAC022
GhNAC143	Dt10	54080204	54081826	NAM
GhNAC144	Dt09	54731012	54733488	No group
GhNAC145	At12	95051404	95053605	NAP
GhNAC146	Dt01	61881128	61885270	NAC1
GhNAC147	Dt04	3772544	3774868	TIP
GhNAC148	Dt11	49115097	49118345	NAC2
GhNAC149	At13	3795855	3797142	NAM
GhNAC150	At01	53363219	53366268	ANAC011
GhNAC151	scaffold2321.1	246701	249093	No group
GhNAC152	At05	46781442	46782667	SENU5
GhNAC153	At05	46817750	46818902	SENU5
GhNAC154	At06	21106233	21107622	NAM

GhNAC155	At08	46113260	46114337	No group
GhNAC156	At09	49560643	49564555	ONAC003
GhNAC157	scaffold2526.1	121292	122525	No group
GhNAC158	At12	30935852	30937487	No group
GhNAC159	scaffold1107.1	45291	46812	ONAC003
GhNAC160	At03	99301754	99302955	NAP
GhNAC161	At09	31632364	31635635	NAC2
GhNAC162	At13	54620728	54622015	NAM
GhNAC163	At04	23428217	23430141	No group
GhNAC164	At01	44922711	44924085	NAM
GhNAC165	At01	44972723	44974331	NAP
GhNAC166	At02	16972570	16974069	No group
GhNAC167	scaffold1819.1	268777	269956	ATAF
GhNAC168	At10	16958072	16959698	NAM
GhNAC169	At07	5295190	5295826	No group
GhNAC170	At13	23173143	23174052	No group
GhNAC171	At08	34980527	34981142	OsNAC7
GhNAC172	At10	20264435	20265487	ONAC022
GhNAC173	Dt09	34520876	34523954	No group
GhNAC174	Dt09	34495689	34498959	NAC2
GhNAC175	scaffold1127.1	41675	44726	ONAC003
GhNAC176	At06	8417715	8419070	ONAC022
GhNAC177	scaffold2414.1	68448	69522	No group
GhNAC178	At02	51104751	51113208	No group
GhNAC179	At08	2027998	2029345	OsNAC7
GhNAC180	Dt01	34993430	34994362	TERN
GhNAC181	Dt01	22849854	22853976	NAC1
GhNAC182	Dt06	44579056	44580416	ONAC022
GhNAC183	At11	60088385	60090470	No group
GhNAC184	Dt08	54272911	54274252	OsNAC7
GhNAC185	scaffold3586.1	43352	45157	ONAC003
GhNAC186	Dt01	27181569	27182995	OsNAC7
GhNAC187	At03	14047267	14048598	OsNAC7
GhNAC188	At09	32234351	32237417	No group
GhNAC189	At03	86317649	86318892	ATAF
GhNAC190	At13	73412057	73413389	ONAC022
GhNAC191	Dt10	47662688	47664116	ONAC003
GhNAC192	scaffold1782.1	188208	191764	No group
GhNAC193	At05	11594221	11595811	OsNAC7
GhNAC194	Dt07	17611333	17613253	OsNAC7
GhNAC195	At10	94546011	94547238	NAP
GhNAC196	At12	26984052	26985191	TERN
GhNAC197	Dt11	50131747	50140759	ONAC022
GhNAC198	At10	58201435	58202864	ONAC003

GhNAC199	At03	82184892	82186117	NAP
GhNAC200	At11	59426888	59428886	No group
GhNAC201	At03	23446486	23449177	TIP
GhNAC202	At12	74220386	74221746	ONAC022
GhNAC203	Dt05	36228400	36229461	NAP
GhNAC204	scaffold3682.1	5443	6715	NAM
GhNAC205	Dt13	15813266	15814682	OsNAC7
GhNAC206	Dt01	18231915	18233247	ONAC022
GhNAC207	scaffold5566.1	13718	16622	No group
GhNAC208	scaffold5036.1	14326	15819	OsNAC7
GhNAC209	scaffold1864.1	247559	249402	No group
GhNAC210	scaffold7841.1	4250	5591	OsNAC7
GhNAC211	scaffold3858.1	64614	66037	OsNAC7

TABLE S7 Orthologous gene pairs of NAC proteins in *G.hirsutum*, *G.arboreum* and *G.raimondii*.

**Genes of At subgenome**	**Orthologous genes in *G. arboreum***	**Orthologous genes in *G. raimondii***	**Genes of Dt subgenome**	**Subfamily**
-	GaNAC11	GrNAC58	-	ANAC011
GhNAC69	GaNAC52	GrNAC20	-	ANAC011
-	GaNAC87	GrNAC29	GhNAC110	ANAC011
GhNAC57	GaNAC55	GrNAC127	GhNAC116	ANAC011
-	GaNAC43	GrNAC111	GhNAC13	ANAC011
GhNAC34	-	GrNAC118	GhNAC150	ANAC011
GhNAC61	GaNAC14	GrNAC74	GhNAC33	ANAC011
GhNAC15	GaNAC28	GrNAC42	GhNAC51	ANAC011
-	GaNAC38	GrNAC84	GhNAC60	ANAC011
GhNAC61	GaNAC54	GrNAC31	GhNAC8	ANAC011
-	GaNAC35	-	-	ANAC063
-	GaNAC61	GrNAC116	-	ANAC063
GhNAC189	GaNAC121	GrNAC132	GhNAC115	ATAF
GhNAC103	GaNAC71	GrNAC1	GhNAC139	ATAF
GhNAC167	GaNAC98	GrNAC63	GhNAC85	ATAF
-	GaNAC33	GrNAC10	GhNAC91	AtNAC3
-	GaNAC60	GrNAC60	GhNAC91	AtNAC3
-	GaNAC15	GrNAC40	-	NAC1
GhNAC38	GaNAC84	GrNAC97	-	NAC1
GhNAC181	GaNAC106	GrNAC89	GhNAC146	NAC1
GhNAC78	GaNAC112	GrNAC72	GhNAC67	NAC1
GhNAC30	GaNAC30	GrNAC21	-	NAC2
-	GaNAC118	GrNAC26	GhNAC148	NAC2
GhNAC161	GaNAC110	GrNAC87	GhNAC174	NAC2

GhNAC141/GhNAC204	GaNAC57	GrNAC13	-	NAM
-	GaNAC128	GrNAC110	GhNAC12	NAM
GhNAC154	-	GrNAC138	GhNAC132	NAM
GhNAC168	GaNAC131	GrNAC133	GhNAC143	NAM
GhNAC149	GaNAC125	GrNAC81	GhNAC162	NAM
GhNAC37	GaNAC65	GrNAC105	GhNAC23	NAM
GhNAC89	GaNAC72	GrNAC135	GhNAC27	NAM
-	GaNAC75	GrNAC38	GhNAC58	NAM
-	GaNAC32	GrNAC8	GhNAC58	NAM
GhNAC164	GaNAC78	GrNAC136	GhNAC65	NAM
GhNAC1	GaNAC17	GrNAC100	-	NAP
GhNAC131	GaNAC119	GrNAC88	-	NAP
GhNAC71	GaNAC135	GrNAC114	-	NAP
GhNAC203	GaNAC107	GrNAC128	GhNAC145	NAP
GhNAC195	GaNAC139	GrNAC142	GhNAC199	NAP
GhNAC95	GaNAC89	GrNAC16	GhNAC20	NAP
GhNAC88	GaNAC73	GrNAC134	GhNAC26	NAP
GhNAC56	GaNAC31	GrNAC23	GhNAC44	NAP
GhNAC101	GaNAC126	GrNAC65	GhNAC64	NAP
GhNAC165	GaNAC79	GrNAC137	GhNAC66	NAP
GhNAC160	GaNAC59	GrNAC59	GhNAC76	NAP
GhNAC160	GaNAC34	GrNAC11	GhNAC92	NAP
-	GaNAC114	-	-	No group
-	GaNAC19	-	-	No group
-	GaNAC26	-	-	No group
-	GaNAC103	-	-	No group

-	GaNAC94	GrNAC14	-	No group
-	GaNAC23	GrNAC15	-	No group
-	-	GrNAC17/GrNAC18	-	No group
-	GaNAC5	GrNAC54	-	No group
-	GaNAC95	GrNAC67/GrNAC68	-	No group
-	GaNAC104/GaNAC124	GrNAC7	-	No group
-	-	GrNAC75	-	No group
-	-	GrNAC76	-	No group
-	GaNAC18	GrNAC77/GrNAC78	-	No group
-	-	GrNAC79	-	No group
-	GaNAC105	GrNAC96	-	No group
GhNAC108	GaNAC96	-	-	No group
GhNAC59	GaNAC97	GrNAC35	-	No group
GhNAC9/GhNAC100	GaNAC41	-	-	No group
GhNAC144	GaNAC22	GrNAC115	GhNAC102	No group
GhNAC107	GaNAC58	GrNAC69/GrNAC70	GhNAC109	No group
GhNAC169	GaNAC9	GrNAC109	GhNAC11	No group
GhNAC43	GaNAC69	GrNAC130	GhNAC111	No group
GhNAC43	GaNAC102	GrNAC94	GhNAC111	No group
GhNAC158	GaNAC108	-	GhNAC117	No group
GhNAC163	GaNAC6	GrNAC33	GhNAC122	No group
-	GaNAC67	GrNAC123	GhNAC123	No group
-	GaNAC68	GrNAC124	GhNAC124	No group
GhNAC200	GaNAC85	GrNAC104	GhNAC128	No group
GhNAC90	-	GrNAC62	GhNAC133	No group
-	GaNAC24	GrNAC98	GhNAC151	No group

GhNAC130	GaNAC86	GrNAC120	GhNAC170	No group
GhNAC188	GaNAC111	GrNAC86	GhNAC173	No group
-	GaNAC64	GrNAC39	GhNAC21	No group
GhNAC35	GaNAC45	GrNAC107	GhNAC22	No group
GhNAC207	GaNAC42	GrNAC64	GhNAC25	No group
-	GaNAC21	GrNAC80	GhNAC28/GhNAC29	No group
GhNAC177/GhNAC119	GaNAC2	GrNAC56	GhNAC31	No group
GhNAC99	GaNAC1	GrNAC55	GhNAC32	No group
-	GaNAC4	GrNAC95	GhNAC40	No group
GhNAC63	GaNAC8	GrNAC125	GhNAC46	No group
GhNAC104	GaNAC37	GrNAC32	GhNAC54	No group
GhNAC183	GaNAC142	GrNAC22	GhNAC55	No group
GhNAC157	GaNAC3	GrNAC57	GhNAC7	No group
GhNAC178	GaNAC138	GrNAC12	GhNAC75	No group
GhNAC192	GaNAC63	GrNAC92	GhNAC80	No group
-	GaNAC109	GrNAC85	GhNAC82	No group
GhNAC155	GaNAC117	GrNAC25	GhNAC86	No group
GhNAC166	GaNAC53	GrNAC93	GhNAC94	No group
GhNAC129	-	-	-	No group
GhNAC209	-	-	-	No group
-	GaNAC113	GrNAC3	-	ONAC022
-	GaNAC82	GrNAC30	-	ONAC022
-	GaNAC120	GrNAC4	GhNAC126	ONAC022
GhNAC112	GaNAC70	GrNAC66	GhNAC134	ONAC022
GhNAC190/GhNAC206	GaNAC140	GrNAC112	GhNAC135	ONAC022
GhNAC176	GaNAC115	GrNAC5	GhNAC182	ONAC022

GhNAC172	GaNAC136	GrNAC140	GhNAC142	ONAC022
GhNAC172	GaNAC136	GrNAC101	GhNAC197	ONAC022
-	GaNAC25	GrNAC82	GhNAC24	ONAC022
GhNAC19	GaNAC92	GrNAC46	GhNAC47	ONAC022
GhNAC18	GaNAC91	GrNAC45	GhNAC48	ONAC022
GhNAC17	GaNAC90	GrNAC44	GhNAC49	ONAC022
GhNAC202	GaNAC134	GrNAC129	GhNAC97/GhNAC98	ONAC022
-	GaNAC77	GrNAC131	-	OsNAC003
GhNAC156	GaNAC44	GrNAC126	-	OsNAC003
GhNAC185	GaNAC50	GrNAC113	-	OsNAC003
GhNAC36	GaNAC83	GrNAC106	-	OsNAC003
GhNAC45	GaNAC129	GrNAC34	-	OsNAC003
GhNAC198	GaNAC137	GrNAC141	GhNAC191	OsNAC003
GhNAC159	GaNAC20	GrNAC119	GhNAC42	OsNAC003
GhNAC175	GaNAC123	GrNAC2	GhNAC70	OsNAC003
-	-	GrNAC108	-	OsNAC7
GhNAC106	GaNAC36	GrNAC83	-	OsNAC7
GhNAC205	GaNAC132	GrNAC61	-	OsNAC7
GhNAC72	GaNAC16	GrNAC24	-	OsNAC7
GhNAC118	GaNAC10	-	GhNAC10	OsNAC7
-	GaNAC80	GrNAC27	GhNAC136	OsNAC7
GhNAC179	GaNAC101	GrNAC50	GhNAC138	OsNAC7
-	GaNAC27	GrNAC19	GhNAC14	OsNAC7
GhNAC53/GhNAC210	GaNAC56	GrNAC52	GhNAC184	OsNAC7
-	GaNAC81	GrNAC117	GhNAC194	OsNAC7
GhNAC193	GaNAC88	GrNAC103	GhNAC2	OsNAC7

-	GaNAC66	GrNAC122	GhNAC208	OsNAC7
GhNAC105	GaNAC7	-	GhNAC5	OsNAC7
GhNAC16	GaNAC29	GrNAC43	GhNAC50	OsNAC7
GhNAC62	GaNAC40	GrNAC47	GhNAC73	OsNAC7
GhNAC186	GaNAC130	GrNAC91	GhNAC81	OsNAC7
GhNAC79/GhNAC171	GaNAC51	GrNAC139	GhNAC83	OsNAC7
GhNAC187	GaNAC116	GrNAC99	GhNAC84	OsNAC7
-	GaNAC74	GrNAC28	GhNAC87	OsNAC7
GhNAC211	GaNAC141	GrNAC9	GhNAC96	OsNAC7
-	-	-	GhNAC93	OsNAC7
GhNAC113	GaNAC99	GrNAC49	GhNAC121	OsNAC8
GhNAC41	GaNAC62	GrNAC102	GhNAC127	OsNAC8
GhNAC152	GaNAC47	GrNAC6	GhNAC4	SENU5
GhNAC153	GaNAC48	GrNAC6	GhNAC4	SENU5
-	GaNAC93	GrNAC41	GhNAC68	SENU5
GhNAC114	GaNAC100	GrNAC48	GhNAC120	TERN
GhNAC196	GaNAC133	GrNAC121	GhNAC137	TERN
GhNAC180	GaNAC46	GrNAC90	GhNAC140	TERN
GhNAC39	GaNAC49	GrNAC71	GhNAC77	TERN
GhNAC52	GaNAC76	GrNAC51	-	TIP
GhNAC125	GaNAC122	GrNAC73	GhNAC147	TIP
GhNAC201	GaNAC127	GrNAC36	GhNAC3	TIP
-	GaNAC12/GaNAC13	GrNAC53	GhNAC6	TIP
-	GaNAC39	GrNAC37	GhNAC74	TIP

-represents no orthologous genes were found.

TABLE S8 The duplicated GaNAC, GrNAC and GhNAC and its corresponding orthologous genes in three other dicots.

Species	Duplicated gene 1	Duplicated gene 2	Subfamily	Locus name of its corresponding orthologous gene in some dicots		
				V.vinifera	***A.thaliana***	***T.cacao***
G.arboreum	GaNAC102	GaNAC69	No group	GSVIVT01029709001	AT5G64530	Thecc1EG007543
	GaNAC104	GaNAC124	No group	-	-	-
	GaNAC12	GaNAC13	TIP	GSVIVT01038666001	AT5G24590	Thecc1EG015535
	GaNAC33	GaNAC60	AtNAC3	GSVIVT01014403001	AT1G52890	Thecc1EG031364
	GaNAC34	GaNAC59	NAP	GSVIVT01014405001	AT3G15510	Thecc1EG031363
	GaNAC47	GaNAC48	SENU5	GSVIVT01000940001	AT5G13180	Thecc1EG008477
	GaNAC75	GaNAC32	NAM	GSVIVT01014287001	AT5G53950	Thecc1EG031619
	GaNAC90	GaNAC91	ONAC022	GSVIVT01020834001	AT2G43000	Thecc1EG032389/Thecc1EG032393
G.raimondii	GrNAC10	GrNAC60	AtNAC3	GSVIVT01014403001	AT1G52890	Thecc1EG031364
	GrNAC101	GrNAC140	ONAC022	GSVIVT01020387001	AT2G43000	Thecc1EG029321
	GrNAC11	GrNAC59	NAP	GSVIVT01014405001	AT3G15510	Thecc1EG031363
	GrNAC130	GrNAC94	No group	GSVIVT01029709001	AT5G64530	Thecc1EG007543
	GrNAC17	GrNAC18	No group	-	-	Thecc1EG020125
	GrNAC31	GrNAC74	ANAC011	GSVIVT01029392001	AT3G17730	Thecc1EG044911
	GrNAC38	GrNAC8	NAM	GSVIVT01014287001	AT5G53950	Thecc1EG031619
	GrNAC44	GrNAC45	ONAC022	GSVIVT01020834001	AT2G43000	Thecc1EG032389/Thecc1EG032393
	GrNAC54	GrNAC96	No group	-	-	-
	GrNAC67	GrNAC68	No group	-	-	Thecc1EG017070/Thecc1EG017048
	GrNAC70	GrNAC69	No group	-	-	Thecc1EG042655
	GrNAC77	GrNAC78	No group	-	-	-
G.hirsutum	GhNAC100	GhNAC9	No group	-	-	Thecc1EG033799
	GhNAC141	GhNAC204	NAM	GSVIVT01007982001	AT5G61430	Thecc1EG014073
	GhNAC153	GhNAC152	SENU5	GSVIVT01000940001	AT5G13180	Thecc1EG008477

	GhNAC17	GhNAC18	ONAC022	GSVIVT01020834001	AT2G43000	Thecc1EG032389/Thecc1EG032393
	GhNAC177	GhNAC119	No group	GSVIVT01023921001	AT2G17040	Thecc1EG006499
	GhNAC183	GhNAC200	No group	GSVIVT01023123001	AT2G02450	Thecc1EG027945
	GhNAC190	GhNAC206	ONAC022	GSVIVT01019993001	AT1G26870	Thecc1EG011718
	GhNAC197	GhNAC142	ONAC022	GSVIVT01020387001	AT2G43000	Thecc1EG029321
	GhNAC28	GhNAC29	No group	GSVIVT01013671001	AT1G34180	Thecc1EG036587
	GhNAC49	GhNAC48	ONAC022	GSVIVT01020834001	AT2G43000	Thecc1EG032389/Thecc1EG032393
	GhNAC53	GhNAC210	OsNAC7	GSVIVT01019670001	AT2G46770	Thecc1EG015621
	GhNAC76	GhNAC92	NAP	GSVIVT01014405001	AT3G15510	Thecc1EG031363
	GhNAC79	GhNAC171	OsNAC7	GSVIVT01018809001	AT2G18060	Thecc1EG000235
	GhNAC97	GhNAC98	ONAC022	GSVIVT01019993001	AT1G26870	Thecc1EG011718

-represents no orthologous genes were found.

TABLE S9 Information of synetic blocks predicted among *G. hirsutum*, *G. arboretum* and *G. raimondii* NAC homologs.

Blocks	*G. raimondii* ortholog in *G. hirsutum*		*G. raimondii*		*G. arboretum*		*G. arboretum* ortholog in *G. hirsutum*	
	**Chromosome**	**Name**	**Chromosome**	**Name**	**Chromosome**	**Name**	**Chromosome**	**Name**
1	-	-	DD01	GrNAC42\ GrNAC43	AA03	GaNAC29\ GaNAC28	Dt05	GhNAC15\ GhNAC16
2	At10	GhNAC65\ GhNAC66	DD07	GrNAC137\ GrNAC136	AA04	GaNAC79\ GaNAC78	At01	GhNAC164\ GhNAC165
3	Dt07	GhNAC124\ GhNAC123	DD07	GrNAC123\ GrNAC124	AA04	GaNAC67\ GaNAC66	-	-
4	-	-	DD01	GrNAC44\ GrNAC45\ GrNAC46	AA05	GaNAC90\ GaNAC91\ GaNAC92	Dt05	GhNAC17\ GhNAC18\ GhNAC19
5	-	-	DD08	GrNAC50\ GrNAC139	AA06	GaNAC51\ GaNAC101	-	-
6	-	-	DD08	GrNAC55\ GrNAC56	AA07	GaNAC1\ GaNAC2	-	-
7	-	-	DD03	GrNAC29\ GrNAC28	AA07	GaNAC87\ GaNAC74	-	-
8	Dt09	GhNAC174\ GhNAC173	DD09	GrNAC86\ GrNAC87	AA10	GaNAC111\ GaNAC110	-	-
9	-	-	DD09	GrNAC60\ GrNAC59	AA10	GaNAC59\ GaNAC60	-	-
10	Dt06	GhNAC74\ GhNAC73	DD06	GrNAC37\ GrNAC47	AA11	GaNAC39\ GaNAC40	-	-
11	Dt06	GhNAC120\	DD06	GrNAC48\	AA11	GaNAC100\	At06	GhNAC114\

		GhNAC121		GrNAC49		GaNAC99		GhNAC113
12	Dt06	GhNAC64\ GhNAC132	DD06	GrNAC138\ GrNAC65	-	-	-	-
13	Dt13	GhNAC27\ GhNAC26	DD07	GrNAC135\ GrNAC134	AA13	GaNAC72\ GaNAC73	At13	GhNAC89\ GhNAC88
14	-	-	DD13	GrNAC58\ GrNAC112	AA13	GaNAC11\ GaNAC140	-	-
15	Dt01	GhNAC80\ GhNAC81\ GhNAC146	DD01	GrNAC89\ GrNAC91\ GrNAC92	-	-	-	-
16	Dt07	GhNAC11\ GhNAC12\ GhNAC13	DD07	GrNAC109\ GrNAC110\ GrNAC111	-	-	-	-
17	Dt08	GhNAC44\ GhNAC70	DD08	GrNAC2\ GrNAC23	-	-	-	-
18	-	-	-	-	AA08	GaNAC136\ GaNAC131	At10	GhNAC168\ GhNAC172
19	-	-	-	-	AA13	GaNAC72\ GaNAC73	At13	GhNAC89\ GhNAC88
20	-	-	-	-	AA06	GaNAC9\ GaNAC10	At07	GhNAC169\ GhNAC118
21	-	-	-	-	AA08	GaNAC47\ GaNAC48	At05	GhNAC152\ GhNAC153

-represents no orthologous genes were found

TABLE S10 PCR primers used in this study.

Name	Forward primer (5'-3')	Reverse primer (5'-3')
GaNAC7	TCCGACTGGAACGAGGACTA	TCCTTCCTCCAAGGTCTTCCT
GaNAC11	TCCCATGACTCTCCCTCCTG	ATCCCTGGGGCTGTAGAAGT
GaNAC14	CCGAATGGGTTCAGGACGAA	TCCTGCCAATAACCACTTCTCT
GaNAC28	AAAATTACCGGTCGCCCCAT	TCCGGTGGCTTTCCAATACC
GaNAC31	CATGGGTCCAGTTTCCCATCT	CCCCAAGCTGTAGCTTCTCAA
GaNAC33	CAAGCAGAGAGCAAAGCACG	CCACCAAGACTCCCCCAATC
GaNAC34	ACTGGACCGACGATGAAACC	GACCCCAACATTGCTGCTTG
GaNAC43	CTCGAGAACGAATCGAGCCA	AGGGTCTTACGATAGCCGGT
GaNAC47	CTTGGGACTTGCCAGGTGAT	ATGCATGATCCAGTCGGTCC
GaNAC48	GGGTTCCGCTTCCATCCTAC	ATAGAGGCAGGTAAGGGCCA
GaNAC50	AGCCGCTTCCTACACCAAAA	TGAAGCTTGCGCAGATCAGA
GaNAC54	TTGGACCCCGAGATCGGAAA	TGGGAAGCAACTCTCCTGTC
GaNAC59	CAGAGCCACAAGCAAACAAGAA	GTTGTAATGAAGGACTTTGTCGT
GaNAC60	AAGTATCCGAACGGGTCACG	CAACTTTTCGGCCATCGGTC
GaNAC61	GGCGAGTGGAAGACCAAAGA	AAGAGAAACTCGGCACAGGG
GaNAC62	GACAAAGAAGCTGGAGGGCT	GGTTGCGCGTCTACTTTGTG
GaNAC75	GTGTCCTGTTTCTCCACCGT	AGTTGACAGATGAGCCACCG
GaNAC77	GGAGCTTGTGGTGTCGAAGA	CTCGATAAAGCAGCGGCAAC
GaNAC83	TCCATGGTGTTGAATGTGAGGA	TCCAAGCAATTCATCTCGGAA
GaNAC84	CGGTGATCGACCCAAGAACA	CCTTAGGAGGCATATGCGGG
GaNAC89	TGGGAGGGGTGGAGTGTATT	CTGCTTCGTCTTCCTCTTGGT
GaNAC91	GTTCTGGAAAGCAACGGGCA	GGTCATTGTGGGATTGGGGA
GaNAC93	TGTGCTGAGATTGCCTCCTG	GGCAAGCAAGCACTTTTCGT
GaNAC100	CCAAGACAAGAGAGGGAGGC	ACATATGGGTCGGACAGTGC
GaNAC106	GACGGGGAAGGATCGAATGG	TTCCATTGGGAGCTCGGTTC
GaNAC118	ATGCAAGGTTGAGAGACCCG	TGCCGTCTTCTCTTGTACCG
GaNAC120	AAGGCCAAATAGGGTGACCG	CGGCACAGCCTCGATAGTAG
GaNAC121	GCAGCTACGGCGATTGGTAA	GAATTCCCGAGACACCGCAT
GaNAC122	TGCCTACCGGATTTCGGTTC	TTCCTGTCGAGTGGGCAAAA
GaNAC139	ACCGCAGTCTCTTCAAGCTC	TCCAGTACTGTGGACCTGCT
GrNAC4	AAGGCCAAATAGGGTGACCG	CGGCACAGCCTCGATAGTAG
GrNAC6	GGGTTCCGCTTCCATCCTAC	ATAGAGGCAGGTAAGGGCCA
GrNAC10	CAAGCAGAGAGCAAAGCACG	CCACCAAGACTCCCCCAATC
GrNAC11	ACTGGACCGACGATGAAACC	GACCCCAACATTGCTGCTTG
GrNAC16	CAGCCCCAGAAGTCCTGTTC	GCTTTTGGGGAAACCTGCTG
GrNAC19	CAGCAGCCAAACTCAACCAC	GGGAGGGTAGTGAAGGGCTA
GrNAC26	ATGCAAGGTTGAGAGACCCG	TGCCGTCTTCTCTTGTACCG
GrNAC38	GTGTCCTGTTTCTCCACCGT	AGTTGACAGATGAGCCACCG
GrNAC41	TGTGCTGAGATTGCCTCCTG	GGCAAGCAAGCACTTTTCGT
GrNAC42	AATGAGTCTCCCTCCCGGTT	GGCGACCGGTGATTTTGTTG
GrNAC44	GAACAGTGTAAGACCAAACCGA	ATGGCTTCACCGCTTTTGAC
GrNAC45	CAGATGAAGAGCTCGTCGGG	TTCCAGAACCCCGACTCTGT

GrNAC48	CAAGAGAGGGAAGCTCGTGG	ACATATGGGTCGGACAGTGC
GrNAC58	TCCCATGACTCTCCCTCCTG	ATCCCTGGGGCTGTAGAAGT
GrNAC59	CCTCAATAGAGCCACAAGCAA	AGGACTTTCTGGTAGCTGGC
GrNAC60	AAGTATCCGAACGGGTCACG	CAACTTTTCGGCCATCGGTC
GrNAC73	TGTGAGCCGTGGGATTTACC	TCCTTACCCGTAGCCTTCCA
GrNAC89	GACGGGAAAGGATCGAATGGT	GCGTTGCTGTTTTCTCCTCC
GrNAC97	CGGTGATCGACCCAAGAACA	CCTTAGGAGGCATATGCGGG
GrNAC102	GACAAAGAAGCTGGAGGGCT	GGTTGCGCGTCTACTTTGTG
GrNAC106	ATGTGCTAATCCGCCAGAGG	GTAGGTCCCACCGTTGACTG
GrNAC111	CTCGAGAACGAATCGAGCCA	AGGGTCTTACGATAGCCGGT
GrNAC113	AGCCGCTTCCTACACCAAAA	TGAAGCTTGCGCAGATCAGA
GrNAC116	GGCGAGTGGAAGACCAAAGA	AAGAGAAACTCGGCACAGGG
GrNAC117	GGATACGAGGAGCAAAGCGA	CCCAGCCTTCTTCAAGGGTC
GrNAC131	GGAGCTTGTGGTGTCGAAGA	CTCGATAAAGCAGCGGCAAC
GrNAC132	TGGCTTTCGGTTCCATCCAA	GACGGCGATAGACTGAGACG
GrNAC136	CGACGAACTCATCGATTTGCC	ACCAATAGCGCTTGCACTGA
GrNAC142	AAAAGGAAGGTGGCTTCGGT	TTGGAAGCTCCCATGGATCG
GhNAC5	AGTATCCGACAGGGACGAGG	ACCCATCCTTCCTCCAAGGT
GhNAC8	TTGGACCCCGAGATCGGAAA	TGGGAAGCAACTCTCCTGTC
GhNAC13	CTCGAGAACGAATCGAGCCA	AGGGTCTTACGATAGCCGGT
GhNAC14	CAGCAGCCAAACTCAACCAC	GGGAGGGTAGTGAAGGGCTA
GhNAC15	AAAATTACCGGTCGCCCCAT	TCCGGTGGCTTTCCAATACC
GhNAC18	GTTCTGGAAAGCAACGGGCA	GGTCATTGTGGGATTGGGGA
GhNAC19	TGGCAATGGTGTACATGGCT	ATTCCCTTCTGCAACTCCCG
GhNAC20	CAGCCCCAGAAGTCCTGTTC	GCTTTTGGGGAAACCTGCTG
GhNAC27	CGCTTCCATGGTGCTCTACC	ACACGTGCTCGGATTGGTTA
GhNAC36	TCCATGGTGTTGAATGTGAGGA	TCCAAGCAATTCATCTCGGAA
GhNAC38	CGGTGATCGACCCAAGAACA	CCTTAGGAGGCATATGCGGG
GhNAC41	GACAAAGAAGCTGGAGGGCT	GGTTGCGCGTCTACTTTGTG
GhNAC44	AAGCCATGCCCTGTTTCGAT	ACCCCATTCGGGTACTTTCG
GhNAC48	CAGATGAAGAGCTCGTCGGG	TTCCAGAACCCCGACTCTGT
GhNAC49	GAACAGTGTAAGACCAAACCGA	ATGGCTTCACCGCTTTTGAC
GhNAC51	AATGAGTCTCCCTCCCGGTT	GGCGACCGGTGATTTTGTTG
GhNAC56	CATGGGTCCAGTTTCCCATCT	CCCCAAGCTGTAGCTTCTCAA
GhNAC58	GGAAGACTGGCCGTATGAGT	TGGTGGGATCAAGGAGAGGT
GhNAC60	CCCCTTTAGGGGATCGAACG	CTCCTCGAGCATCACTTGCT
GhNAC65	CGACGAACTCATCGATTTGCC	ACCAATAGCGCTTGCACTGA
GhNAC68	TGTGCTGAGATTGCCTCCTG	GGCAAGCAAGCACTTTTCGT
GhNAC76	CCTCAATAGAGCCACAAGCAA	AGGACTTTCTGGTAGCTGGC
GhNAC89	CCGCTCCTGGATACAACCAA	TGTTGTTCATCTGGGGTGGG
GhNAC91	CAAGCAGAGAGCAAAGCACG	CCACCAAGACTCCCCCAATC
GhNAC92	ACTGGACCGACGATGAAACC	GACCCCAACATTGCTGCTTG
GhNAC95	TGGGAGGGGTGGAGTGTATT	CTGCTTCGTCTTCCTCTTGGT
GhNAC105	TCCGACTGGAACGAGGACTA	TCCTTCCTCCAAGGTCTTCCT

GhNAC114	CCAAGACAAGAGAGGGAGGC	ACATATGGGTCGGACAGTGC
GhNAC115	TGGCTTTCGGTTCCATCCAA	GACGGCGATAGACTGAGACG
GhNAC120	CAAGAGAGGGAAGCTCGTGG	ACATATGGGTCGGACAGTGC
GhNAC125	TGCCTACCGGATTTCGGTTC	TTCCTGTCGAGTGGGCAAAA
GhNAC126	AAGGCCAAATAGGGTGACCG	CGGCACAGCCTCGATAGTAG
GhNAC127	GGATCAGAGGAGACGTGAGC	TGCAGCCACCACATAGTCTG
GhNAC146	GACGGGAAAGGATCGAATGGT	GCGTTGCTGTTTTCTCCTCC
GhNAC147	TGTGAGCCGTGGGATTTACC	TCCTTACCCGTAGCCTTCCA
GhNAC148	ATGCAAGGTTGAGAGACCCG	TGCCGTCTTCTCTTGTACCG
GhNAC160	CAGAGCCACAAGCAAACAAGAA	GTTGTAATGAAGGACTTTGTCGT
GhNAC164	AGACGAACTCATCGATTTGCC	CCCAAGGCTCACACTTGTTC
GhNAC181	GACGGGGAAGGATCGAATGG	TTCCATTGGGAGCTCGGTTC
GhNAC185	AGCCGCTTCCTACACCAAAA	TGAAGCTTGCGCAGATCAGA
GhNAC189	GCAGCTACGGCGATTGGTAA	GAATTCCCGAGACACCGCAT
GhNAC194	GGATACGAGGAGCAAAGCGA	CCCAGCCTTCTTCAAGGGTC
GhNAC195	ACCGCAGTCTCTTCAAGCTC	TCCAGTACTGTGGACCTGCT
GhNAC199	AAAAGGAAGGTGGCTTCGGT	TTGGAAGCTCCCATGGATCG
EF1α	AGACCACCAAGTACTACTGCAC	CCACCAATCTTGTACACATCC
