# Supplementary material for: Asymmetric Evolution and Expansion of the NAC Transcription Factor in Polyploidized Cotton
Source: Front Plant Sci. 2018 Jan 30;9:47. doi: 10.3389/fpls.2018.00047 (PMC5797638; doi:10.3389/fpls.2018.00047)
Supplement: Figure S1 — Evolutionary analysis of the NAC family in G. raimondii, G. arboreum, G. hirsutum, A. thaliana, T. cacao, and V. vinifera with the MEGA program. Amino acid sequences were aligned with ClustalW, and the neighbor-joining (NJ) tree was generated through the MEGA program. The numbers in the clades are the bootstrap values. The subfamilies within the NAC family are grouped by colors. [file Image1.PDF]

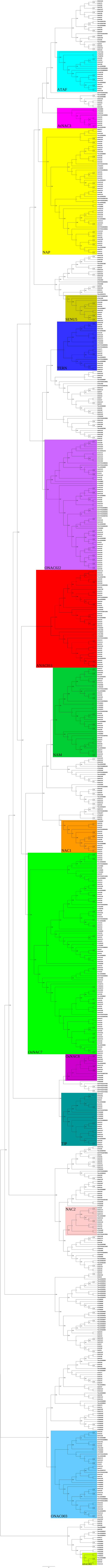

ATAF
AtNAC3
NAP
SENU5
TERN
ONAC022
ANAC011
NAM
NAC1
OsNAC7
OsNAC8
TIP
NAC2
ONAC003
ANAC063
